# Neurobehavioral dysfunction in a mouse model of Down syndrome: upregulation of cystathionine β-synthase, H_2_S overproduction, altered protein persulfidation, synaptic dysfunction, endoplasmic reticulum stress, and autophagy

**DOI:** 10.1007/s11357-024-01146-8

**Published:** 2024-04-01

**Authors:** Theodora Panagaki, Lucia Janickova, Dunja Petrovic, Karim Zuhra, Tamás Ditrói, Eszter P. Jurányi, Olivier Bremer, Kelly Ascenção, Thilo M. Philipp, Péter Nagy, Milos R. Filipovic, Csaba Szabo

**Affiliations:** 1 Chair of Pharmacology, Section of Science and Medicine, University of Fribourg, Fribourg, Switzerland; 2 Leibniz-Institut Für Analytische Wissenschaften-ISAS-E.V., Dortmund, Germany; 3 Department of Molecular Immunology and Toxicology and the National Tumor Biology Laboratory, National Institute of Oncology, Budapest, Hungary; 4 Doctoral School of Semmelweis University, Semmelweis University, Budapest, Hungary; 5 Department of Anatomy and Histology, HUN-REN–UVMB Laboratory of Redox Biology Research Group, University of Veterinary Medicine, Budapest, Hungary; 6 Chemistry Institute, University of Debrecen, Debrecen, Hungary

**Keywords:** Metabolism, Brain, Astrocytes, Gliosis, Gasotransmitters, Persulfidation, Cognition

## Abstract

Down syndrome (DS) is a genetic condition where the person is born with an extra chromosome 21. DS is associated with accelerated aging; people with DS are prone to age-related neurological conditions including an early-onset Alzheimer’s disease. Using the Dp(17)3Yey/ + mice, which overexpresses a portion of mouse chromosome 17, which encodes for the transsulfuration enzyme cystathionine β-synthase (CBS), we investigated the functional role of the CBS/hydrogen sulfide (H_2_S) pathway in the pathogenesis of neurobehavioral dysfunction in DS. The data demonstrate that CBS is higher in the brain of the DS mice than in the brain of wild-type mice, with primary localization in astrocytes. DS mice exhibited impaired recognition memory and spatial learning, loss of synaptosomal function, endoplasmic reticulum stress, and autophagy. Treatment of mice with aminooxyacetate, a prototypical CBS inhibitor, improved neurobehavioral function, reduced the degree of reactive gliosis in the DS brain, increased the ability of the synaptosomes to generate ATP, and reduced endoplasmic reticulum stress. H_2_S levels in the brain of DS mice were higher than in wild-type mice, but, unexpectedly, protein persulfidation was decreased. Many of the above alterations were more pronounced in the female DS mice. There was a significant dysregulation of metabolism in the brain of DS mice, which affected amino acid, carbohydrate, lipid, endocannabinoid, and nucleotide metabolites; some of these alterations were reversed by treatment of the mice with the CBS inhibitor. Thus, the CBS/H_2_S pathway contributes to the pathogenesis of neurological dysfunction in DS in the current animal model.

**Supplementary Information:**

The online version contains supplementary material available at 10.1007/s11357-024-01146-8.

## Introduction

Hydrogen sulfide (H_2_S), an endogenous gaseous transmitter, has been implicated in a wide range of processes in physiology as well as pathophysiology in mammals [1, 2]. Three principal enzymatic sources of H_2_S exist in mammalian cells and tissues: cystathionine β-synthase (CBS), cystathionine γ-lyase (CSE), and 3-mercaptopyruvate sulfurtransferase (3-MST), but emerging data suggest an additional role of cysteinyl-tRNA synthetase 2 (CARS2) [1, 2]. H_2_S levels in cells and tissues are dynamically regulated, with the major enzymes involved in its catabolism being rhodanese or thiosulfate sulfurtransferase (TST), ethylmalonic encephalopathy 1 protein (ETHE1), sulfur dioxygenase (SQR), and sulfide:quinone oxidoreductase (SUOX) [1, 2].

The gene that encodes CBS is located on chromosome 21q22 in humans. Down syndrome (DS) is a common genetic disorder due to the presence of an additional third copy of chromosome 21. DS is associated with accelerated aging; people with DS are prone to age-related neurological conditions including early-onset Alzheimer’s disease [3–6]. Over the last 20 years, starting with biochemical observations of Lejeune [7] and clinical observations of Kamoun et al. [8, 9], the concept emerged in the pathogenesis of DS, which suggests that overproduction of H_2_S, at least in part due to a gene dosage effect, plays a role in the neuropathological alterations associated with DS. The Kamoun hypothesis—stipulating that high concentrations of H_2_S in DS cells and tissues promote cell dysfunction [10]—has been experimentally confirmed in recent years in vitro and in vivo [11]. A growing body of experimental data indicates that excess H_2_S in DS suppresses mitochondrial complex IV activity; diverts the physiological, aerobic, mitochondrial ATP generation into the anaerobic pathway; and leads to cell dysfunction [12–17].

In contrast to humans, where cbs is located on chromosome 21, in mice, cbs is located on chromosome 17. From in vivo studies in various models of DS mice and DS rats that incorporate an extra chromosome or chromosomal fragment encoding CBS, it appears that the CBS/H_2_S pathway contributes to cognitive defects and even to the alterations in the spontaneous electrical activity (electroencephalogram, EEG) of the brain [12, 14].

The pharmacological options to inhibit CBS enzymatic activity are currently limited. Aminooxyacetic acid (AOAA) is a compound that has been used for several decades. It is a potent inhibitor of CBS, although it also has additional inhibitory effects on CSE, as well as other pyridoxal-phosphate-dependent enzymes. It has good brain penetration, and it is accepted as a pharmacological tool to probe the role of the CBS/H_2_S pathway in various models of health and disease [18–20]. Using this compound, we have recently demonstrated the role of the CBS pathway in neurological and EEG alterations in a rat model of DS [14] and in the biochemical and metabolic alterations associated with DS in cellular models [13, 15].

To extend these findings, in the current study, we have now evaluated the effect of AOAA in a mouse model of DS. We utilized the Dp(17)3Yey/ + mice, a DS mouse line developed by Yu’s group in 2010 [21, 22]. This mouse contains an additional copy of the entire Mmu17 region syntenic to Hsa21. This region includes the gene for CBS, as well as several other genes, including the ATP-binding cassette, sub-family G (WHITE), member 1 (Abcg1) gene, and ribosomal RNA processing 1 homolog B (Rrp1b) gene [21, 22]. Using the Dp(17)3Yey/ + mice, we have now quantified CBS expression in the DS mouse brain, as well as defined its localization to specific cell types. In addition, we have evaluated the effect of AOAA on neurobehavioral alterations; changes in synaptosomal function; various pathways of cell dysfunction such as endoplasmic reticulum stress (ER stress) and autophagy, as well as H_2_S levels; alterations of total proteins (proteomics) persulfidated proteins (persulfidome analysis); and various small molecule substrates, intermediates, and products of cell metabolism metabolites (metabolomics) in the brain.

Our findings support the upregulation and the functional role of the CBS/H_2_S pathway in the pathogenesis of DS. The findings also reveal unexpected sex differences in these responses.

## Materials and methods

### Animals

The transgenic Dp(17)3Yey/ + (strain: #013531) mouse model of DS [21, 22] was purchased from the Jackson Laboratory (Bar Harbor, USA). Hemizygous mice were bred to the non-carrier, wild type (WT), C57BL/6 J mice (Janvier Labs, France) in the animal unit of Fribourg University. Their offspring were ear-punched and genotyped for the targeted duplication by polymerase chain reaction with sequence-specific primers as described previously [21, 22]. Primers were purchased from Microsynth AG (Switzerland), and their sequences are presented in Table S1. Offspring negative for the targeted mutation served as the wild-type (WT) controls in the present study, while their littermates carrying the duplication formed the DS experimental groups.

Both male and female mice were used in our study, with age and sex balance ensured in all in vivo and ex vivo protocols/methodologies followed. When the treatments started, the animals were 5–6 weeks old; behavioral studies started at weeks 6–7 and the animals were 7–8 weeks old at the completion of the study. The age of the animals was selected to correspond to their adolescent/young adult period, which is appropriate for the subject matter of our project. Mice were housed in individually-ventilated cages, enriched with fine Aspen bedding, standard nesting material, Aspen chew sticks, and a non-toxic polycarbonate play tunnel. Colony room temperature was 22 ± 2 °C. Relative humidity was 50 ± 15%. Animals were under a 12-h light/dark cycle, with the lights coming on at 07:00 AM. Animals had ad libitum access to food and water. Daily handling of the animals for 2 weeks preceded the introduction of the treatments. A day before the treatment initiation, the body weights of the animals were recorded and utilized for animal randomization into the control and experimental treatment groups by ensuring the same mean body weight value per group.

For the main part of the manuscript, control and experimental treatments refer to the administration of saline (0.9% NaCl) or 1 mg/kg of AOAA (CAS No: 2921–14-4, Sigma-Aldrich, Switzerland), respectively. In the supplemental data, results of our studies with two additional higher dose groups of AOAA (3 mg/kg/day and 10 mg/kg/day) are also included. AOAA solutions were freshly prepared from AOAA powder immediately prior to administration. Mice were dosed intraperitoneally at a volume of 0.2 ml once a day for 14 days. During the second week of the study, exploratory behavior, spatial learning, reference memory, and recognition memory were assessed in the paradigms of spontaneous alternation T-maze, open field, and novel object recognition, respectively. Animals were additionally monitored for their body weight every 4 days over the study period. All experimental procedures and animal handling were carried out in the light phase, with at least an hour gap from the light/dark transition, and at the same time period each day. Mice were acclimatized to the corresponding experimental rooms for 30 min prior to the commencement of any experimental procedure and behavioral testing.

Experimental protocols and purposes of the study were approved and licensed by the Food Safety and Veterinary Affairs Department of the Canton of Fribourg (Reference ID: 2019_11_FR and 2022–23-FR). Animal handling and experiments were performed in accordance with the “Swiss Federal Animal Welfare Act of 16 December 2005.” Health state of the animals was assessed daily throughout the study. Treatment-related adverse effects or signs of pain were not observed. No premature euthanasia was necessary. All efforts were made to minimize animal suffering and reduce the number of animals used.

### Spontaneous alternation T-maze paradigm

The spontaneous alternation T-maze task builds on the animal’s natural preference for alternating and exploring novel environments to evaluate spatial learning and reference memory [23, 24]. The task was conducted in a Perspex® acrylic apparatus (Ugo Basile®, Italy). The apparatus consisted of two short goal arms (*L* 35 cm × *W* 15 cm) and a long start arm (*L* 65 cm × *W* 15 cm) forming a T shape. The arms were surrounded by a 15-cm high continuous wall. The task comprised an acquisition trial during which each mouse was first placed in the starting arm for 60 s, and the guillotine door separating the starting arm from the central arm was slowly lifted. The mouse was then allowed to run and choose between the two available goal arms. Upon arm selection, the guillotine door separating the central arm from each goal arm was slowly lowered, and the mouse was confined in its chosen goal arm till the end of the acquisition trial (total cut-off time = 180 s). Followingly, each mouse undertook two successive retention trials with a cut-off time of 90 s and an intertrial interval of 1 h on the same day. Another two probe trials followed 24 h and 25 h later, respectively. Mice had free access to both goal arms in all runs. At the end of each session, each mouse was gently removed from the apparatus and returned to its home cage. The apparatus was then thoroughly cleaned with 70% ethyl alcohol to eliminate residual olfactory cues. All trials were performed under dim light conditions (15 ± 5 lx). The arm selected by each animal in each session was recorded by an experimenter blind to the animal genotype and its assigned treatment. Retention trials were marked as successful if the mouse chose different goal arms from the previous run and failures if the mouse chose the same goal arm as the previous one. The alternation rate was defined as the total proportion of successful trials for each animal per day. An alternation rate exceeding the chance level (≥ 0.5) signified spontaneous spatial alternation and reference memory recall.

### Open field paradigm

Relative anxiety levels, exploratory behavior, and general motor function were assessed in the open field paradigm, as previously described [23, 24]. The testing apparatus consisted of a round, grey-colored, Plexiglass arena with a 60-cm perimeter, surrounded by a 30-cm high continuous wall. Light intensity of 15 ± 5 lx was reaching the apparatus. Each mouse was gently placed into the center of the arena and allowed to freely explore the apparatus for 10 min, with the experimenter out of the animal’s sight. The activity of the animal in the arena was video-recorded with the ANY-maze Megapixel USB 2 camera equipped with a lens of up to 12 mm focal length (Stoelting Co., Ireland) and mounted above the center of the arena. Footages were subsequently processed with the automated tracking/analyzer software ANY-maze (RRID:SCR_014289; Stoelting). Within the software, the testing arena was divided into three zones—the center, middle, and outer zone, with a diameter of 10 cm each. Each generated track represented the total distance covered over the testing session in the open field arena and was analyzed for the mean velocity along with the time spent and path traveled in the center. Vertical activity (also known as rearing that described the events when the mouse raised its forepaws, stood on its hind paws, and extended its head upwards) along with the grooming events (referring to the events when the mouse licked/scratched its fur, washed its face, and/or licked its genitalia) were manually recorded during the testing session. At the end of each trial, any fecal deposits were counted and removed, and the apparatus was thoroughly cleaned with 70% ethyl alcohol to eliminate any residual olfactory cues. All measurements and data processing were performed by an experimenter blind to the genotype and treatment of the animal.

### Novel object recognition paradigm

The novel object recognition paradigm builds on the animal’s natural preference for novelty and evaluates hippocampal-dependent recognition memory, a form of declarative memory [23, 24]. It comprised two phases—the acquisition and the testing phase. The acquisition of the behavioral task commenced 48 h following the open field task, during which the mouse was placed in the center of the familiar arena that at the time contained two identical objects placed along a straight line 10 cm away from the nearest wall. Each mouse was allowed to explore the enriched environment for 10 min. After a retention interval of 3 h, each mouse was assigned to re-explore the familiar arena in the presence of an object identical to the previously encountered one and a novel object to evaluate its intermediate-term recognition memory over a 5-min trial. The locations of a novel and familiar object were counterbalanced within each animal group to prevent bias for a particular area of the apparatus. Similarly, the type of object served as a novel was counterbalanced within each animal group, although C57BL/6 and Dp(17)3Yey/ + mice had displayed equal preference for the red plastic cube and green wooden triangle block during pilot studies in our laboratory. Over both trials, light and noise conditions remained identical to the ones during the open field task. We recorded the activity of each animal over all phases of the task using the overhead ANY-maze Megapixel USB 2 camera. At the end of each session, the apparatus and the objects were thoroughly cleaned with 70% ethyl alcohol to eliminate any residual olfactory cues. Footages were subsequently processed with the automated tracking/analyzer software ANY-maze (RRID:SCR_014289) to quantify the exploration time allotted in each object during acquisition and retention trials. A mouse was considered to explore an object when its nose was directed toward the object at a distance ≤ 2 cm. Data were then processed for the calculation of the Recognition Index (RI) that describes the percent ratio of the time spent in the object in the familiar or novel location over the total time spent in object exploration during the retention trial. RI for the novel location exceeding the chance level (50%) signified task acquisition, novelty preference, and recognition memory retrieval. We also recorded and compared the total exploration time allotted to the objects during the acquisition trial, along with the velocity and total path length of each animal in the retention trial to ascertain that no lack of motivation or emotional defects had interfered with the task acquisition and memory testing. Processing of all video recordings was carried out by an experimenter blind to the inherited genotype and assigned a treatment group of the animals. Animals that spent ≥ 95% of their time in the outer zone of the apparatus that led to ≤ 5 s allotted in the object exploration and obscured the learning process during the acquisition trial were excluded from the analysis, according to published guidelines [24]. One transgenic Dp(17)3Yey/ + mouse in the saline group displayed a clear preference for the left location of the object during the task acquisition, having allotted more than 75% of its exploration to it; it was excluded from the analysis to prevent any possible bias of the probe trial findings and the subsequent calculations.

### Collection and processing of animal samples

Following the end of the behavioral assessment, animals were deeply anesthetized and transcardially perfused with a heparinized saline solution. Prior to perfusion, cardiac blood was collected and assayed with a VetScan® VS2 analyzer (Abaxis Europe GmbH, Germany) as described [14]. Brain tissue was then rapidly removed from the skull of each animal and divided into the two cerebral hemispheres. Right and left hemispheres were subsequently processed for synaptosome preparation and tissue homogenization for whole-cell protein extraction, respectively. Alternatively, collected hemispheres were used for metabolomic analysis. Five animals per group were used for histological and immunohistochemical analysis (3 males/2 females per group). These animals were perfused using 0.9% NaCl, followed by perfusion with 4% PFA in 0.9% NaCl. Brains were removed, post-fixed for 24 h in 4% PFA in TBS, and cryopreserved in 30% sucrose-TBS 0.1 M, pH 7.3 at 4 °C.

### Synaptosome preparation and processing

Immediately upon tissue collection, the hindbrain was removed, and the rest of the brain tissue (i.e., forebrain and midbrain) was homogenized by mechanical shearing with a Wheaton™ Dounce tissue grinder in pre-cooled Syn-PER® Synaptic Protein Extraction Reagent (ThermoFisher Scientific, Switzerland) supplemented with protease/phosphatase inhibitor cocktail (1X) on ice. Followingly, the homogenates were sequentially centrifuged at 1000 × *g* for 12 min and 11,500 × *g* for 30 min at 4 °C. The fractions of the cytosol and synaptosomes were collected and assayed for their protein content with Pierce™ Coomassie Plus (Bradford) Assay (ThermoFisher Scientific). The purity of the preparations along with the subcellular localization of rate-limiting enzymes in the H_2_S anabolism and the synaptic expression levels of the oxidative phosphorylation (OXPHOS) protein complexes were determined by immunoblotting. Synaptosomes were further assayed for (a) complex IV enzymatic activity (Abcam, UK), (b) Seahorse XF Real-Time ATP production rate analysis (Bucher Biotech AG, Switzerland), and (c) local vesicle turnover under stimulation.

### Complex IV enzymatic activity assay

After isolation, 100 µg of synaptosomes from each experimental sample was assayed for the synaptic complex IV enzymatic activity using the rodent microplate assay kit from Abcam and following the manufacturer’s protocol. The assay was formatted in the kit-provided 96-well ELISA microplate. Experimental samples along with the assay buffer control were loaded in a total volume of 200 µl of the kit-provided assay buffer per well and incubated for 3 h at room temperature with a gentle agitation in the dark. During this step, the complex IV enzyme present in each sample was immunocaptured by the pre-bound monoclonal antibody within the wells. The enzyme activity was colorimetrically monitored by following the oxidation of reduced cytochrome c by the absorbance change at 550 nm over 2 h with an inter-measurement interval of 1 min using the Infinite® 200 PRO microplate reader (Tecan, Switzerland). The temperature of the instrument was pre-set and maintained at 30 °C during the measurements.

### Seahorse XF extracellular flux analysis

Extracellular flux analysis [15] was used for real-time quantification of the rate of oxygen consumption (OCR) and extracellular acidification (ECAR) owing to adenosine triphosphate (ATP) turnover at the synaptic sites. The assay was formatted in Seahorse XF_e_24 V7 cell culture microplates (Bucher Biotech AG), in which 100 µg of each collected synaptosome fraction was loaded per well in duplicate and attached to the bottom of the well by centrifugation at 3600 × *g* at 4 °C for 1 h. Followingly, the Syn-PER™ reagent was replaced with 500 µl of pre-warmed, pH-calibrated (pH 7.4) assay buffer. The latter consisted of 3.5 mM KCl, 120 mM NaCl, 1.3 mM CaCl_2_, 0.4 mM KH_2_PO_4_, 1.2 mM Na_2_SO_4_, 2 mM MgSO_4_, 15 mM D-glucose, 4 mg/ml fatty acid-free bovine serum albumin (BSA), and 10 mM sodium pyruvate. The plate was incubated for 20 min at 37 °C under non-CO_2_ conditions and then loaded into the Seahorse XF_e_24 analyzer according to the manufacturer’s instructions. A protocol of sequential inhibition of the mitochondrial respiratory system was implemented to measure indices of intracellular ATP production. Oligomycin A and rotenone/antimycin A were respectively injected to the final working concentration of 5 µM and 1 µM through the ports B and C of the Seahorse Flux Pack cartridges (Bucher Biotech AG) to serially inhibit the ATP synthase (complex V) and the electron flux through complexes III and I that would eventually shut down the mitochondrial respiration. ATP synthase inhibition allows the quantification of the rate of basal respiration utilized for ATP production, particularly for mitochondrial ATP production. The inhibition of the electron flux through complexes III and I accounts for mitochondrial-related acidification, further allowing the determination of the anaerobic, glycolytic ATP production rate. All measurements and calculations were performed with Seahorse Wave (RRID:SCR_014526) software (Agilent Technologies).

### Synaptic vesicle turnover assay

Synaptic vesicle turnover was monitored using a stimulation protocol previously described [25, 26]. The assay was formatted in the Corning® 96-well black polystyrene microplate (Sigma-Aldrich), in which isolated synaptosomes from each experimental sample (150 µg in 50 µl Syn-PER® reagent) were incubated in 150 µl of pre-warmed Krebs–Henseleit buffer modified (Sigma-Aldrich) supplemented with 1.3 mM CaCl_2_ at 37 °C with gentle agitation for 10 min. The invaginating synaptic membrane was loaded with 100 µM FM2-10 (ThermoFisher Scientific), which is a fluorescent styryl dye with λ_excitation_ = 506 nm/ λ_emission_ = 620 nm from the FM family introduced by Betz and colleagues to image vesicle recycling dynamics [27]. The dye uptake was stimulated with 33 mM KCl at 37 °C with gentle agitation for 10 min. Followingly, synaptosomes were washed thrice with short 60 s spins to remove the excess (externally bound) and resuspended in a pre-warmed incubation medium. After a 10-min rest period, the invaginated dye was unloaded from the nerve terminals with a second KCl challenge. Dye unloading was monitored in a kinetic mode for 600 s with an inter-reading interval time of 1 s. The fluorescent signal during the resting phase was monitored twice with endpoint measurements. An Infinite® 200 PRO microplate reader was employed for fluorescent signal monitoring, and the temperature of the instrument was pre-set and maintained at 37 °C. Negative control, which describes a sample without tissue handled identically to the rest experimental samples, was included to determine the assay background. The latter was then subsequently subtracted from the raw fluorescent readings for data normalization. Samples were run in duplicate.

### Western blotting

Immunoblotting of whole-cell tissue lysates and isolated subcellular fractionations was performed following our previously published methodology [15]. Membranes were blocked in 5% skimmed milk and probed with primary antibodies (sources and dilutions as follows): CBS (Cell Signaling, #14,782; 1:1,000), 3-MST (Abcam, ab154514; 1:500), CSE (Abcam, ab151769; 1:1,000), ETHE1(Abcam, ab174302; 1:1000), TST (Abcam, ab166625; 1:1,000), CARS2 (Sigma-Aldrich, 1:1,000), SQR (Sigma-Aldrich, 1:1,000), SUOX (Invitrogen, 1:1,000), SOD1 (E4G1H, Invitrogen, 1:1,000), post-synaptic density protein (PSD95) (Cell Signaling, #3450; 1:1,000), synaptophysin (Sys) (Cell Signaling, #5461; 1:1,000), protein kinase–like endoplasmic reticulum kinase (PERK) (Cell Signaling, #5483; 1:1,000), phospho-PERK (Thr 980) (ThermoFisher Scientific, MA5-15,033; 1:1,000), ERO1-like protein α (ERO1-Lα) (Cell Signaling, #3264; 1:1,000), autophagy-related 6 (ATF6) (Abcam, ab227830; 1:1,000), Chop (Cell Signaling, #2895; 1:1,000), phosphorylated inositol-requiring transmembrane kinase/endoribonuclease 1α (phospho-IRE1α) (Ser 724) (Abcam, ab48187; 1:500), immunoglobulin heavy-chain binding protein (BiP) (Cell Signaling, #3177; 1:1,000), protein disulfide isomerase (PDI) (Cell Signaling, #3501; 1:1,000), autophagy-related 7 (Atg7) (Cell Signaling, #8558; 1:1,000), beclin-1 (Cell Signaling, #3495; 1:1,000), autophagy-related 3 (Atg3) (Cell Signaling, #3415; 1:1,000), microtubule-associated protein 1A/1B-light chain 3B (LC3B) (Cell Signaling, #2775; 1:1,000), OxPHOS Rodent WB Cocktail (ThermoFisher Scientific, 45–8099; 1:500), and beta-actin (Cell Signaling, #3700 or #4970; 1:10,000).

When analyzing differences between various groups, the densitometric data were always normalized to its corresponding actin loading control. In turn, ratios of the normalized data are presented, with the control (values measured in the vehicle-treated wild-type mice) protein/actin ratios taken as 1 (i.e., 100%).

### Histological and immunohistochemical analysis of the hippocampus

Hematoxylin and Eosin (H&E) staining was used to detect putative morphological changes in the brain isolated from wild-type control and DS mice. FD Rapid MultiStain™ Kit, FD Hematoxylin Solution (Cat. #: PS104), and FD Eosin Y Solution (Cat. #: PS103) were used according to the manufacturer’s instructions. Coronal brain sections were mounted directly on adhesive microscope slides (MicroSlides SuperFrost® Menzel Epredia, ThermoScientific, Huberlab, 10.0120.02) covered with silani gel. Mounted sections were then air-dried at room temperature for 60 min before staining. All microscope slides were then placed in a staining tray and processed at the same time. Slides were soaked in xylene, passed through several changes of ethanol (100–75%), hydrated and stained with FD hematoxylin solution (2 min), and rinsed in tap water and distilled water containing 2% glacial acetic acid, before being stained with FD Eosin Y Solution (5 min), cleared in xylene and coverslip in resinous mounting medium. FD Hematoxylin Solution (Cat. #: PS104) was formulated for the staining of both neuronal and non-neuronal cellular elements; hematoxylin stains nuclear components, including heterochromatin. Nuclei and basophilic cellular elements were stained blue. Using FD Eosin Y Solution (Cat. #: PS103), cytoplasmic components including collagen and elastic fibers, muscle fibers, and red blood cells were stained in various shades of pink. A fully automated slide scanner NanoZoomerS60 C13210-01 (Hamamatsu Photonics K. K, Switzerland) was used for image acquisition. Images were analyzed in NDP.view2 Image viewing software (NanoZoomer, Hamamatsu Photonics K.K, U12388-01).

For immunohistochemical analysis of the hippocampus, free-floating coronal brain sections were incubated in a blocking solution containing 1xTBS, 5% normal goat serum, and 0.3% Triton X-100, for 1 h at room temperature. Sections were then incubated in primary antibody solution containing 1xTBS, 1% BSA, and 0.3% Triton X-100 overnight at 4 °C. For the localization of astrocytes, an antibody against glial fibrillary acidic protein (GFAP) (Cell Signaling, #3670; 1:100) was used. For the localization of CBS, a CBS antibody (Cell Signaling, #14,782; 1:300) was used. On the following day, the sections were washed twice in TBS and once in Tris–HCl, for 5 min each. Secondary antibodies goat anti-mouse IgG (H + L) Highly Cross-Adsorbed Secondary Antibody Alexa Fluor Plus 488 (1:1,000 dilution) and goat anti-rabbit IgG (H + L) Highly Cross-Adsorbed Secondary Antibody Alexa Fluor Plus 568 (1:1,000) were added for 1 h at room temperature. Sections were counterstained with 1 μg/ml DAPI (Merck, MBD0015) for the last 5 min. After the final wash, sections were transferred to microscopy slides (MicroSlides SuperFrost® Plus Menzel Epredia, ThermoScientific, Huberlab, 10.0344.01) and mounted with a drop of ProLong™ Gold Antifade Mountant (ThermoFisher Scientific, P36930).

Mounted brain sections were first scanned by a fully automated slide scanner NanoZoomerS60 C13210-01 (Hamamatsu Photonics K. K, Switzerland) with a × 20 objective (NA 0.75) and scanning resolution of 0.46 μm/pixel and analyzed in NDP.view2 Image viewing software (NanoZoomer, Hamamatsu Photonics K.K, U12388-01). Identical parameters for scanning were applied for parallel-processed sections either in bright-field acquisition or in fluorescent acquisition. Fluorescence images were acquired using the mercury lamp unit using DAPI, FITC, and Cy3 filter cube with excitation filters (387, 485, and 560 nm) and emission filters (410, 504, 582 nm). Specific regions were analyzed using the “free-hand” tool in the NDP.view2 software (Hamamatsu Photonics, Switzerland). The Leica STELLARIS 8 FALCON inverted laser scanning confocal microscope was used for examination of the hippocampal region. A × 40 objective (HC PL APO CS2 × 40/1.10 WATER) was used. A DMOD 405 laser was used for DAPI excitation. A pulsed supercontinuum white light laser was used for the excitation of Alexa 488 and Alexa568. Proprietary Acousto-Optical Beam Splitter (AOBS) enabled the use of simultaneous independent laser lines. Fluorescence of DAPI was acquired by excitation at Ex 405 nm and recording the emission at Em 415–470 nm; Alexa FluorTM 488 dye was acquired at Ex 488 nm, Em 498–555 nm; and Alexa FluorTM 568 fluorescence was acquired at Ex 553 nm, Em 570–630 nm. Scanning format used for the confocal acquisition was as follows: 1240 × 1240 pixels, scan speed 200 Hz, Z-step interval 0.42 μm.

For image reconstruction and morphometric analyses, Imaris 9.8.2® software (Bitplane, AG, Switzerland) was used. For automatic neuron tracing and analysis of neuronal branching, the “Filament Tracer” software package was used. For volume and fluorescence intensity determination, the “Cell” software package and the “Surface” software package of the Imaris software were used. The same parameters and algorithm settings were applied for comparison between the various experimental groups.

### Measurement of CBS and 3-MST activity in brain homogenates

Brain homogenates from wild-type and DS mice were also evaluated for their H_2_S-generating ability ex vivo using the 7-azido-4-methylcoumarin (AzMC) dye, as described [14]. Brains were rapidly removed from the skull and dissected into coronal sections of 1 mm. Two-to-three sections per mouse were placed into a well of a 24-well tissue plate that contained 1 ml of pre-warmed artificial cerebrospinal fluid (aCSF; 120 mM NaCl, 3.5 mM KCl, 1.5 mM CaCl_2_, 0.4 mM KH_2_PO_4_, 1 mM MgCl_2_, HEPES 5 mM) that had been previously supplemented with 4.5 g/l D-glucose, 0.23 mM sodium pyruvate, and 4 mg/ml fatty acid-free BSA and adjusted to a pH of 7.4. At the end of treatment incubation, brain tissue was homogenized by mechanical shearing (∼ 20 strokes) with a Wheaton™ Dounce tissue grinder in pre-cooled radioimmunoprecipitation assay buffer (RIPA buffer) on ice; RIPA buffer was supplemented with 1X Halt™ protease/phosphatase inhibitor cocktail (Thermo Fisher Scientific, Basel, Switzerland). Protein was collected by centrifugation at 10,000 × *g* for 10 min at 4 °C. Protein concentration was determined with the BCA assay (Pierce™ BCA Protein Assay Kit by Thermo Fisher Scientific).

Protein samples of each experimental condition were then assayed for CBS enzyme activity (H_2_S generation) and carried out in 96-well flat-bottomed black microplates, using the H_2_S-selective fluorescent probe AzMC, an Infinite 200 Pro plate reader (Tecan, Männedorf, Switzerland), in a total assay volume of 200 µl. Each well received 50 mM Tris–HCl pH 8.0, 150 µg protein, 5 µM pyridoxal 5′-phosphate hydrate (PLP; AppliChem GmbH, Darmstadt, Germany), 10 µM AzMC, and ± 500 µM S-adenosyl-L-methionine (SAM; Cas No: 86867–01-8). The reaction mixture was resuspended 10 times, followed by incubation at room temperature for 10 min in the absence of light. The enzymatic activity was then triggered by dispensing a mixture of 500 µM L-homocysteine (Hcy; CAS No: 6027–13-0) and 2 mM L-cysteine (Cys; AppliChem GmbH) followed by 5 times resuspension. The blanks received buffer in place of homogenate under otherwise identical conditions. The increase in the probe fluorescence (λ_excitation_ = 340 nm; λ_emission_ = 460 nm) was monitored over 2 h at 37 °C. CBS activity was determined from the initial slope of the fluorescence increase over time.

For the assessment of 3-MST activity, the conditions were similar, except for SAM and PLP which were omitted instead of L-homocysteine, the reaction was initiated with the 3-MST substrate 3-mercaptopyruvate (500 µM, CAS No: 2464–23-5).

### Quantification of brainH_2_S and polysulfide levels and related small molecules

The modified monobromobimane method was used to measure bioavailable H_2_S concentrations in plasma, as described [28–30]. Twenty-five microliters of plasma was mixed with 66 μl working solution (65 μl 200 mM HEPES pH 8.2 + 1 μl 100 mM monobromobimane in ACN) and kept at 20.0 °C for 10 min before the addition of 10 μl 50% trichloroacetic acid to stop the reaction. Precipitated proteins were removed by centrifugation at 3000 × *g* for 5 min, and 3 μl of the supernatants was injected into the HPLC. The derivatized product (sulfide-dibimane) was separated on a Phenomenex Luna C18(2) 250 × 2 mm 3 μm column using a linear gradient elution with 0.1% TFA/H2O (A) and 0.1% TFA/ACN (B) at a flow rate of 0.25 ml/min: 0 min, 15%B; 3 min, 35%B; 8.5 min, 35%B; 10.5 min, 90%B; 11 min, 90%B; 12 min, 15%B; and 14 min 15%B. Products were detected and quantitated using fluorescence excitation set at 390 nm and detection wavelength at 475 nm.

Cryopulverized tissue samples (approx. 10–30 mg) were suspended and sonicated in ice-cold methanol containing 5 mM HPE-IAM and incubated at 37 °C for 20 min under constant vortexing. The reaction mixture was cooled on ice and centrifuged at 14,000 × *g* 4 °C for 10 min. One hundred microliters of the supernatants was acidified with 5 μl 10% FA and transferred into HPLC autosampler vials in a 1:1 ratio with 0.1% FA/H_2_O. Components were separated on a Phenomenex Kinetex C18 50 × 2 mm 2.6 μm column using gradient elution with 0.1% FA/H_2_O (A) and 0.1% FA/MeOH (B) at a flow rate of 0.3 ml/min: 0 min, 5%B; 15 min, 95%B; 17 min, 5%B; and 20 min, 5%B. The following transitions were monitored in the Thermo Scientific Q-Exactive Focus mass spectrometer using HCD fragmentation: Cys, 299–121 m/z; Cys-SH, 331–121 m/z; GSH, 485–356 m/z; GSSH, 517–388 m/z; H_2_S, 389–252 m/z; and H2S2, 421–121. The precipitated proteins were dissolved in 1% SDS/PBS, briefly sonicated, and centrifuged again at 14,000 × *g* at room temperature for 10 min. Supernatants were used to measure protein content using the BCA assay. The modified monobromobimane method was used to measure tissue H_2_S and polysulfide (H_2_S_2_) levels and plasma H_2_S concentrations, as described. LC–ESI–MS/MS analysis with HPE-IAM was used to determine cysteine (Cys), Cys-SH, reduced glutathione (GSH), and oxidized glutathione (GSSH) levels as described [30].

### Measurement of persulfidated proteins in the brain

Mouse brains were lysed in cold HEN buffer (50 mM HEPES, 1 mM EDTA, 0.1 mM neocuproine, 1% IGEPAL, 2% SDS, pH 7.4) supplemented with 10 mM 4-chloro-7-nitrobenzofurazan (NBF-Cl, Sigma, 163,260) and 1% protease inhibitor cocktail (Sigma, P8340). One 5-mm stainless steel bead (Qiagen, 69,989) was added to a tube containing a sample with lysis buffer, and homogenization was performed using TissueLyser II (3 × 1 min at 30 Hz, with a 1-min break on ice in between). Lysates were shortly spun down to collect the liquid and incubated for 2 h at 37 °C. Next, samples were again shortly spun down to remove beads, transferred to 1.5-ml tubes, and centrifuged for 10 min at 18 000 × *g* to remove any insoluble parts. Proteins were then precipitated twice by methanol/chloroform precipitation. Pellets were washed twice with ice-cold methanol. Persulfidation of proteins was assessed as described [31]. To reduce unspecific enrichment, samples were cleaned from endogenously biotinylated proteins. Pellets were dissolved in PBS (Sigma, D8537) supplemented with 0.2% SDS, and protein concentration was performed using DC protein assay (Bio-Rad, 5,000,112). The same amount of protein was incubated with Pierce™ NeutrAvidin™ agarose beads (ThermoFisher, 29,200). After 2 h of mixing at room temperature (RT), supernatants were collected and precipitated using methanol/chloroform precipitation. Pellets were suspended in PBS + 2% SDS and incubated for 1.5 h at 37 °C with 250 µM DCP-Bio1 (MERK, NS1226). Proteins were precipitated twice and washed using the same method as previously described to remove the excess of DCP-Bio1. After precipitation, pellets were resuspended in PBS + 0.1% SDS, and the same amount of protein was incubated with Pierce™ High-Capacity Streptavidin Agarose beads (ThermoFisher, 20,357) to enrich persulfidated proteins. After 4 h of mixing at RT, beads were collected, transferred to Pierce™ Disposable Columns 2 ml (ThermoFisher, 29,920), and washed with 28 ml of PBS and 8 ml of water to remove unbound proteins and detergent. After washes, enriched proteins were eluted by incubating beads overnight with 2.25 M NH_4_OH solution (Merck, 533,003). After incubation, eluates were collected and lyophilized. Lyophilized samples were resuspended in a digestion buffer (50 mM NH_4_HCO_3_, 1 mM CaCl_2_). Samples were digested for 16 h using trypsin (Promega, V5117) at an enzyme-to-protein ratio of 1:20. Samples were then desalted using Supel™-Select HLB SPE Tube (Sigma, 54,181-U) following the manufacturer’s protocol. Shortly, columns were conditioned with the elution solution—60% acetonitrile, 0.1% trifluoroacetic acid (TFA)—and washed twice with washing solution (0.1% TFA).

Each sample was then loaded on the column using gravity flow. Cartridges were washed once with washing solution followed by two-step elution using the elution buffer. Eluted peptides were evaporated to dryness. Peptides were dissolved in 0.1% TFA before being analyzed by high-resolution LC–MS/MS using an Ultimate 3000 Nano Ultra High-Pressure Chromatography (UPLC) system (Thermo Fisher Scientific) coupled with an Orbitrap Eclipse™ Tribrid™ Mass Spectrometer via an EASY-spray (Thermo Fisher Scientific). Peptide separation was carried out with an Acclaim™ PepMap™ 100 C18 column (Thermo Fisher Scientific) using a 180 min gradient: 0 min, 3%B; 110 min, 26%B; and 125 min, 35%B (solvent B corresponds to 84% acetonitrile, 0.1% formic acid) at a flow rate of 250 nL/min. The Orbitrap Eclipse™ was operated in a DDA mode, and MS1 survey scans were acquired from 300 to 1500 m/z at a resolution of 120,000 using the Orbitrap mode. The most intense ions were isolated for 3 s with a 1.2 m/z window and then fragmented by high-energy collision-induced dissociation with a normalized collision energy of 32%, considering a dynamic exclusion of 30 s. MS/MS spectra were recorded using normal-speed IonTrap mode. Data evaluation was performed with PEAKS ONLINE software using 15 ppm for precursor mass tolerance, 0.5 Da for fragment mass tolerance, specific tryptic digest, and a maximum of 3 missed cleavages. NBF (+ 163.00961594 Da) on C, K, and R and DCP (+ 168.0786442 Da) on C, acetylation on N-terminus and oxidation on M were added as variable modification, peptides, and proteins were filtered at FDR 1%. Data filtering for cysteine-containing proteins was performed using the script in Python. Normalization using the EigenMS package and most of the data analysis and visualization were performed in R version 4. 0. 3 employing Rstudio version 1. 3. 1093.

### Untargeted metabolomic analysis of brain samples

Metabolomic analysis of 24 whole-brain samples (6 wild-type controls treated with vehicle, 6 wild-type controls treated with AOAA, 6 DS animals treated with vehicle and 6 DS animals treated with AOAA; in each group, 3 male and 3 female animals) was conducted by Metabolon Inc. (Morrisville, NC USA) as described [32–35]. Samples were prepared using the automated MicroLab STAR® system from Hamilton Company. Several recovery standards were added prior to the first step in the extraction process for QC purposes. To remove protein, dissociate small molecules bound to protein or trapped in the precipitated protein matrix, and recover chemically diverse metabolites, proteins were precipitated with methanol under vigorous shaking for 2 min (Glen Mills GenoGrinder 2000) followed by centrifugation. The resulting extract was divided into five fractions: two for analysis by two separate reverse phase (RP)/UPLC-MS/MS methods with positive ion mode electrospray ionization (ESI), one for analysis by RP/UPLC-MS/MS with negative ion mode ESI, one for analysis by HILIC/UPLC-MS/MS with negative ion mode ESI, and one sample reserved for backup. Samples were placed briefly on a TurboVap® (Zymark) to remove the organic solvent. The sample extracts were stored overnight under nitrogen before preparation for analysis.

Ultrahigh-performance liquid chromatography-tandem mass spectroscopy (UPLC-MS/MS) was performed using the Waters ACQUITY ultra-performance liquid chromatography (UPLC) and a Thermo Scientific Q-Exactive high-resolution/accurate mass spectrometer interfaced with a heated electrospray ionization (HESI-II) source and Orbitrap mass analyzer operated at 35,000 mass resolution. The sample extract was dried and then reconstituted in solvents compatible with each of the four methods. Each reconstitution solvent contained a series of standards at fixed concentrations to ensure injection and chromatographic consistency. One aliquot was analyzed using acidic positive ion conditions, chromatographically optimized for more hydrophilic compounds. In this method, the extract was gradient-eluted from a C18 column (Waters UPLC BEH C18—2.1 × 100 mm, 1.7 µm) using water and methanol, containing 0.05% perfluoropentanoic acid (PFPA) and 0.1% formic acid (FA). Another aliquot was also analyzed using acidic positive ion conditions; however, it was chromatographically optimized for more hydrophobic compounds. In this method, the extract was gradient-eluted from the same afore mentioned C18 column using methanol, acetonitrile, water, 0.05% PFPA, and 0.01% FA and was operated at an overall higher organic content. Another aliquot was analyzed using basic negative ion optimized conditions using a separate dedicated C18 column. The basic extracts were gradient-eluted from the column using methanol and water, however, with 6.5 mM NH_4_HCO_3_ at pH 8. The fourth aliquot was analyzed via negative ionization following elution from a HILIC column (Waters UPLC BEH Amide 2.1 × 150 mm, 1.7 µm) using a gradient consisting of water and acetonitrile with 10 mM ammonium formate, pH 10.8. The MS analysis alternated between MS and data-dependent MSn scans using dynamic exclusion. The scan range varied slighted between methods but covered 70–1000 m/z.

The bioinformatics system consisted of four major components: the laboratory information management system (LIMS), the data extraction and peak-identification software, data processing tools for QC and compound identification, and a collection of information interpretation and visualization tools for use by data analysts. The hardware and software foundations for these informatics components were the LAN backbone, and a database server running Oracle 10.2.0.1 Enterprise Edition. Raw data were extracted, peak-identified, and QC-processed using Metabolon’s hardware and software. These systems are built on a web-service platform utilizing Microsoft’s.NET technologies, which run on high-performance application servers and fiber-channel storage arrays in clusters to provide active failover and load-balancing. Compounds were identified by comparison to library entries of purified standards or recurrent unknown entities. Metabolon maintains a library based on authenticated standards that contain the Retention Time/Index (RI), the mass-to-charge ratio (m/z), and chromatographic data (including MS/MS spectral data) on all molecules present in the library. Furthermore, biochemical identifications are based on three criteria: Retention Index within a narrow RI window of the proposed identification, accurate mass match to the library + / − 10 ppm, and the MS/MS forward and reverse scores between the experimental data and authentic standards. The MS/MS scores are based on a comparison of the ions present in the experimental spectrum to the ions present in the library spectrum. While there may be similarities between these molecules based on one of these factors, the use of all three data points can be utilized to distinguish and differentiate biochemicals. More than 3300 commercially available purified standard compounds have been acquired and registered into LIMS for analysis on all platforms for determination of their analytical characteristics. Additional mass spectral entries have been created for structurally unnamed biochemicals, which have been identified by virtue of their recurrent nature (both chromatographic and mass spectral). These compounds have the potential to be identified by future acquisition of a matching purified standard or by classical structural analysis. Various curation procedures were carried out to ensure accurate and consistent identification of true chemical entities and to remove those representing system artifacts, mis-assignments, and background noise. Library matches for each compound were checked for each sample and corrected if necessary. Peaks were quantified using area-under-the-curve. For studies spanning multiple days, a data normalization step was performed to correct variation resulting from instrument inter-day tuning differences. Each compound was corrected in run-day blocks by registering the medians to equal one (1.00) and normalizing each data point proportionately (“block correction”).

### Statistics and reproducibility

Data are presented as representative blots or the mean values ± standard error of the mean (SEM) of *n* experiments with *n* indicating the number of animals per group. ANOVA followed by Bonferroni’s multiple comparisons test and one-way ANOVA and Dunnett’s multiple comparisons test were used to analyze the numerical data. Statistical methods used for the metabolomic analysis included Welch’s two-sample *t*-test and two-way ANOVA. Significance was indicated as follows: * for *p* ≤ 0.05 and ** for *p* ≤ 0.01. The current study was preceded by a power analysis that was done based on expected differences and AOAA treatment effects based on a prior study in DS rats [14]. Each animal group contained an equal number of male and female animals and was planned for analysis together as a single group. The study was not originally powered for analysis of sex differences. However, because the proteomic findings indicated a marked sex difference, post hoc analysis was also performed for separate male and female subgroups.

## Results

### Expression analysis of H_2_S-generating and H_2_S-metabolizing enzymes in mouse DS brains

**Fig. 1:**
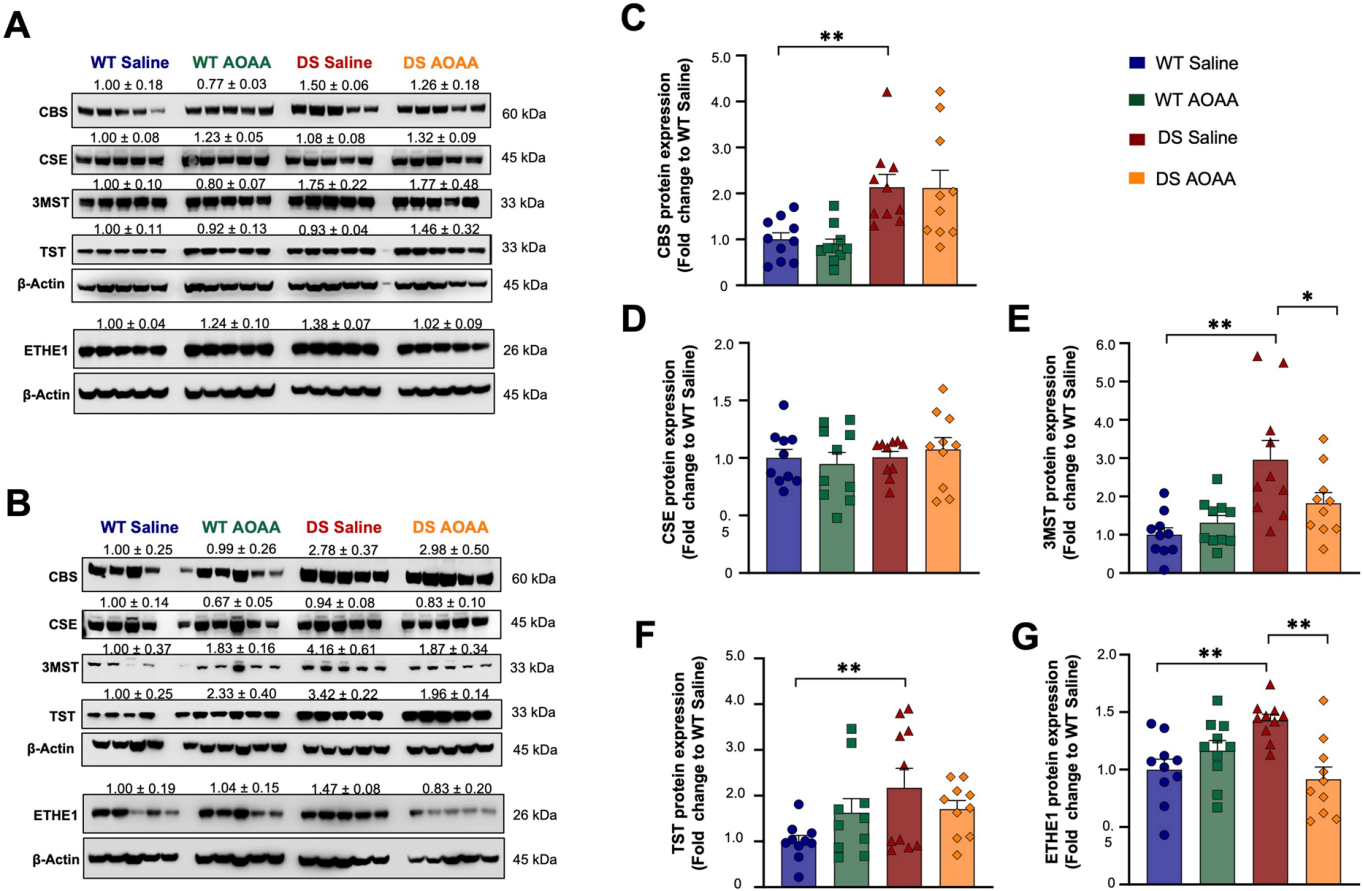
Comparison of the expression of the H_2_S-producing enzymes CBS, CSE, and 3-MST and the H_2_S-metabolizing enzymes TST and ETHE1 in wild-type vs. Dp(17)3Yey/ + mouse brains. **A**, **B** Representative immunoblots of the expression levels of the enzymes for H_2_S metabolism in whole-brain homogenates in **A** male and **B** female animals. **C–G** Collective densitometric analysis for the H_2_S-producing enzymes **C** CBS, **D** 3-MST, and **E** CSE, as well as for the H_2_S-catabolizing enzymes **F** TST and **G** ETHE1 are shown. The housekeeping protein β-actin served as the loading control. Each bar represents the mean ± SEM of 10 animals per experimental group, 5 males and 5 females, with each dot representing one animal; *p ≤ 0.05 and **p ≤ 0.01

**Fig. 2:**
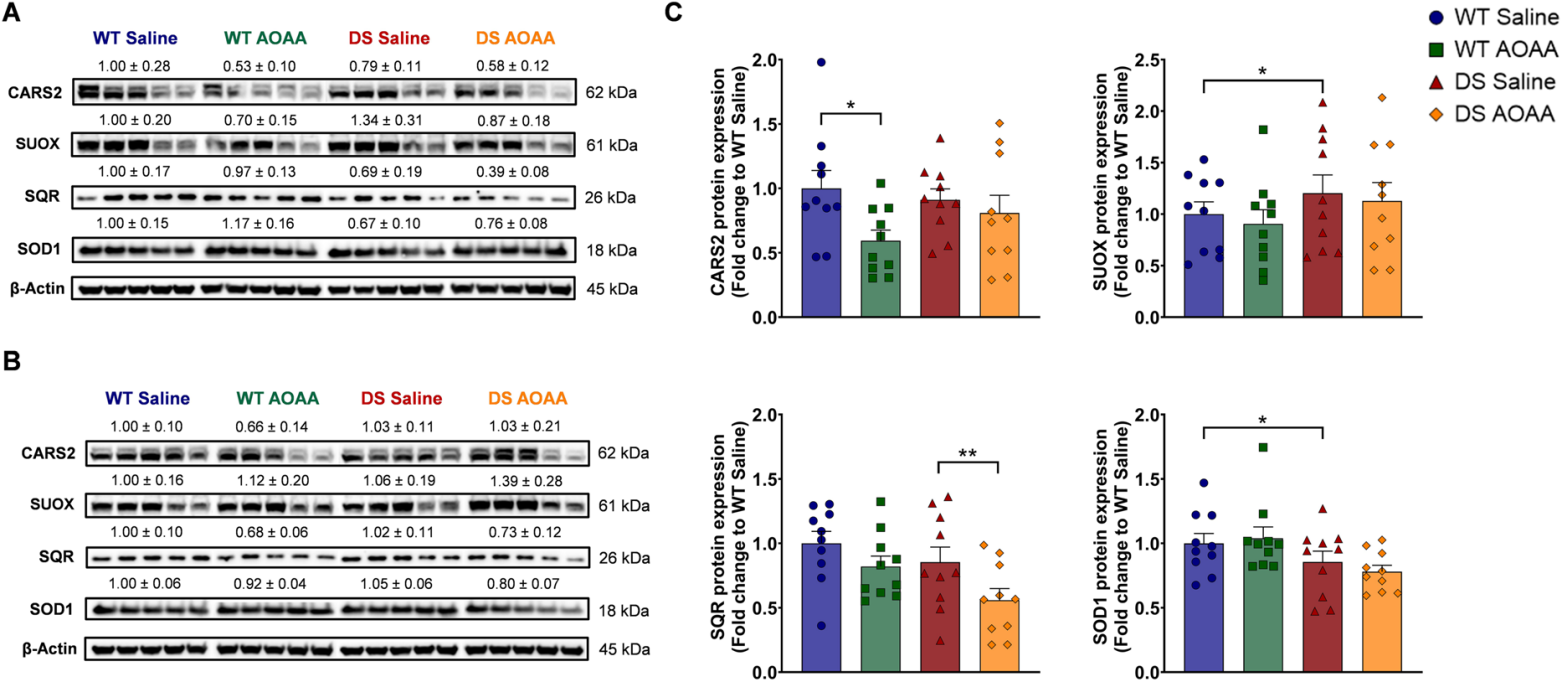
Comparison of the expression of the H_2_S-producing enzyme CARS2 and the H_2_S-metabolizing enzymes SUOX, SQR, and SOD1 in wild-type vs. Dp(17)3Yey/ + mouse brains. **A**, **B** Representative immunoblots of the expression levels of the enzymes for H_2_S metabolism in whole-brain homogenates in **A** male and **B** female animals. **C** Collective densitometric analysis. The housekeeping protein β-actin served as the loading control. Each bar represents the mean ± SEM of 10 animals per experimental group, 5 males and 5 females, with each dot representing one animal; *p ≤ 0.05

Western blotting studies from whole-brain homogenates demonstrated that DS brains contain higher levels of CBS (Fig. 1A–C). Levels of CSE were comparable in wild-type and DS brains (Fig. 1A, B, D). Expression of 3-MST was higher in DS brains than in controls (Fig. 1A, B, E). CARS2 showed comparable expression in wild-type and DS brains (Fig. 2).

Expression of two enzymes involved in H_2_S catabolism, TST (Fig. 1A, B, F), and ETHE1 (Fig. 1A, B, G) was higher in DS than in wild-type brains. Expression of the further H_2_S-decomposing enzymes SQR was comparable in DS and wild-type control brains, and expression of the H_2_S-decomposing enzyme SUOX was lower in DS brains than in wild-type brains and so was the expression of superoxide dismutase 1 (SOD1), which acts as an antioxidant enzyme, but has also been implicated in H_2_S decomposition [36] (Fig. 2). Treatment of the DS mice with AOAA did not significantly affect the expression of CBS, CSE, or TST (Fig. 1C, D, F) but reduced the expression of 3-MST and ETHE1 (Fig. 1E, G). These findings are in line with prior studies demonstrating the upregulation of CBS and 3-MST in DS [14, 37].

**Fig. 3:**
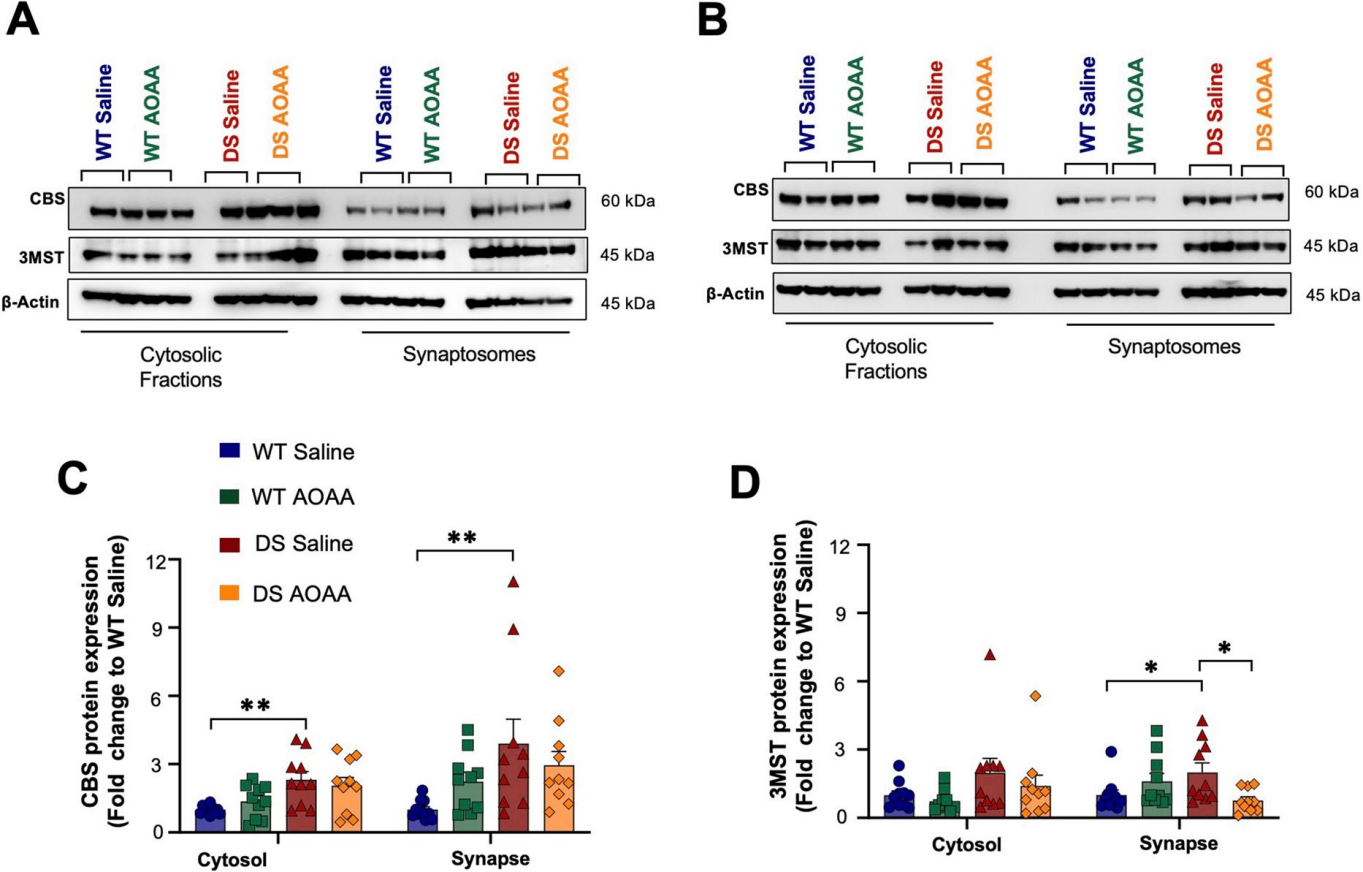
Synaptosomes isolated from Dp(17)3Yey/ + mouse brain exhibit upregulated expression of the H_2_S-producing enzymes CBS and 3-MST compared to wild-type controls. Immunoblots of the expression levels of the enzymes for H_2_S metabolism in cytosolic fractions and synaptosomes from **A** male and **B** female mice, along with the collective densitometric analyses for the H_2_S-producing enzymes **C** CBS and **D** 3-MST. Cytosolic and synaptic expressions are presented. The housekeeping protein β-actin served as the loading control in all densitometric analyses. Each bar represents the mean ± SEM of 10 animals per group, 5 males and 5 females; each dot represents one animal; *p ≤ 0.05 and **p ≤ 0.01

We also assessed the expression of CBS and 3-MST in synaptosomal preparations obtained from mouse brains. Once again, CBS expression was higher in DS synaptosomes than in wild-type control brain synaptosomes, and this was the case both in the synapse and in the cytosol (Fig. 3A–C). 3-MST expression was also higher in DS than in the wild-type synaptosomes, with statistically significant differences for the synapse and a trend for the cytosol (Fig. 3A, B, D). Treatment of the mice with AOAA did not have a significant effect on synaptosomal CBS expression (Fig. 3A–C) but tended to reduce synaptosomal 3-MST expression (Fig. 3A, B, D).

### Morphological analysis of mouse DS brains

**Fig. 4:**
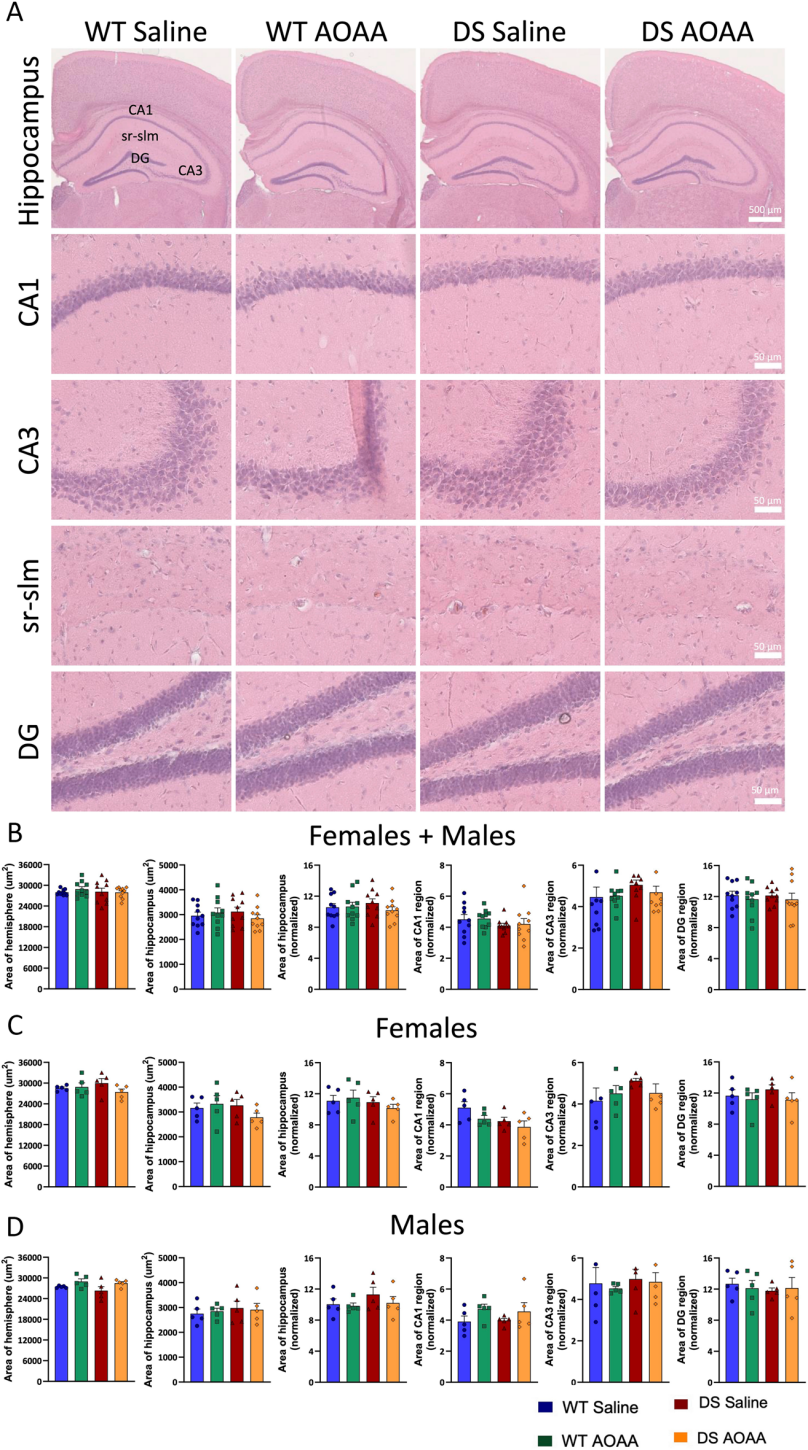
Histological analysis of the architecture of wild-type vs. Dp(17)3Yey/ + mouse brains. Hematoxylin–eosin staining was performed to define the cytoarchitecture of the hippocampus. Hematoxylin stains nuclear components, including heterochromatin and nucleoli. Eosin stains cytoplasmic components: collagen, elastic fibers, muscle fibers, red blood cells. **A** representative images; **B–G** analysis of various brain regions. Each bar represents the mean ± SEM of 5 animals per experimental group; each dot represents one animal. No macroscopic differences were found between the cytoarchitecture of the brains analyzed in the four experimental groups

Prior studies in DS individuals and in various mouse models of DS have demonstrated various differences in brain development [38, 39]. However, histological analysis of the brains did not reveal any significant morphological differences between wild-type and DS mice (Fig. 4), suggesting that the extra copy of the genes present in the current model—which only represents a fraction of the genes that are relevant for human DS—do not significantly regulate brain development—at least at the resolution level of our analysis.

### Immunohistochemical localization of CBS in the DS brain

**Fig. 5:**
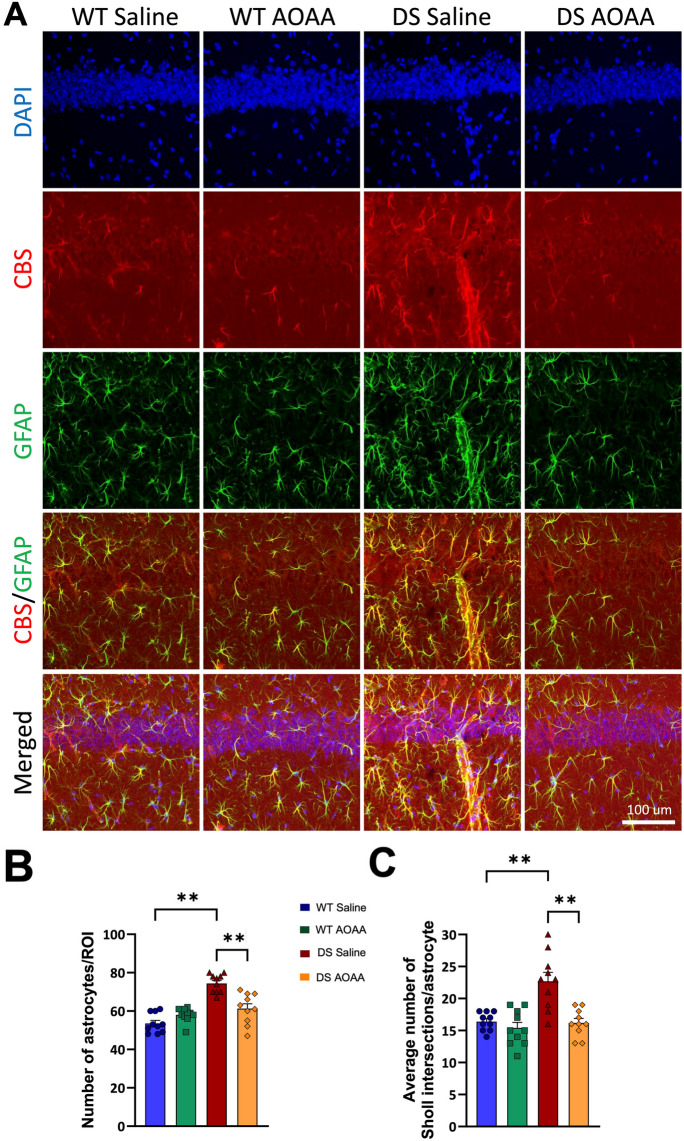
Reactive astrogliosis in the DS brain. Immunohistochemical staining of mouse brain sections of wild-type and DS mice (with or without AOAA treatment) was analyzed in the CA1 region of the hippocampus. Images were acquired using confocal microscopy in 3D at × 40 magnification and are shown as maximum intensity projections of the whole z-stack of 30 μm. Immunofluorescence labeling for marker of astrocytes GFAP (green), CBS (red), and counterstaining with DAPI (blue) is shown. **A** Representative images; **B** astrocyte density; **C** Sholl analysis of astrocyte intersections. Each bar represents the mean ± SEM of 10 animals per group; **p ≤ 0.01. DS was associated with reactive astrogliosis, as evidenced by higher astrocyte numbers, larger astrocyte cell size, and more complex branching

**Fig. 6:**
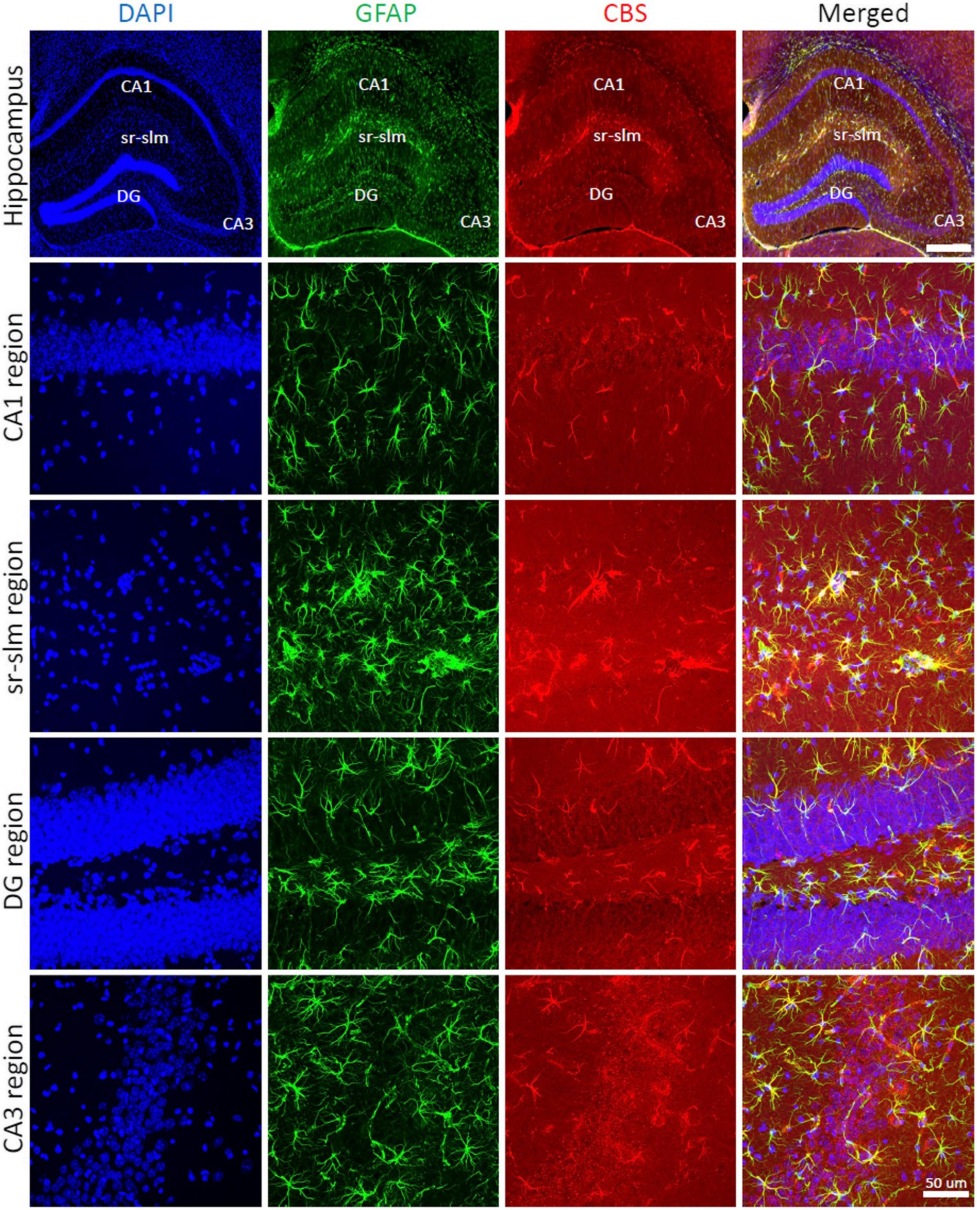
CBS expression is primarily localized in astrocytes in the mouse hippocampus. Immunohistochemical staining of wild-type mouse brain sections was analyzed in 4 different regions of the hippocampus (CA1, sr-slm, DG, CA3). Images in the top row were acquired using NanoZoomer fully automated slide scanner in 2D at × 5 magnification. Images in the other rows were acquired using confocal microscopy in 3D at × 40 magnifications and are shown as maximum intensity projections of the whole z-stack of 30 μm. Immunofluorescence labeling for marker of astrocytes GFAP (green), CBS (red), and counterstaining with DAPI (blue) is shown. Note the colocalization of CBS with GFAP in all regions of the hippocampus

**Fig. 7:**
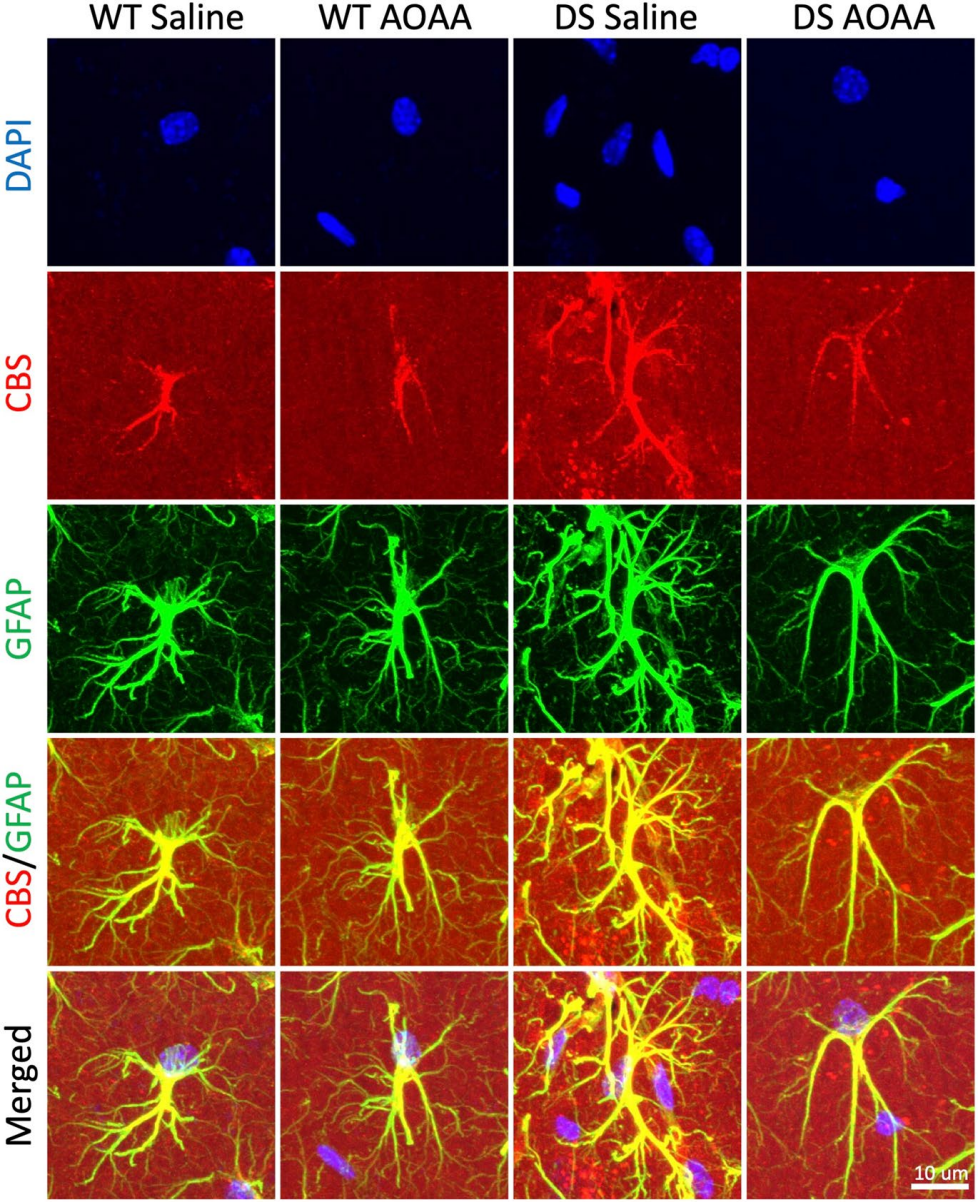
Reactive astrogliosis in DS brains. Representative zoom-in images of astrocytes (GFAP, green) and CBS (red) and merged images with DAPI (blue) are shown. CBS was found mostly in the astrocytes, but it was also present in vascular tissues surrounded by astrocytic end-feet. DS was associated with reactive astrogliosis, as evidenced by higher astrocyte numbers, larger astrocyte cell size, and more complex branching

Immunohistochemical analysis of wild-type mouse brain sections was conducted in 4 different regions of the hippocampus (CA1, sr-slm, DG, and CA3). Figures 5, 6, and 7 show the results of various immunofluorescence labeling experiments and demonstrate that CBS primarily localizes to the astrocytes. Figure 5 shows a comparison of the expression patterns in the 4 groups of mice (wild type, wild type treated with AOAA, and DS treated with AOAA), with representative photomicrographs (Fig. 5A), with quantification of astrocytes/ROI (Fig. 5B) and Sholl analysis, which quantifies astrocyte branch complexity (Fig. 5C). The number of astrocytes was significantly higher in the DS brains than in wild-type control brains, and the complexity of the astrocytes was also higher. These findings are consistent with prior findings in various experimental models of DS, as well as clinical observations of DS, demonstrating higher astrocyte numbers than what can be found in control brains, reflecting a compensatory phenomenon that is often referred to as “reactive astrogliosis” [40–42]. Treatment of the DS mice with AOAA suppressed astrocytosis and reduced astrocyte complexity (Fig. 5B, C).

### Neurobehavioral analysis of DS mice: effect of AOAA

**Fig. 8:**
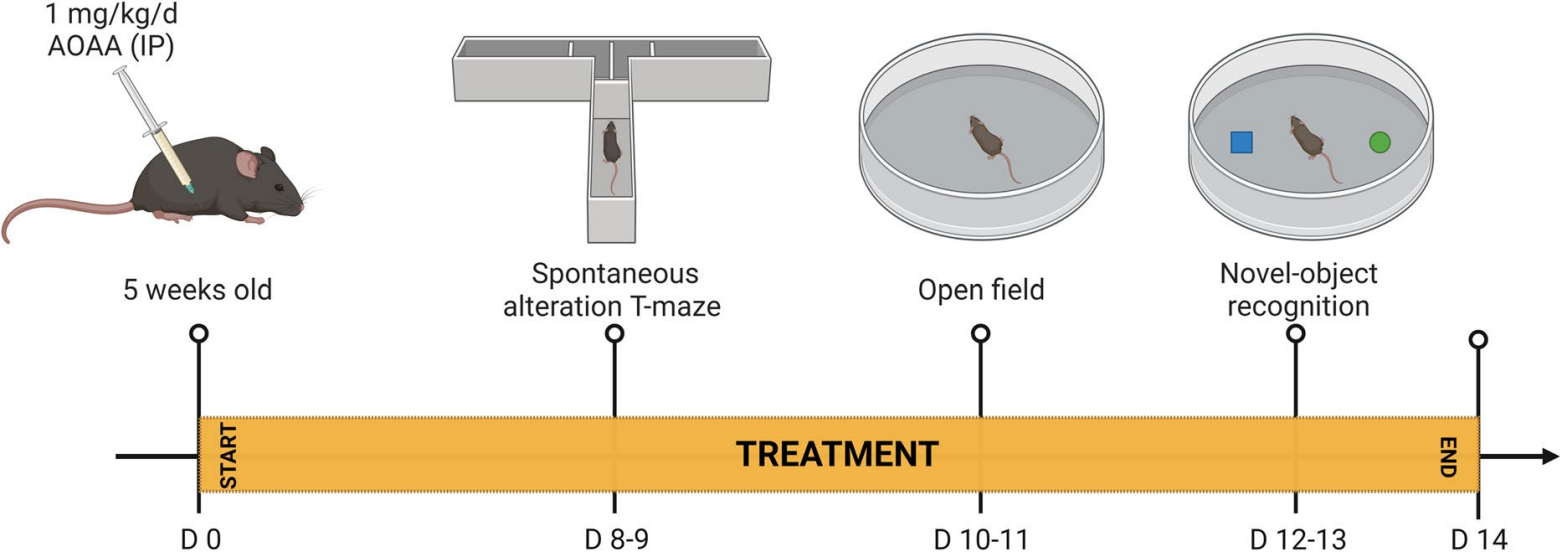
Timeline of the in vivo studies. The letter “D” stands for day

**Fig. 9:**
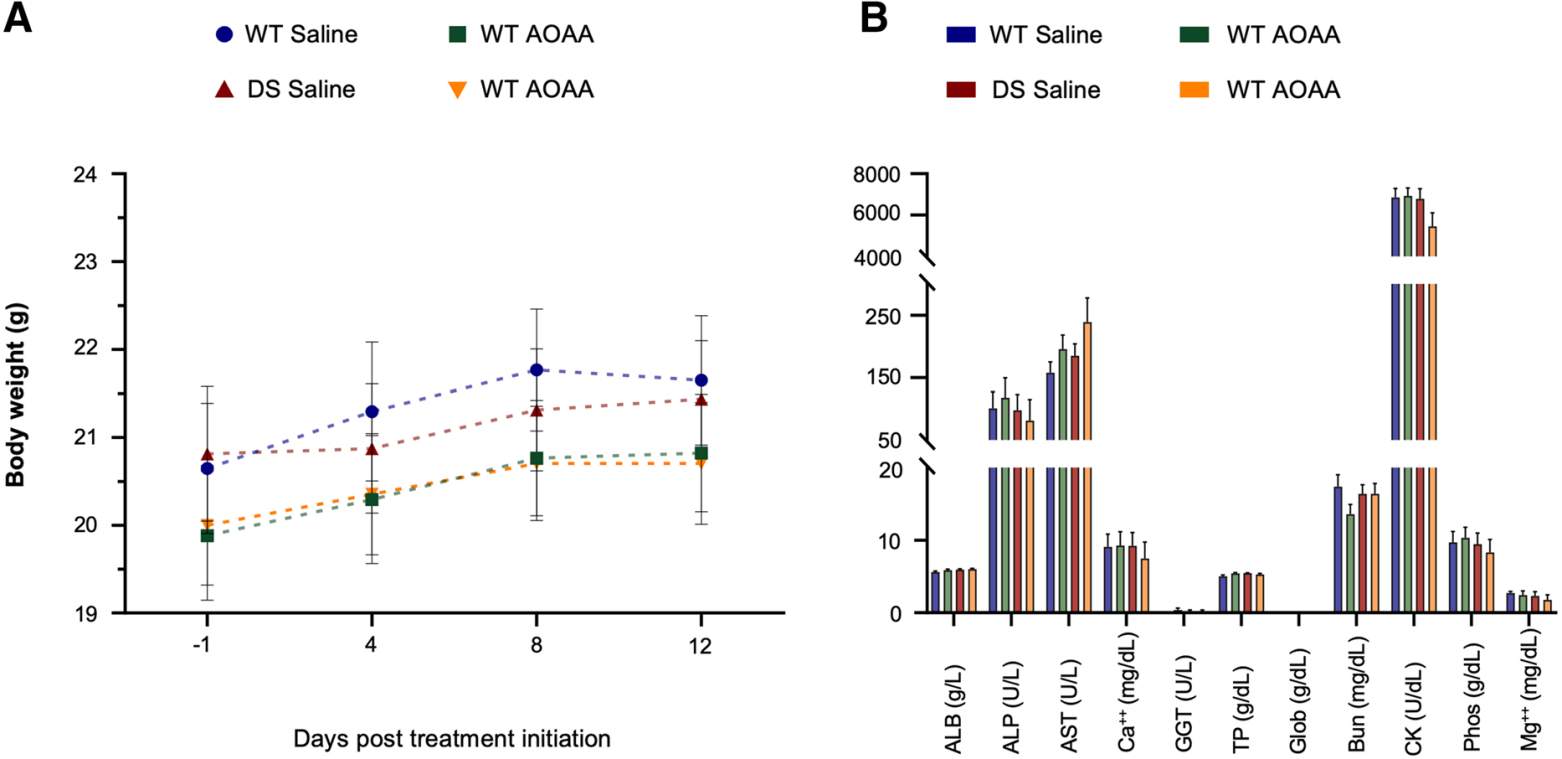
Pharmacological inhibition of the CBS enzyme with AOAA at a dose of 1 mg/kg/day for 2 weeks does not impact animal growth and systemic health. **A** Body weight of the mice monitored every 4 days; *n* = 16/group. **B** Analysis of circulating biomarkers for peripheral organ dysfunction, using the VetScan® Analyzer; *n* = 5/group. No statistically significant differences were found between the studied groups

**Fig. 10:**
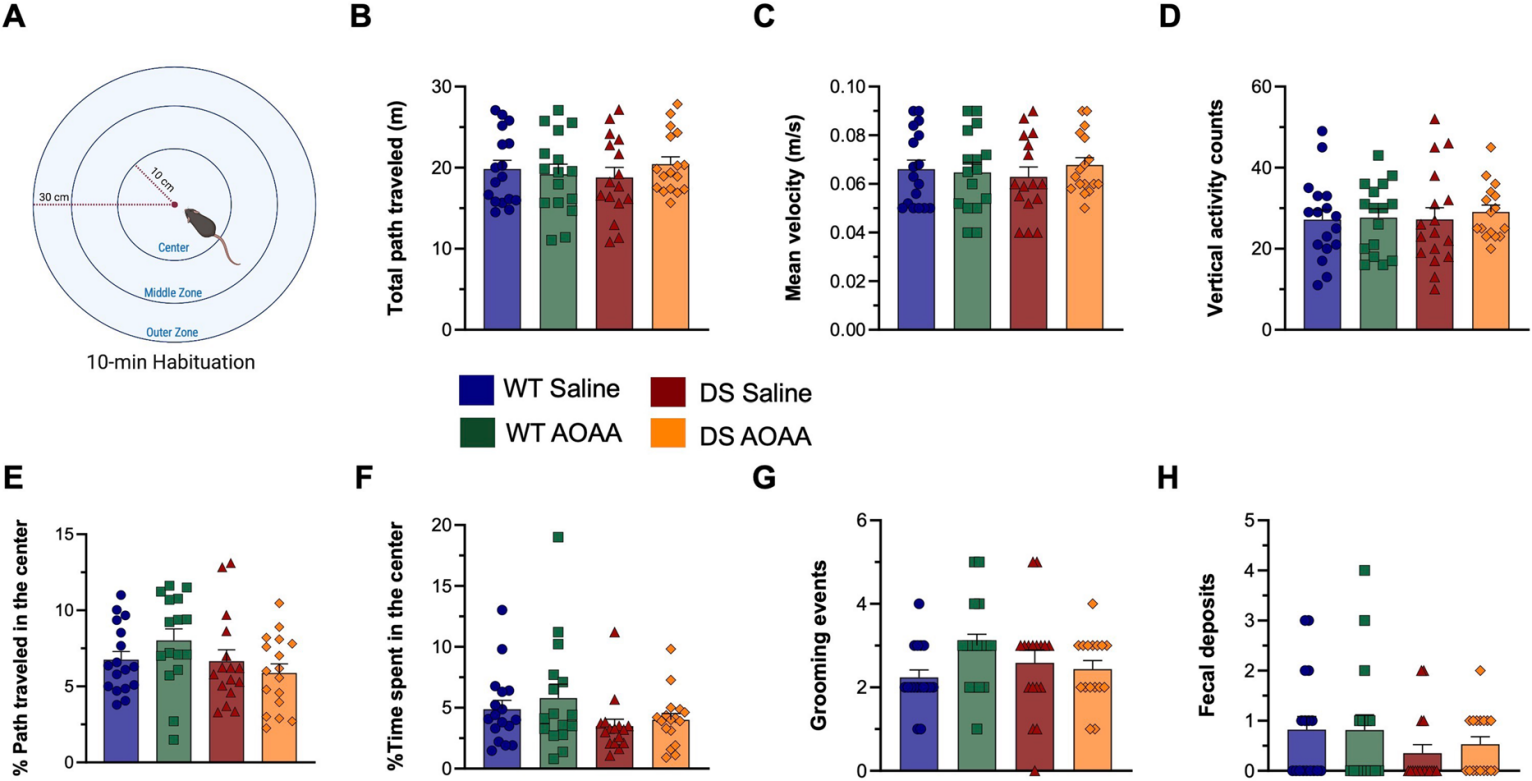
Dp(17)3Yey/ + mice exhibit normal locomotion and exploratory behavior. **A** Illustration of the open field arena annotated with the three individual zones utilized for the behavioral assessment along with the diameter of the total arena and central zone. **B** The total path traveled over the 10-min arena familiarization session along with **C** the mean velocity and **D** the vertical activity counts representing the activity and exploration of mice. In addition, **E** the percentage of the path traveled and **F** the time spent in the center of the arena along with the **G** grooming events and **H** fecal deposits were recorded to further appraise the emotional status and exploratory/risk-taking behavior of the mice. Each bar represents the mean ± SEM of 16 animals per experimental group, 8 male and 8 female; each dot represents one animal. No significant differences were found between the studied groups

**Fig. 11:**
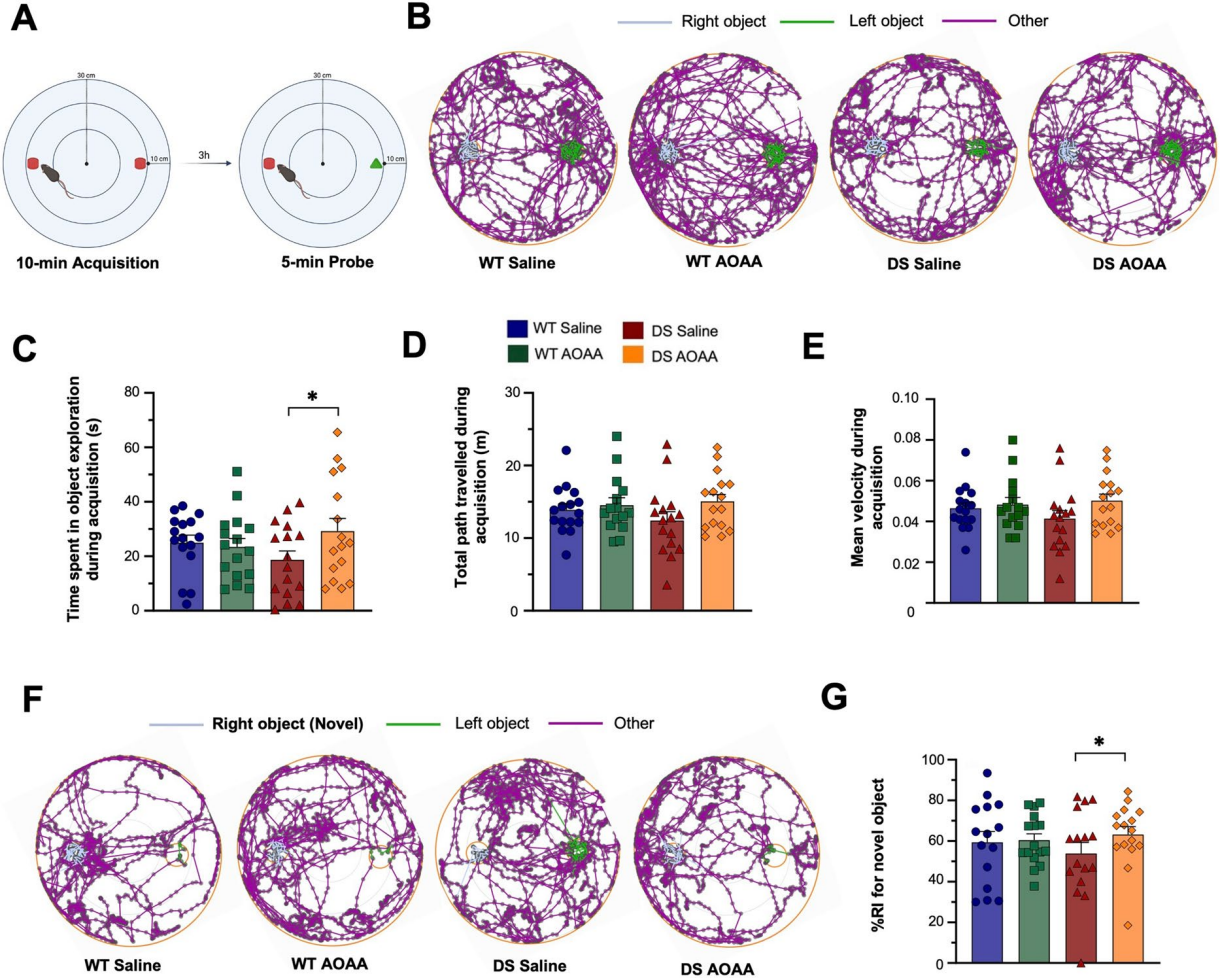
Recognition memory testing in the Dp(17)3Yey/ + mouse model; effect of AOAA treatment. **A** Illustration of the novel object paradigm used. **B** Representative tracking maps of animal activity in the arena during the 10-min acquisition session. **C** The total time spent in object exploration along with **D** the total path traveled and **E** the mean velocity was recorded during the acquisition trial. **F** Representative tracking maps of animal activity in the arena during the 5-min retention trial. **G** The recognition index (%RI) for the novel object—calculated as a percentage ratio of the time allotted to the novel object over the total object exploration—was employed for assessing recognition memory. Each bar represents the mean ± SEM of 16 animals per group, 8 male and 8 female; each dot represents one animal; *p ≤ 0.05

**Fig. 12:**
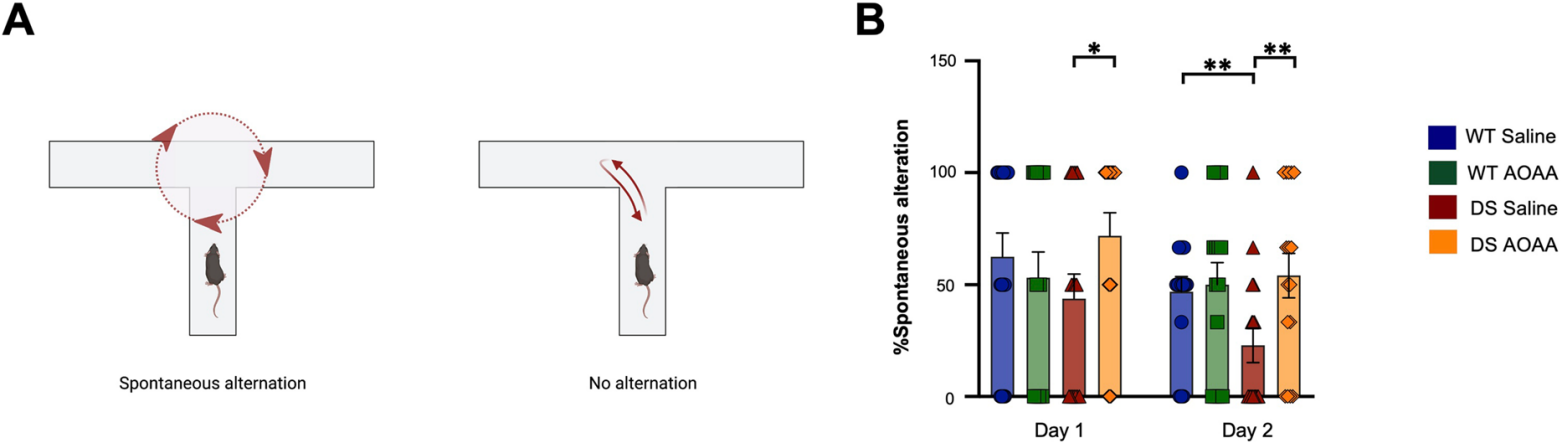
Suppressed spatial learning in the Dp(17)3Yey/ + mouse model; effect of AOAA treatment. **A** Illustration of the definition of spontaneous alternation in the T-maze paradigm, as used herein, that describes the natural tendency of the animals to enter the unfamiliar, previously unexplored arm of the apparatus. **B** The percentage of the spontaneous alteration as calculated over a total of five probe trials with a cut-off time of 90 s during a 2-day testing period per subject. Each bar represents the mean ± SEM of 16 animals per experimental group, 8 male and 8 female; each dot represents one animal; *p ≤ 0.05 and **p ≤ 0.01

The experimental design for the various functional assays is shown in Fig. 8. Treatment of the mice with AOAA did not affect their overall health and did not affect various plasma markers of organ injury (Fig. 9). DS mice exhibited normal locomotion and exploratory behavior, which was unaffected by AOAA treatment (Fig. 10). In the novel object recognition model (Fig. 11A), DS mice did not show a difference in the time spent in object exploration during acquisition (Fig. 11B, C), in the total path traveled during acquisition (Fig. 11B, D), or the mean velocity of during acquisition (Fig. 11B, E), further indicating normal locomotion and exploration. AOAA treatment did not affect most parameters in this phase, except the time spent in object exploration during acquisition, which was increased by the CBS inhibitor (Fig. 11C). In the recognition phase of the study, AOAA treatment of the mice did not affect RI performance in control mice but significantly improved this parameter in DS mice (Fig. 11F, G). In the T-maze model, which investigates spatial learning, DS mice exhibited impaired function, and the performance of the animals was improved by AOAA treatment (Fig. 12A, B).

### CBS inhibition improves synaptic function in the DS brain

**Fig. 13:**
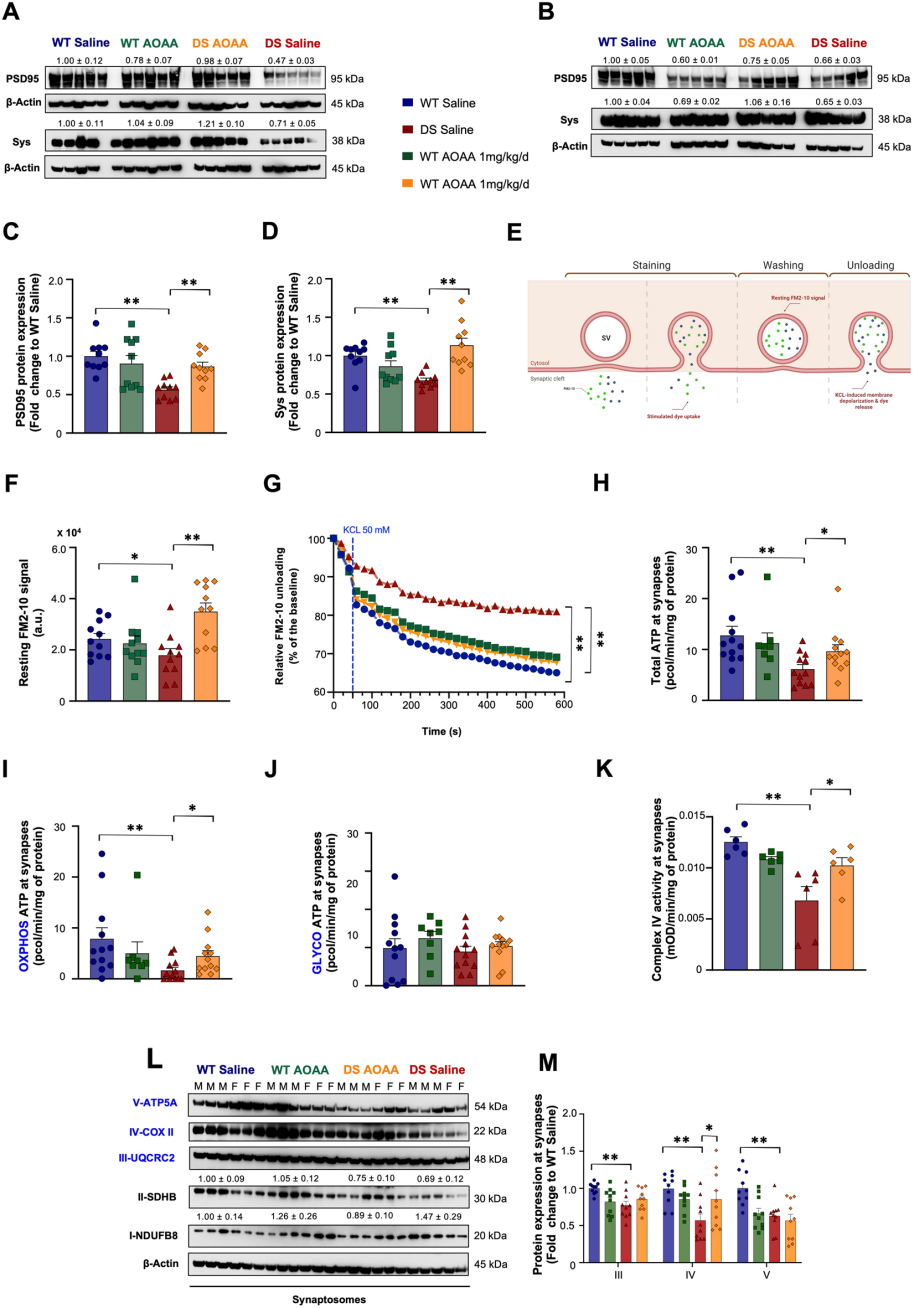
Pharmacological inhibition of CBS with AOAA rescues the impaired complex IV activity and complex IV expression in the synapses of the Dp(17)3Yey/ + mice and normalizes local ATP production, synaptic vesicle recycling, and the expression of major synaptic markers. Immunoblots for the expression of post-synaptic density protein of 95 kDa (PSD95) and synaptophysin (Sys) in whole-brain homogenates from **A** female and **B** male mice together with **C**, **D** densitometric analysis for each marker. **E** Illustration of the protocol for vesicle recycling by isolated synaptosomes using the fluorescent styryl dye FM2-10. **F** Resting fluorescent signal was monitored in single endpoint measurements to evaluate stimulated endocytosis and recruitment of synaptic vesicles (SVs) locally at synapses. Subsequently, **G** a second extracellular KCl treatment was employed to induce global depolarization and thus the release of the dye from the synaptic vesicles along with neurotransmitters. The fluorescent signal was subsequently monitored every second for a total time of 600 s, and the observed decay of the signal reflected the capacity of synapses to respond to the stimulus and engage exocytosis locally, indicative of their functionality. Quantification of **H** ATP production and **I** OXPHOS- and **J** glycolysis-derived ATP in isolated synaptosomes. Synaptosome preparations were further processed for enzymatic quantification of **K** the complex IV activity and densitometric quantification of **L** the protein expression level of OXPHOS complexes. β-actin served as the loading control in all densitometric analyses. **M** The bar graph of the OXPHOS densitometry displayed the complexes where statistically significant differences were observed. Each bar represents the mean ± SEM of animals per group (*N* = 10 for densitometry; *N* = 11 for FM2-10 fluorescent signal kinetics; *N* = 12 for extracellular flux analysis; *N* = 6 for complex IV activity); each dot represents one animal; *p ≤ 0.05 and **p ≤ 0.01

AOAA rescued the DS-associated suppression of PSD95 expression (Fig. 13A–C) and Sys protein expression (Fig. 13A, B, D) and improved the deficiency of the DS to release synaptic content upon stimulation (Fig. 13E–G). Since synaptic neurotransmitter release is an ATP-dependent process [43, 44] and DS is associated with a significant bioenergetic defect (6,7,11–13), we have also measured synaptosomal ATP content; this was reduced in DS and was improved by AOAA treatment (Fig. 13H). Bioenergetic characterization of the synaptosomes revealed a deficiency of oxidative phosphorylation (Fig. 13I)—but not of glycolysis (Fig. 13J) in DS; oxidative phosphorylation capacity of the synaptosomes was improved by CBS inhibition (Fig. 13I), similar to the previously demonstrated effect of CBS inhibition or CBS silencing in human DS fibroblasts [13, 15]. Mitochondrial complex IV activity and expression in the synapses of the DS mice were also suppressed, and CBS inhibition improved these responses (Fig. 13K–M)—once again, in line with previous data showing that overproduction of H_2_S in DS suppresses mitochondrial complex IV activity [13, 15]. There was also a significant downregulation of complex III and V expression in the DS synaptosomes, but this was unaffected by treatment of the mice with AOAA (Fig. 13L, M).

### CBS inhibition corrects several components of the unfolded protein response (UPR) in DS

**Fig. 14:**
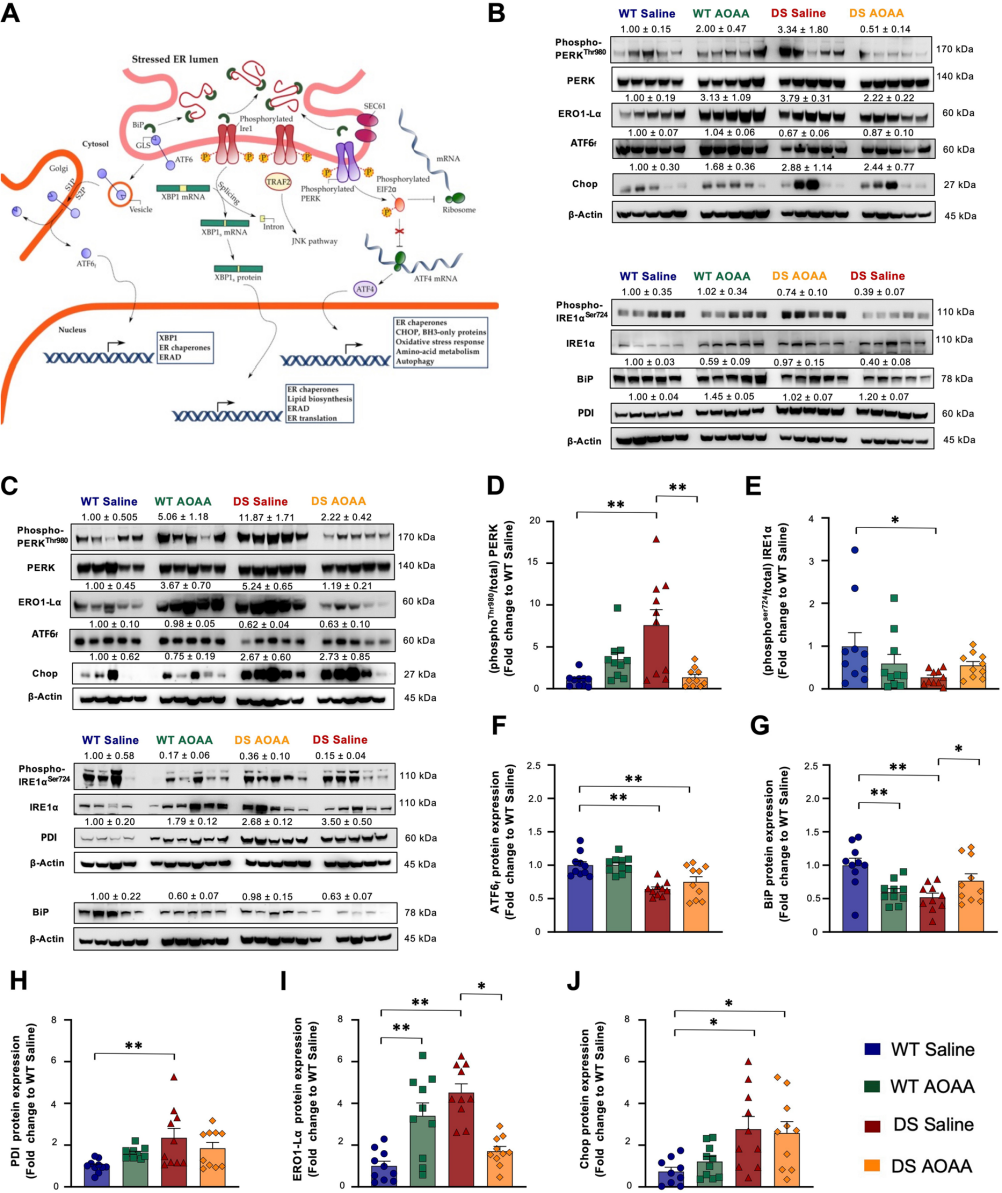
Pharmacological inhibition of CBS ameliorates the aberrant unfolded protein response in the Dp(17)3Yey/ + mouse brain. **A** Schematic illustration of the UPR signaling network. **B**, **C** Representative immunoblots of the UPR nodes quantified in whole-brain homogenates from **B** male and **C** female mice. Densitometric determination of the expression levels of **D** the active phosphorylated-to-the total PERK, **E** the active phosphorylated-to-the total IRE1α, and **F** the cleaved, active, 50-kDa fragment of the activating transcription factor 6 (ATF6f) along with the densitometric analysis for protein-quality control chaperones **G** BiP, **H** PDI, **I** ERO1-Lα, and **J** Chop. β-actin served as the loading control. Each bar represents the mean ± SEM of 10 animals per experimental group, 5 males and 5 females; each dot represents one animal; *p ≤ 0.05 and **p ≤ 0.01

**Fig. 15:**
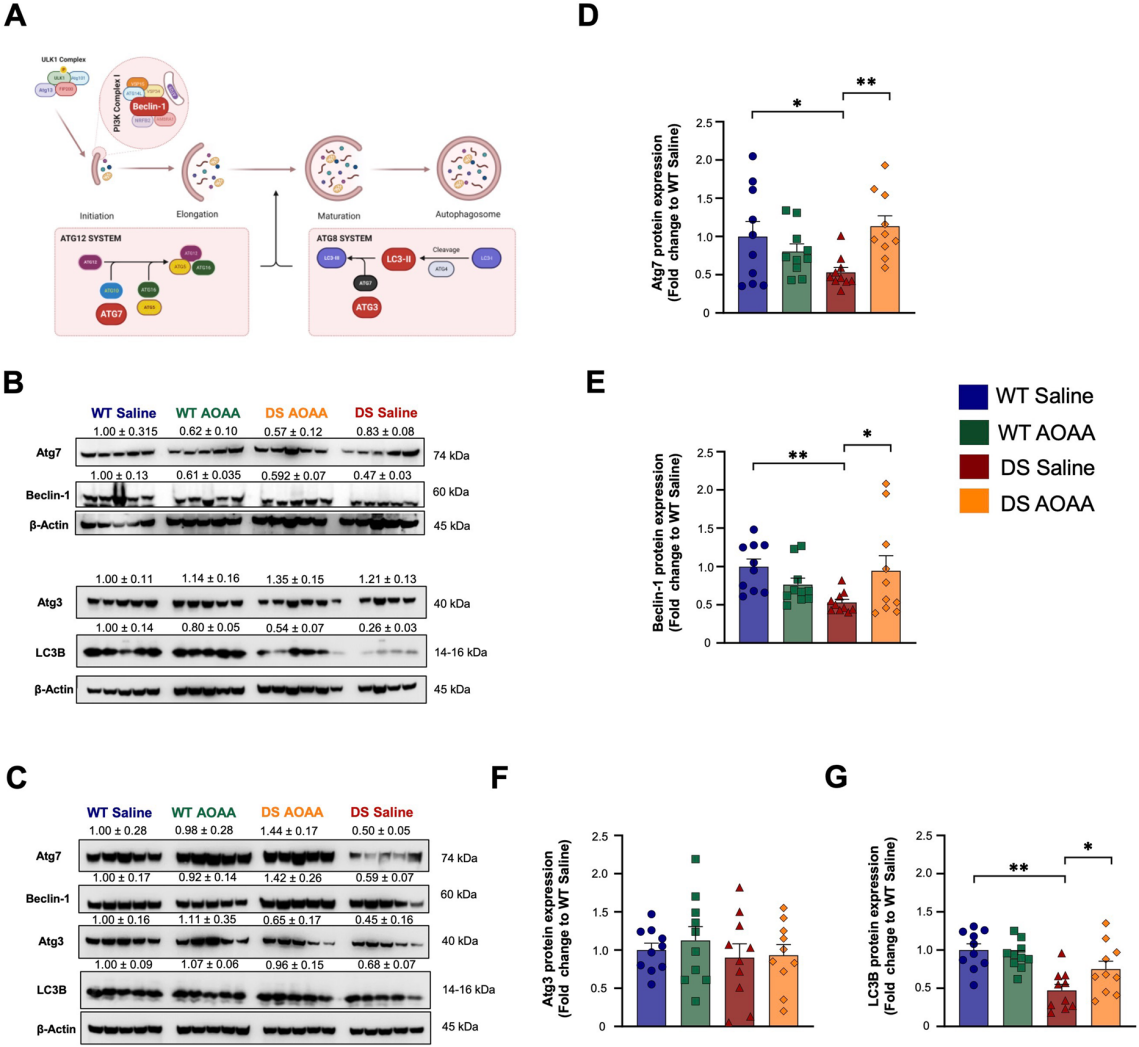
Pharmacological inhibition of CBS with AOAA ameliorates the availability of autophagy-related components crucial for autophagosome formation and maturation in the Dp(17)3Yey/ + mouse brain. **A** Schematic illustration of the autophagic machinery, with the investigated components of interest highlighted in red. Representative immunoblots of the selected rate-limiting components of the autophagy pathway quantified in whole-brain homogenates from **B** female and **C** male mice. Densitometric analysis of **D** Atg7, **E** beclin-1, **F** Atg3, and **G** LC3B proteins. β-actin served as the loading control. Each bar represents the mean ± SEM of 10 animals per experimental group, 5 males and 5 females, while each dot represents one animal; *p ≤ 0.05 and **p ≤ 0.01

Pharmacological inhibition of CBS enzyme with AOAA significantly affected the dysregulation of various unfolded protein response markers in Dp(17)3Yey/ + mice. Figure 14A shows the main pathways of this response, Fig. 14B, C shows representative Western blots, and Fig. 14D–J shows key UPR markers. PERK phosphorylation (Fig. 14B–D), the expression of PDI (Fig. 14B, C, H), the expression of ERO1-Lα (Fig. 14B, C, I), and the expression of the proapoptotic protein Chop (Fig. 14B, C, J) were higher in the DS brains than in the wild-type control brains. In contrast, expression levels of the phosphorylated serine/threonine-protein kinase/endoribonuclease IRE1α (Fig. 14B, C, E); the cleaved, active, 50-kDa fragment of ATF6 (ATF6f) (Fig. 14B, C, F); and BiP (Fig. 14B, C, G) were lower in the DS brain samples. In wild-type animals, the CBS inhibitor did not affect the levels of most factors—except BiP, which was slightly reduced (Fig. 14B, C, G). In contrast, treatment of DS mice with the CBS inhibitor normalized the expression level of several of the investigated UPR marker proteins (phosphor-PERK, ATF6f, BiP, ERO1-Lα) (Fig. 14B, C, F, G, I). There were also significant changes in the autophagosomal response in DS (Fig. 15). Atg7 (but not Atg3), beclin-1, and LC3B expressions were lower in the brain homogenates of DS mice than in wild-type controls and were increased after treatment of the mice with the CBS inhibitor (Fig. 15).

### Quantification of H_2_S generation, H_2_S levels, and persulfidated protein levels in the DS brain

In whole-brain homogenates, when CBS activity was maximally activated with the addition of the CBS allosteric activator SAM and the substrate of CBS, AzMC-guided detection of H_2_S generation was performed; H_2_S generation in DS brain samples was 172 ± 3% of the wild-type control (*n* = 6, *p* < 0.05). Likewise, when the tissue’s ability to generate H_2_S from maximally activated 3-MST was quantified, the response in DS samples was 146 ± 16% of the wild-type control (*n* = 6, *p* < 0.05). These results are consistent with the higher expression of the respective enzymes CBS and 3-MST in DS brains than in control brains, already demonstrated by Western blotting shown in Fig. 2.

Plasma H_2_S levels in wild-type and DS mice were quantified as 78 ± 4 nmol/l (*n* = 8) and 95 ± 12 nmol/l (*n* = 8), respectively: a statistically significant (*p* < 0.05) difference (Table 1). H_2_S levels in the DS brain homogenates amounted to 126 ± 19% of wild-type controls (*n* = 8), while polysulfides in the DS brain homogenates amounted to 90 ± 14% of wild-type controls (*n* = 8) (Table 1).

**Table 1:** Differences in H_2_S and polysulfide levels and related small molecule species between control and DS mouse brains (B) and plasma (P) samples. For brain values (B), data are expressed as mean ± SEM of *n* = 4/group; control values (male + female grouped together) were set as 100%. For plasma values (P), absolute values (nmol/l) are shown

	WT (♂)	WT (♀)	DS (♂)	DS (♀)	DS/WT (%)
H_2_S (B)	106 ± 23	94 ± 19	85 ± 5	170 ± 5*	128 ± 28
H_2_S_2_ (B)	119 ± 46	81 ± 20	64 ± 5	116 ± 21	90 ± 14
Cys (B)	107 ± 24	93 ± 26	85 ± 7	171 ± 41	128 ± 25
Cys-SH (B)	113 ± 30	88 ± 18	84 ± 8	167 ± 26	126 ± 20
GSH (B)	102 ± 18	98 ± 21	87 ± 3	205 ± 64	146 ± 37
GSSH (B)	108 ± 21	92 ± 22	86 ± 8	153 ± 26	120 ± 18
H_2_S (P)	86 ± 3	70 ± 6	100 ± 14	90 ± 20	122 ± 16

^*^p < 0.05

**Fig. 16:**
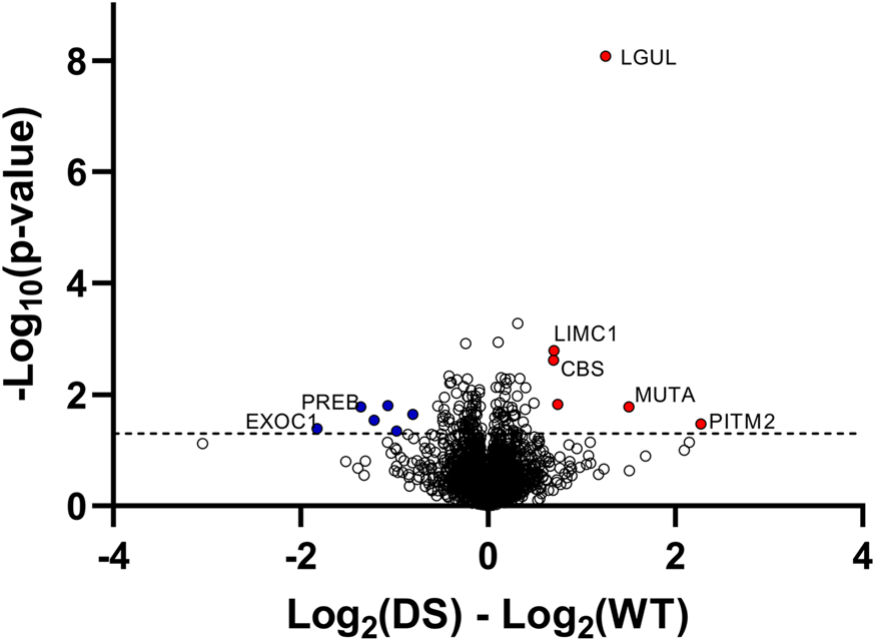
Total proteome comparison of wild-type vs. DS brains. Wild-type male mice (WTm, *n* = 3) and wild-type female mice (WTf, *n* = 3) were analyzed together as a single group; DS male mice (DSm, *n* = 3) and DS female mice (DSf, *n* = 4) were also analyzed together as a single group

Next, we assessed the changes in proteome and protein persulfidation levels. We first monitored the total proteome changes between DS and wild-type control mice. As expected, CBS was found to be upregulated at the protein level (Table S2), confirming the Western blot data shown in Figs. 2 and 3. CBS was the only protein encoded on the extra chromosome 17 segment that was detected by the mass spectrometry analysis; the expression level of the other 17 gene products relevant for the current mouse model (UMODL1, ABCG1, TFF1, TFF2, TFF3, TMPRSS3, UBASH3A, RSPH1, SLC37A1, PDE9A, WDR4, NDUFV3, PKNOX1, U2AF1, CRYAA, SIK1, HSF2BP, RRP1B) [21, 22] did not reach the detection limit of our analysis. Out of 3123 quantified proteins (5/5 confident hits per group), 128 showed significant change (54 upregulated and 74 downregulated) in DS brain samples when compared to wild-type control mice (Fig. 16).

**Fig. 17:**
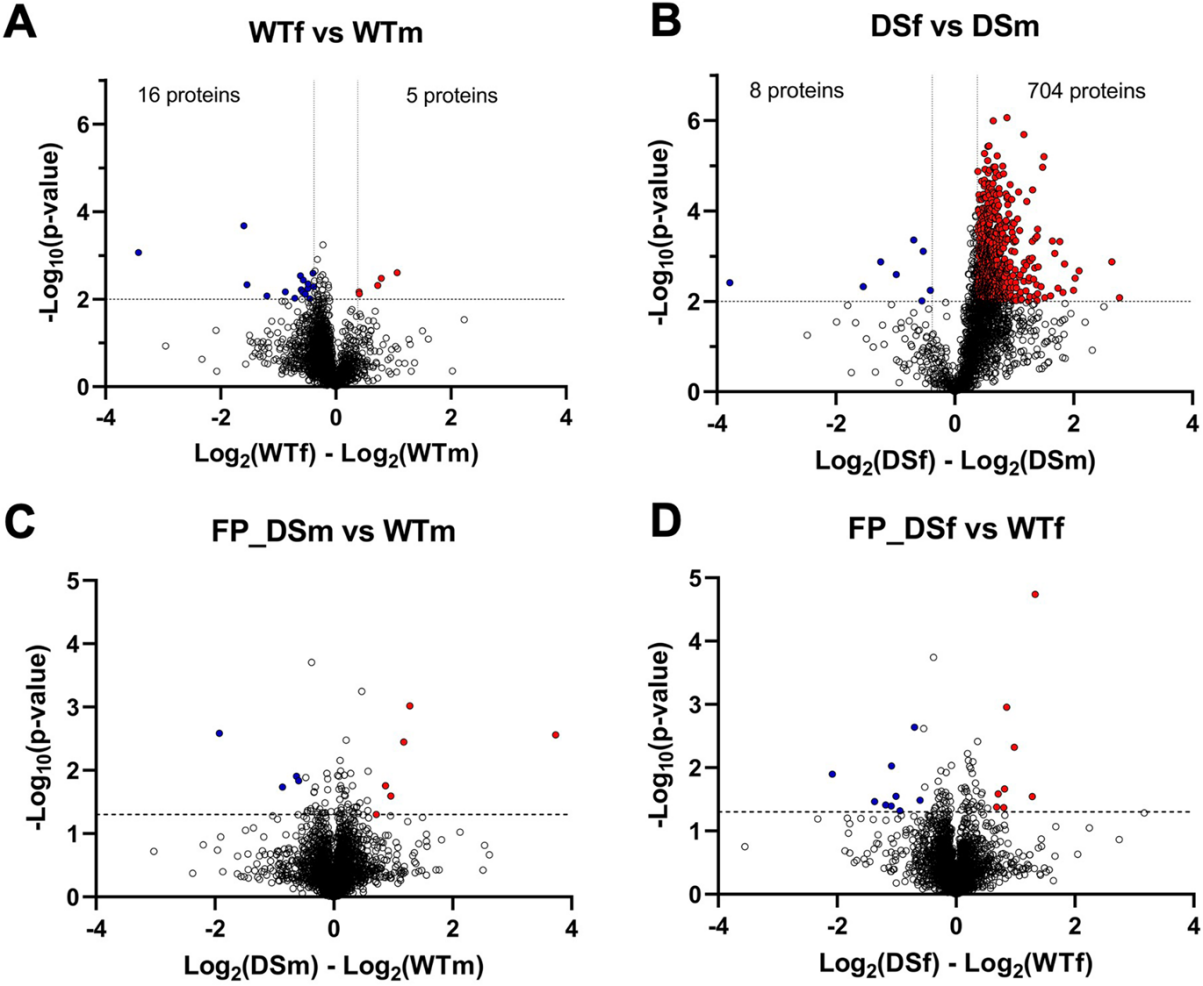
Total proteome comparison of wild-type vs. DS brains: analysis of potential sex differences. **A** Comparison of male vs. female wild-type mice; **B** comparison of male vs. female DS mice; **C** comparison of male wild-type mice (WTm, *n* = 3) vs. male DS mice (DSm, *n* = 3); **D** comparison of female wild-type mice (WTf, *n* = 3) vs. female DS mice (DSf, *n* = 4). Volcano plot of log2-transformed ratio (fold change) against the negative log10 of the *p* value calculated using Welch’s test. Horizontal gray line denotes *p* value cut-off of 0.01

Some of the markedly upregulated proteins in DS include lactoylglutathione lyase (LGUL) and methylmalonyl-CoA mutase (MUTA)—both of which are also encoded by mouse chromosome 17, although not by the segment that contains an extra copy in the current model—LIM and calponin homology domain–containing protein 1 (LIMC1) and membrane-associated phosphatidylinositol transfer protein 2 (PITM2), both of which are encoded on mouse chromosome 5 (Fig. 17, Table S2). Pathway enrichment analysis showed no pathway passing the Benjamini significance threshold. Proteins that differ in their expression between wild-type and DS mice were predominantly located in the cytosol (44 proteins), membrane (67 proteins), and mitochondria (44 proteins).

**Fig. 18:**
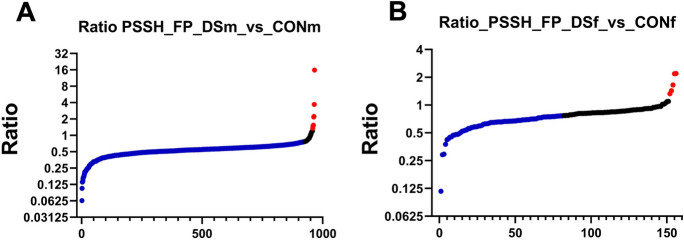
Changes in persulfidated proteins in the brain of DS mice, normalized to protein levels: analysis of potential sex differences. **A** Comparison of male wild-type mice (WTm, *n* = 3) vs. male DS mice (DSm, *n* = 3); **B** comparison of female wild-type mice (WTf, *n* = 3) vs. female DS mice (DSf, *n* = 4)

Using the dimedone switch method for global persulfidome analysis [30, 45], we next compared the changes in protein persulfidation of DS brains. In DS brains, protein persulfidation was lower than in wild-type controls (Fig. 18, Table S3)—as opposed to the expected increase that is typically seen in pathophysiological states associated with increased H_2_S biosynthesis [46–48].

### Metabolomic analysis of the DS mouse brain and the effect of AOAA treatment

**Fig. 19:**
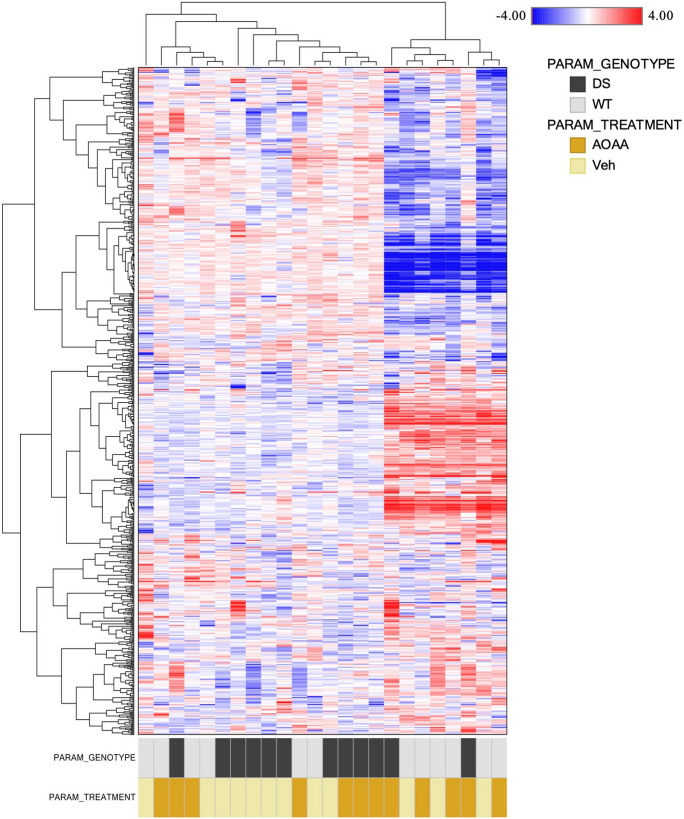
Hierarchical clustering of the brain samples from the 4 groups (wild-type mice, wild-type mice treated with AOAA, and DS mice treated with AOAA, *n* = 6 each) is shown (Euclidean distance, complete correlation)

In whole-brain homogenates, following normalization to protein concentration and log transformation, an untargeted metabolomic dataset—comprises a total of 731 biochemicals, 690 compounds of known identity (named biochemicals), and 41 compounds of unknown structural identity (unnamed biochemicals)—has been generated for brain samples. Analyte concentrations measured in the four groups (wild-type mice, wild-type mice treated with AOAA, and DS mice treated with AOAA) were compared. Hierarchical clustering of the analytes per treatment group is shown in Fig. 19. A summary of the numbers of biochemicals that achieved statistical significance (*p* ≤ 0.05), as well as those approaching significance (0.05 < *p* < 0.10), is shown in Table S4.

**Fig. 20:**
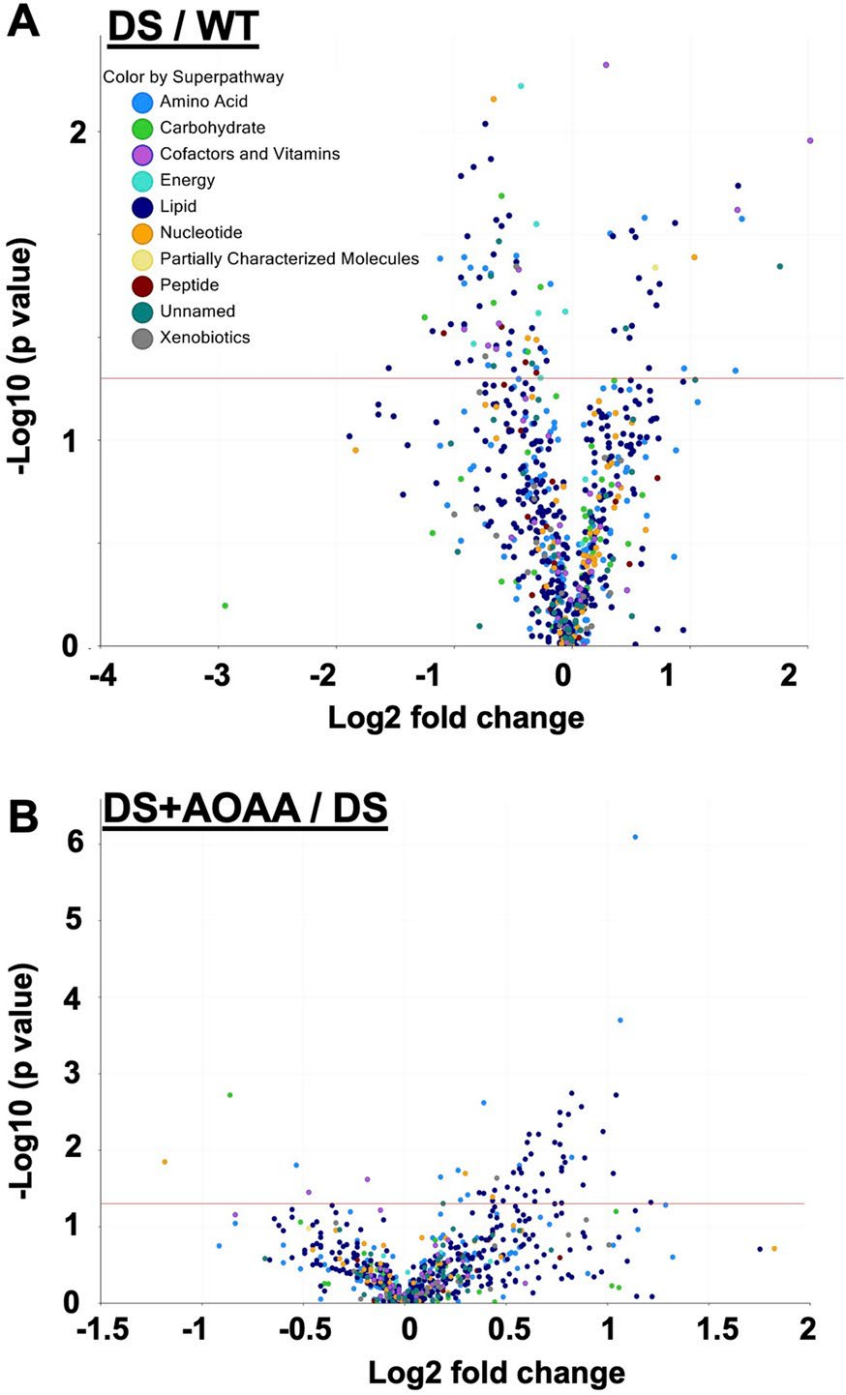
Volcano plots showing differences in various analytes measured by untargeted metabolomic analysis between **A** DS vs. wild-type mouse brains or **B** AOAA-treated DS mice vs. DS mice brains

Volcano plots (Fig. 20) demonstrate that the metabolomic profile of DS mouse brains is significantly different from wild-type controls. There were 96 analytes that were statistically significantly (*p* < 0.05) affected: 25 analytes were higher in DS than in control; 71 analytes were lower in DS than in control. AOAA treatment of the wild-type mice did not induce marked changes in the metabolomic profile, with only 9 analytes affected significantly (Table S4). In contrast, in DS mice, AOAA significantly (*p* < 0.05) affected 56 metabolites, the majority of which (51 analytes) were higher in the brains of AOAA-treated DS mice than in the vehicle-treated DS mice (Fig. 20).

In the subsequent analysis, therefore, we have focused on the differences between analytes in DS and wild-type brains and on the effect of AOAA in DS brains. From the various metabolite clusters, some of the most relevant differences were observed in various amino acid metabolites. Selected examples of these are shown in Table S5. Glycine levels were lower in DS brains than in wild-type brains, and AOAA treatment of DS mice tended to partially reverse this alteration. For serine, the opposite trends were noted (serine levels in DS brains were higher than in wild type, and AOAA tended to lower these levels). From analytes related to the histidine metabolism, homocarnosine levels were lower in DS mice than in the wild type, and AOAA treatment of DS mice reversed this difference. Dopamine levels as well as the levels of its primary metabolite homovanillate were significantly higher in DS brains than in wild-type brains, and AOAA treatment of DS mice reversed this difference. From the urea cycle/arginine metabolism, arginine and arginosuccinate levels were lower in DS brains than in wild-type brains, and AOAA treatment in DS mice tended to reverse this difference; citrulline levels trended to show the same pattern of changes as well. From the polyamine metabolism, putrescine and its metabolite N-acetylputrescine tended to be lower in DS brains than in wild-type brains, and AOAA tended to reverse this difference (Table S5).

With respect to carbohydrate metabolism and the “energy subpathway,” the differences are summarized in Table S6. The detected glucose levels in DS brains were markedly lower than in wild-type brains. Phosphoglycerate levels tended to be higher in DS brains and lower in AOAA-treated DS brains. From the pentose phosphate pathway, the most significant difference was the low sedoheptulose-7-phosphate levels in the DS brain with a trend for AOAA to partially attenuate this difference. Also, 6-phosphogluconate levels trended higher in DS control compared to wild-type control, while AOAA treatment of DS animals caused a decrease in this metabolite. From the Krebs cycle, most of the metabolites in this cycle (citrate, aconitate, succinylcarnitine, fumarate, and malate) were lower in DS brains than in wild-type brains. AOAA treatment of DS mice tended to slightly reverse these alterations, but the effect did not reach the level of statistical significance.

DS brains exhibited marked differences in terms of multiple analytes in the lipid subpathway (Table S7), in many cases 30–50% lower levels than levels measured in wild-type brains for long chain mono- and poly-unsaturated fatty acids, monohydroxy fatty acids, and lysophospholipids. Interestingly, AOAA treatment of DS mice tended to reverse many of these alterations, as it was the case, for instance, for 10-heptadecenoate, eicosenoate, erucate, eicosapentaenoate, docosapentaenoate, nisinate, docosadienoate, stearoylcarnitine, behenoylcarnitine, 2-hydroxyheptanoate, 2-hydroxystearate, 2-hydroxybehenate, 2-hydroxynervonate, 3-hydroxylaurate, 1-oleoyl-GPE, 1-linoleoyl-GPE, 1-oleoyl-GPS, 1-palmitoyl-GPG, 1-stearoyl-GPG, 1-oleoyl-GPG, 1-oleoyl-GPI, 1-linoleoyl-GPI, 1-oleoyl-GPI, and 1-linoleoyl-GPI. There were also marked changes in the DS brain in terms of multiple endocannabinoid metabolites (typically decreases); in several instances, these alterations were reversed in the brains of the AOAA-treated DS mice (Table S8).

With respect to differences in the nucleotide subpathway (Table S9), significant differences were found in purine and pyrimidine metabolism and nicotinate and nicotinamide metabolism. ADP and AMP levels tended to be higher in DS brains than in wild-type brains, with AOAA trending to ameliorate this difference in DS brains. N6-succinyladenosine levels were low in DS brains but were increased in AOAA-treated DS brains. Uridine 5′-diphosphate levels (and, to a lesser degree, uridine 5'-monophosphate) exhibited the same pattern. Both NAD^+^ and NADH levels were higher in DS brains than in wild-type brains, but the difference in the former was more pronounced than in the latter, so the NAD^+^/NADH ratio can be calculated to be lower in DS brains than in wild-type controls. AOAA treatment in DS mice decreased NADH levels but affected NAD^+^ levels to a smaller degree, the end result being a higher (i.e., improved) NAD^+^/NADH ratio in DS brains.

### Post hocassessment of sex differences in the observed responses DS brain

The measurements of brain H_2_S levels and the changes in protein persulfidation levels demonstrated a clear sex difference in the responses. Sex differences were not part of our original design, and our animal groups contained an equal number of male and female mice and were planned for pooled analysis in our original statistical power calculations. However, we have noticed that the trend for the difference in H_2_S levels in the DS brain samples was driven predominantly by the female samples, where H_2_S levels were 70% higher (*p* < 0.05) in DS than in wild-type controls (Table 1). This difference was detected in the brain, but not in circulating H_2_S levels (Table 1). Thus, the sex dependence in the H_2_S levels occurs in the brain of DS mice but may not necessarily represent a whole-body phenomenon.

A marked sex difference was also noted in the persulfidation responses. While no noticeable sex differences were observed in the proteome of the wild-type mice (Table S2), more proteins were found to change in female than in male DS brain samples. Importantly, the upregulation of CBS in female DS brains was more pronounced than in male DS brains (Fig. S1, Table S2). This observation was also confirmed by post hoc analysis of the Western blot data, where CBS expression in female DS mice was higher than in male DS mice, both in whole-brain analysis (Fig. S1) and in synaptosomes for the cytosolic, but not for the synaptic protein (Fig. S2). Interestingly, the upregulation of 3-MST also showed a sex difference; it was higher than the wild type in the female, but not in male DS mice whole-brain samples (Fig. S1B) but not in the synaptosomes (Fig. S2B).

**Fig. 21:**
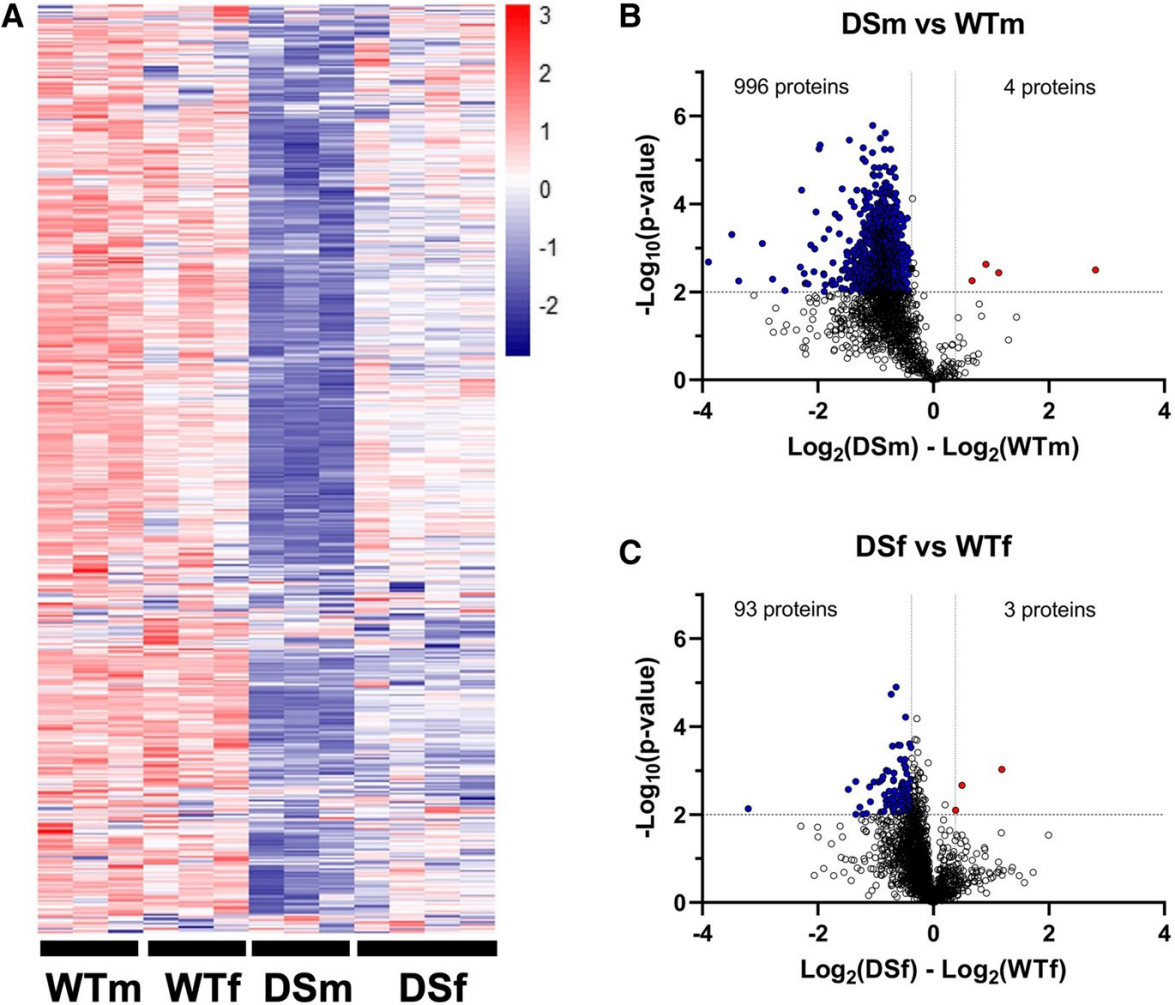
Changes in persulfidated proteins in the brain of male vs. female DS mice. **A** Heat map representing changes in persulfidation levels in wild-type male mice (WTm, *n* = 3), wild-type female mice (WTf, *n* = 3), DS male mice (DSm, *n* = 3), and DS female mice (DSf, *n* = 4). **B** Volcano plot of log2-transformed ratio (fold change) against the negative log10 of the *p* value calculated using Welch’s test. Horizontal gray line denotes *p* value cut-off of 0.01 and vertical ones the fold change cut-off of 1.3

The baseline persulfidation of brain proteins did not show any marked sex difference in wild-type mice (Table 1). However, persulfidation was markedly higher in male DS brains compared to male wild-type brains (where 996 proteins showed significant decreases in their persulfidation). In female brains, the difference was less pronounced: only 93 proteins showed a decreased persulfidation (Fig. 21). Normalization had little effect on persulfidation data obtained for the male DS brains, but it did slightly reduce the number of significantly decreasing proteins in female DS brains (Table S10).

Proteins with markedly decreased persulfidation in male DS mice include unconventional myosin-XVIIIa, Ras-related protein Rap-2a, ubiquitin conjugation factor E4 B, the RNA-binding protein vigilin, adenylate kinase isoenzyme 5, and aldehyde dehydrogenase family 3 member B1. Proteins with markedly decreased persulfidation in female DS mice include ubiquitin-conjugating enzyme E2 J1, tubulin alpha-8 chain, protein transport protein Sec16A, and heterogeneous nuclear ribonucleoprotein H2.

Higher persulfidation in DS compared to wild-type control was a relatively rare event (Fig. 21). Proteins with higher persulfidation in female DS brains included Arf-GAP with SH3 domain, ANK repeat and PH domain–containing protein 1, vigilin, endophilin-A2, adenylate kinase isoenzyme 5, and PRRC2A. Proteins with increased persulfidation in male DS brains included ubiquitin-conjugating enzyme E2 J1, thioredoxin domain–containing protein 5 homolog, and two heterogeneous nuclear ribonucleoproteins, H1 and H2. After the persulfidation, changes were normalized to the actual protein levels quantified by total proteome measurements, and the global effect remained the same (Fig. 18).

Since persulfidation can either increase or decrease the activity (or perhaps also the stability) of a given protein—but it can also be functionally silent—the functional effect of this posttranslational modification may be dependent (a) on the protein in question and (b) the site and functionality of the persulfidated cysteine within the protein structure. The functional role of persulfidation, for the proteins identified in the current study, remains to be systematically assessed. Thus, it is difficult to derive specific hypotheses on the functional role and importance of each persulfidation detected in our study. We can assume that pathways where multiple proteins are affected may be more affected functionally than pathways where only a few proteins are affected—although there may be exceptions to this rule, in case a unique and functionally crucial protein is affected. As a first approximation, we present Kyoto Encyclopedia of Genes and Genomes (KEGG) pathway enrichment tables which depict the core pathways where proteins with altered persulfidation patterns in DS were identified. These pathways (Fig. S3) include the Krebs cycle, the valine, the leucine and isoleucine degradation pathway, pathways of pyruvate metabolism, fatty acid degradation, and the proteasomal protein processing. Combined KEGG pathway enrichment data are presented in Table S11.

With respect to post hoc assessment of the effect of AOAA treatment on reactive astrocytosis, no sex difference was observed (Fig. S4A). Also, there was no sex difference in the degree of branching of the astrocytes in the DS brain (Fig. S4B).

Post hoc re-assessment of the functional neurobehavioral data indicated that in the recognition memory testing model (Fig. S5), the increasing effect of AOAA on the time of DS mice spent for exploration during acquisition was primarily driven by an effect in female DS mice (Fig. S5A). Moreover, the DS-associated impairment in %RI was only noted in the female animals (Fig. S5B), and the beneficial effect of the CBS inhibitor was also only statistically significant in the female subgroup (Fig. S5B,C). However, in the spatial learning model, it was the male cohort of DS mice that tended to respond better to the CBS inhibitor than the female cohort (Fig. S6).

With respect to potential sex differences in the ER stress responses, the parameters that were more affected by AOAA in DS in females than in males were PERK phosphorylation, the expression of PDI, and ERO-1Lα (Fig. S7). It was also the female subgroup that responded better to CBS inhibition in terms of ER stress parameters: in the female—but not male—cohort (Fig. S7). Post hoc analysis shows that the CBS inhibitor exhibited more pronounced effects in female DS mice than in male DS mice on the expression of many of the factors investigated in the analysis of UPR and autophagy. For example, for phospho-PERK, the effect of AOAA in DS was borderline significant in males (*p* = 0.058), while in females, it was highly significant (*p* < 0.01). For PDI expression, in male DS, no overall significant effect was noted nor any effect in males (*p* = 0.521), but if females were analyzed separately, a trend toward normalization was noted (*p* < 0.08). For ERO1-Lα expression, the effect of AOAA in male DS animals was borderline significant (*p* = 0.058), while in females, it was highly significant (*p* < 0.01). Likewise, for Atg7 and beclin-1, the difference between the DS and DS + AOAA subgroups was highly significant in the female DS group (*p* < 0.01), while when the corresponding expression levels were compared in the male DS group, no significant differences were detected. The only analyte in the UPR/autophagy group of proteins that was more affected by AOAA in DS in males than in females was the expression of BIP. The expression of this protein was markedly increased by AOAA in male DS brains (*p* < 0.01), while no significant effect of AOAA on BIP expression was observed in female DS brains (Fig. S7).

Given the significant male/female differences in brain CBS/3-MST expression, brain H_2_S levels, protein persulfidation change patterns, and some of the neurobehavioral effects noted in the preceding sections, it would be important to determine if sex differences are present in the metabolomic responses. It must be emphasized in advance that in the metabolomics studies, each group consisted of 3 male and 3 female animals and was originally designed to be analyzed as a single group. Thus, the metabolomic analysis was not powered to look at sex differences in the responses. Nevertheless, we have conducted a post hoc analysis of metabolites where the metabolite level in the DS + AOAA group was significantly different from the DS control levels (Table S4). With respect to the effects of AOAA on glycine and dopamine/homovanillate levels, it appears that the effects of AOAA were predominantly elicited by effects in the male animals (in male DS animals, AOAA induced a 78% increase and a 68/58% decrease in glycine and dopamine/homovanillate levels, respectively; *n* = 3).

Regarding changes in glycolytic and Krebs cycle metabolite levels, the AOAA effects in DS, once again, were somewhat sex-dependent: the trends/effects in the normalization of citrate, aconitate, succinylcarnitine, fumarate, and malate were primarily driven by effects in male animals (13, 23%, 70%, 24%, and 20% increases, respectively; *n* = 3). Similarly, the effect of AOAA in DS mice on various endocannabinoid levels was mainly due to changes in the male cohort (increases in oleoyl, palmitoyl, and stearoyl ethanolamide levels by 108%, 119%, and 42%, respectively; *n* = 3).

The AOAA-induced changes in NAD^+^ and NADH levels in the DS brains were also mainly driven by changes in the male animals: in the brains of male DS animals, AOAA caused 39% and 67% decreases, respectively, while in female animals, the respective changes were a 20% increase and a 25% decrease (*n* = 3). The effect of AOAA on homocarnosine levels in DS brains was more pronounced in the male cohort (a 109% increase), but it was also present in the female DS brains (a 45% increase). In contrast to the above differences, the effect of AOAA in DS brains on the arginine, proline, polyamine, pentose phosphate, purine, and pyrimidine nucleotides did not reveal any detectable sex differences.

## Discussion

The results of the current report confirm and extend the pathophysiological role of the CBS/H_2_S pathway in DS. In the current study, we used the Dp(17)3Yey/ + mouse model—which does not contain an extra copy of *all* of the mouse genes that are homologous to human chromosome 21 but does contain an extra copy of the fragment of mouse chromosome 17 that encodes CBS. In the DS literature, these animals are relatively rarely used; the majority of the published reports using DS mouse models utilize animals that have an extra copy of many mouse regions that are homologous to human chromosome 21—but, importantly, they do not contain an extra copy of the *cbs* gene. These common DS mouse models include the Ts16 mouse which contains an extra copy of mouse chromosome 16, containing a large region of genes syntenic to those on human chromosome 21, as well as many genes that are not represented on human chromosome 2, and the Ts65Dn mouse which consists of the translocation of the distal region of mouse chromosome 16 onto the centromeric region of mouse chromosome 17 [49–51]. Thus, clearly, the pathomechanisms described in these models are unlikely to be related to the over-activation of the CBS/H_2_S pathway. This is an important point and should be kept in mind when interpreting the current findings and contrasting them to the published body of DS literature that utilizes murine models of the condition.

The published body of literature regarding the Dp(17)3Yey/ + mice is relatively small and—thus far—has not investigated the CBS/H_2_S pathway. According to the published literature, the Dp(17)3Yey/ + mice do not show significant defects in Morris water maze tests or contextual fear conditioning tests, but they exhibit increased hippocampal long-term potentiation responses [21, 22]. To our knowledge, no prior studies have examined the performance of these animals in novel object recognition assays nor have any sex differences been studied or noted previously. The most closely related mouse model is the Dp(17Abcg1-cbs)1Yah model, which encodes mouse homologs of 11 genes that are encoded on chromosome 21 in human DS (including CBS). In this model, there is a significant defect in novel object recognition performance, which is normalized after genetic correction of CBS overexpression [12].

Regarding the role of the CBS pathway in DS, there is already a significant amount of information in the literature, both in human studies and in animal models. Clinical studies showed that increased excretion of thiosulfate (the major metabolite of H_2_S) has been noted in DS individuals (from mean values of 2.6 mmol/mol creatinine in the control group to 5.6 mmol/mol creatinine in the DS group) [8, 9]. The range of the thiosulfate levels in the urine showed a very marked inter-individual variation in DS (from 1.6 to 20.3 mmol/mol creatinine) [8, 9]. In the above studies, sex differences were not investigated or reported. In the current study, both circulating and brain H_2_S levels were found to be higher in DS than in wild-type controls, with the effect mainly carried by the female mice in the total animal group. Sex differences were also reflected in the persulfidation patterns of the brain proteins in DS (see below).

Regarding the expression of CBS in DS, the literature is extensive and includes human DS clinical materials as well as various cell lines from DS individuals and shows a markedly higher level of CBS in the DS cells [16, 17]. In addition, CBS expression is higher in the brain of DS rats (an animal model of DS which contains an extra copy of *all* rat genes that are homologous to those encoded by human chromosome 21) than in wild-type control rat brains [14]. Similar to the findings of the current study, CBS in the rat DS brains was primarily localized to the astrocytes and was associated with significant astrocytosis (higher astrocyte numbers, likely a reactive response). Likewise, in human DS brain samples, high CBS levels and primarily astrocytic CBS localization were reported [51, 52]. Astrocytosis is also well documented in clinical DS brains and represents a dynamic process [3–5, 53]. Based on the results of the current report, astrocytosis in the DS brains can be reversed by the CBS inhibitor AOAA, a response, which suggests that CBS-mediated H_2_S overproduction may play a stimulatory role in astrocyte proliferation. Indeed, multiple in vitro studies demonstrate that H_2_S can stimulate the proliferation of various cell types—although, similar to other effects of H_2_S, the response can be cell type and concentration-dependent [1, 2].

Although astrocytes contain the largest amounts of CBS in the brain, CBS has also been localized to other cell types in various animals and humans, including neurons and vascular structures [1, 2]. In our immunohistochemical analysis, the most intensive staining has been found in the astrocytes, while CBS in other structures only showed a faint signal. Importantly, a recent study has demonstrated the upregulation of CBS in pluripotent stem cell–derived human glutamatergic DS neurons, compared to healthy control neurons [54]. Our Western blotting analysis of synaptosomal preparations also indicates that CBS is also present in neurons in the current model and suggests that neurons are also subject to CBS upregulation in DS.

It should also be noted that there may be significant regional differences in the expression of CBS in the DS brain. In the rat DS model previously employed by our group, separate analysis of various brain regions was possible—as opposed to the current study where the entire hemisphere was analyzed as a whole. In the rat study, significant regional differences were found in the expression of several enzymes involved in the regulation of H_2_S levels (including CBS, 3-MST, and ETHE1). Interestingly, in the rat model, we have also noted—in a regionally heterogenous manner—the truncation of CBS to yield a lower molecular weight (45 kDa) form, which is constitutively active [14]. In contrast, in the current mouse model, only one isoform of CBS (the full-length, non-truncated form) was detected. Thus, there may be significant differences in heterogeneity in the localization of CBS overexpression in the DS brain, but, overall, the existing body of data—including, both Western blotting and proteomics data in the current report—support the conclusion that CBS levels are higher in DS brain than in control brain. This elevated CBS expression—together with higher levels of 3-MST, which, similar to CBS may show regional and species differences in its regulation—would be expected to contribute to increased H_2_S and polysulfide levels in the DS brain.

Similar to prior studies in human DS fibroblasts [37] and in the rat brain in the rat DS model [14], we have also observed an upregulation of a second H_2_S-producing enzyme, 3-MST. This enzyme is not encoded on chromosome 21 in humans nor on chromosome 17 in mice, and its upregulation is not due to a direct gene dosage effect. It is well documented that DS is associated with the dysregulation of many genes on all chromosomes [16, 17]. Thus, the observed effect is not surprising. Our working hypothesis is that the excess H_2_S may be involved in the transcriptional regulation of the *mpst* gene, because AOAA suppresses this upregulation in DS brains. Nevertheless, the mechanism of 3-MST upregulation in DS, as well as the functional role of H_2_S produced by this enzyme in DS, remains to be assessed in future studies.

It should be emphasized that not only the expression of several H_2_S-producing enzymes, but also the expression of several H_2_S-degrading enzymes was higher in the DS brain than in the wild-type control brains. In the current study, we have assessed 5 of these enzymes and found that the expression of TST, ETHE1, and SUOX was higher in DS brains than in control brains—perhaps as a reactive response to the elevated H_2_S generation in the tissues. When in a cell, both H_2_S production and H_2_S degradation are increased, the steady-state H_2_S levels are affected by both of these enzymes. This conclusion is supported by our in vitro biochemical measurements: when CBS or 3-MST-derived H_2_S was maximally stimulated in brain homogenates, the H_2_S-generating capacity increased by approximately twofold in our experiments. (In such experiments, the production of H_2_S overwhelms the capacity of the neutralizing enzymes.) However, when the “ambient” H_2_S levels were measured in the brain homogenates, the levels were only approximately 20% higher in DS brains than in wild-type controls.

With respect to the functional role of CBS in the current study—similar to multiple prior studies—we have used AOAA, a central nervous system-penetrable small molecule CBS inhibitor which is commonly used in H_2_S biology [19]. In line with the results obtained in a rat DS model (where CBS activity was inhibited by AOAA) [14] and in a mouse DS model (Dp1Yah, where CBS expression was reduced by genetic means or CBS activity was inhibited pharmacologically using disulfiram, a compound which has CBS inhibitory activity—in addition to its several additional actions) [12], in the current study, as well, neurocognitive function of the DS mice was apparent in the novel object recognition test and in the T-maze assay. AOAA improved neurocognitive function in both models. Supplemental experiments demonstrated that DS brains exhibited suppressed synaptosomal function, and AOAA-treated DS brains showed improved function. Moreover, the dysregulation of the UPR and autophagy in DS mouse brains was also partially reversed by AOAA.

An ideal study design would have been to conduct a dose–response study with AOAA and measure the degree of CBS inhibition in the brain, in order to select a dosing regimen, where the excess CBS activity seen in DS is reduced to healthy control levels, without inducing an overt inhibition of the activity of this enzyme below the physiological level. The reason for not striving for a more complete inhibition of CBS is the well-known neuroprotective function of endogenously produced H_2_S in the central nervous system [1, 2]. (Indeed, in our pilot experiments that preceded the current study, we found that higher doses of AOAA, i.e., 3 or 10 mg/kg/day, were less effective in improving neurological function in the current model and even trended to impair some neurobehavioral parameters in wild-type mice, Fig. S8.) Thus, we have selected a relatively low dose of the inhibitor for the current study in order to achieve a partial inhibition of this enzyme, but to avoid full inhibition. The fact that the degree of this inhibition has not been quantified remains a limitation of the current project.

AOAA is generally considered an irreversible inhibitor of CBS [19]. In a recent study, our group co-crystallized CBS with AOAA and has also demonstrated that there is some degree of reversibility of AOAA’s action, which is substrate-dependent [20]. Since AOAA’s inhibitory effect is PLP-dependent, the question arises as to whether, in the current study, off-target effects may have also contributed to its observed effect. There are many enzymes that are inhibited by AOAA (reviewed in 20). But, certainly, not all PLP enzymes are inhibited by this compound, and the potency of AOAA is also variable, depending on the enzyme in question. CBS is one of the enzymes which is inhibited by AOAA fairly potently, with an IC_50_ of approximately 8 µM. Because of the relatively low dose of the inhibitor used in the current study, and because none of the off-target enzymes that AOAA is known to inhibit are expected to regulate neurobehavioral responses or ER stress, we believe that the compound’s most likely mode of action, in the current study, is, indeed, inhibition of CBS. Nevertheless, for future studies, using genetic approaches and/or future, more potent or more selective CBS inhibitions should be conducted to follow up the current study.

In light of the upregulation of CBS in the DS brain, the observed *decrease* in protein persulfidation is unexpected. One possible hypothesis to explain these findings may be related to the increased consumption of H_2_S, because it is well documented that ROS generation is high in the DS brain [55]. Persulfides are much better scavengers of ROS than H_2_S, leading to the formation of S-sulfonates that are not detectable by the method used in the current study [47]. Additionally, high ROS production in DS brains could result in thiol hyperoxidation before persulfides could even be formed. This could explain the massive loss of protein persulfidation in male DS that generally showed much lower H_2_S levels than in female mice. Although the above list contains a list of highly diverse proteins, the directionally opposite persulfidation of ubiquitin-conjugating enzyme E2 J1 may be a potential contributor to some of the sex differences seen in some of the functional responses after CBS inhibition in DS brains. The biological significance of the class effect of altered persulfidation of two different members of the heterogeneous nuclear ribonucleoproteins class—as well as the functional role of a large number of proteins identified—remains to be further explored. Protein persulfidation is known to have an evolutionarily conserved effect [48]. If persulfidation is decreased in DS, this may predict accelerated aging or senescence of the central nervous system structures in DS. This hypothesis remains to be further investigated.

Previously, a neuron-centric approach—focusing on neurons and their connectivity—was primarily considered to explain the mechanisms involved in DS brain pathophysiology [3–6]. Several studies have drawn attention to alterations in neuronal survival and homeostasis, synaptogenesis, brain circuit, development, and neurodegeneration. However, recent studies propose to focus more on non-neuronal cells (glia), especially astrocytes in DS research. Astrocytes maintain crucial metabolic and neurotrophic support to neurons and regulate synapse plasticity, extracellular homeostasis, and neurovascular coupling [56], thus maintaining brain homeostasis. In this study, we showed that perturbations in astrocytes could affect the function of CNS and more importantly that targeting CBS/H_2_S pathways can improve the properties of astrocytes and reverse astrogliosis. Such an effect may provide functional benefit in the short term (for parameters assessed in the current study) but may also affect long-term processes in DS, such as chronic neurodegeneration. The potential role of the CBS/H_2_S pathway in the delayed pathophysiological events associated with DS (e.g., neurodegeneration, Alzheimer’s-like pathologies) remains to be investigated in the future. In this context, it is interesting to note that CBS expression in the brain declines as part of the physiological aging process [57]. If this is also the case in DS, then the pathophysiological role of the CBS/H_2_S pathway may be more relevant in the earlier part of the life of DS individuals than in later life stages. This, however, remains to be investigated and so is the question as to whether CBS—or, more broadly, the H_2_S system—plays a role in the pathogenesis of plaque formation and Alzheimer’s disease (AD)–like neuropathologies in DS.

An unexpected finding of the current study was the predominance of CBS expression and the preferential beneficial effect of CBS inhibition in female, rather than male DS mice. The topic of sex differences is now a high priority in neurology and translational neuroscience research. With respect to sex differences for major congenital heart defects, DS was observed to be more common in males [58, 59]. With respect to AD, women are disproportionately affected by AD: females account for around two-thirds of patients [60, 61]. Therefore, biological sex can influence key aspects of DS disease and Alzheimer’s disease, including molecular pathways, cognitive progression, and risk factor profiles. It was also reported that women with DS experience an earlier age of onset of menopause, marked by a drop in estrogen, than women without DS [62]. Clinically, females outperform males in verbal memory tests [63], but this better performance is lost at the dementia stage, possibly due to faster rates of cognitive deterioration in females with mild cognitive impairment compared with males [64]. Whether a similar heightened vulnerability to AD dementia in women holds true in the DS population remains to be investigated.

What mechanism, then, is responsible for the more pronounced upregulation of CBS in DS females than in DS males in the current model? Clearly, the effect appears to be DS-specific, as no sex differences in CBS expression were noted between wild-type control mice. The extra copy of the mouse chromosome 17 segment in the current animal model does not contain any proteins that are known to significantly regulate CBS expression (neither in a general sense nor in a sex-specific sense). There are, however, several studies that suggest that CBS expression may be under the control of various sex hormones. In uterine artery endothelial cells, CBS—both at the level of mRNA and at the level of protein—has been reported to increase after treatment with estradiol-17β or after treatment with estrogen receptor α or β agonists [65]. In addition, CBS has been shown to be upregulated during pregnancy in the uterine artery endothelial cells and smooth muscle cells [66]. It is possible that such transcriptional mechanisms may be involved in the current model, but the underlying mechanisms must be more complex than a simple upregulation, because we only observe the differences in DS females vs. males (and not in wild-type females vs. males).

In the metabolomic dataset, the effect of AOAA treatment was also investigated, and this appeared to affect—and, indeed, in many cases, partially or fully reverse—some of the metabolomic changes. It is hard to speculate if changes in any individual metabolite may have had any impact on the functional responses obtained in the DS mice. Nevertheless, some of the metabolomic changes are interesting and may deserve a brief discussion. There were several differences in the levels of many neurotransmitters between wild-type and DS mice, and in several cases, AOAA tended to reverse these changes in DS. Indeed, changes in monoamine turnover and associated functional activity have been shown to be altered in the DS brain; in some cases, these effects were previously found to be linked to changes in CBS and H_2_S homeostasis [67–69]. Serotonin and dopamine are derived from tryptophan and tyrosine, respectively, and have roles in mediating behavior. Serotonin, dopamine, and HVA (a metabolite of dopamine) are trending higher when comparing DS and wild-type control animals, while AOAA treatment of the DS animals leads to a reduction/lower trend in these metabolites. Monoamine neurotransmitters, such as serotonin and dopamine, act as developmental signals and regulators in the brain. The current findings may, in fact, implicate some role of the CBS/H_2_S pathway in mood, learning, and/or cognition responses in DS. Interestingly, in many cases—glycine, serine, and dopamine/homovanillate—the effects of AOAA in DS tended to show a predominant effect in male mice.

Polyamines are nitrogen-containing compounds found in many cell types and are derived from the amino acids arginine and ornithine. Their metabolism is thus connected with the urea cycle. Polyamines play a supportive role in cellular proliferation, and their levels can potentially affect proliferative capacity. These molecules are especially important in the brain as they are present at high concentrations and have been shown to be positively correlated with learning and memory in several studies [70]. The polyamines putrescine, N-acetylputrescine, and 5-methylthioadenosine (MTA) are trending lower in DS than in wild-type control animals. AOAA treatment in the DS animals results in an increase in putrescine and N-acetylputrescine, increasing their levels to that seen in wild-type animals, perhaps contributing to functional changes in learning and memory. The effect of AOAA in DS on polyamine metabolites did not show a sex difference.

Creatine phosphate is used for energy storage in the brain to regenerate adenosine 5′-triphosphate (ATP) from adenosine 5′-diphosphate (ADP) during increased energy needs. Creatine kinase catalyzes both the transfer of high-energy phosphate from ATP to creatine and the regeneration of ATP from creatine phosphate and ADP. Creatine can also spontaneously cyclize to creatinine. De novo biosynthesis of creatine involves methylation of guanidinoacetate via SAM, forming creatine and SAH. While creatine is unchanged, guanidinoacetate and creatinine are lower/trending lower when comparing the DS and the wild-type control animals, with AOAA treatment in DS animals increasing guanidinoacetate to the levels seen in the wild type. This difference in brain creatine metabolism between the DS and WT mice and the change due to AOAA treatment may be related to differences and changes in energy metabolism. As a whole, there are multiple urea cycle, creatine, and polyamine metabolites that are lower in DS control animals, compared to wild-type mice, with the levels of some of these metabolites being restored to that seen in the wild-type animals upon AOAA treatment of DS mice.

NADH plays a crucial role in oxidative phosphorylation as it serves as an initial donor of electrons in the electron transport chain. The NAD^+^/NADH redox state reflects the metabolic balance of the cell in the regeneration of ATP through oxidative phosphorylation in mitochondria and/or glycolysis in the cytosol [71]. In addition, NAD + can also be phosphorylated by NAD^+^ kinase to produce NADP^+^, which can be utilized in other redox reactions, such as glutathione reduction, as well as with fatty acid synthesis [72]. Here, the lower NAD^+^/NADH ratio in the DS animals may be indicative of impaired energy and redox homeostasis, a change that was ameliorated by AOAA treatment. The main hub of energy metabolism is often considered to be the mitochondrial tricarboxylic acid (TCA) cycle (aka Krebs cycle or Szent-Györgyi-Krebs cycle or citric acid cycle), which primarily links the catabolism of carbohydrates, lipids, and some amino acids to ATP production by supplying reducing equivalents through oxidative phosphorylation. Changes in TCA intermediates can reflect pathway activity (substrate influx and product outflux). Citrate, cis-aconitate, fumarate, and malate are lower in DS animals, compared to the wild type, perhaps indicative of decreased TCA activity. AOAA treatment did not appear to substantially impact the TCA cycle in the current experiments.

The endocannabinoid system plays a modulatory role in dopaminergic neurons. Endocannabinoids also activate G protein–coupled receptors such as CB1 in the brain and serve a unique and important function in the rapid retrograde regulation of synaptic strength [73]. Oleoyl ethanolamide has been reported to modulate a variety of biological processes such as neurotransmission, immunomodulation, and sphingolipid signaling. Additionally, oleoyl ethanolamide and palmitoyl ethanolamide possess anti-inflammatory properties, and N-palmitoyl serine has been shown to contribute to recovery from neurological damage [74, 75]. Oleoyl ethanolamine, palmitoyl ethanolamine, and N-palmitoyl serine are decreased in DS brains, with an increase seen in DS animals (preferentially in the male cohort) in response to AOAA treatment, bringing their levels to that seen in wild-type control animals.

In the above analyses, proteomic alterations, changes in the persulfidation status of proteins, and alterations in the brain levels of various metabolites were discussed separately. It would be ideal to have a method or tool to integrate these various alterations into a multi-omics network; however, we currently do not have a suitable method or technique to do so. Additionally—although much is being written about the promise of various multi-omics integration approaches [76–78]—such integrations are intrinsically limited by the facts of biology. For instance, mRNA levels do not always show a direct correlation with the levels of the protein it encodes (e.g., due to changes in protein translation, protein stability, and degradation); the levels of a protein do not show a direct correlation with its activity (e.g., due to intracellular translocation of the protein, various protein–protein interactions, co-factor availability, posttranslational modifications), and consequently, protein levels do not always induce the expected changes/shifts in the metabolites the protein is known to regulate. In addition, the functional relevance of the various protein persulfidations identified in the current project is not known at present; historically, persulfidation may increase or decrease protein activity and/or stability, but it may also be without functional consequence. Because of all of the above factors—as well as many other limitations [79] that are beyond the scope of the current article—we did not attempt to integrate the various omics measurements into a composite model in the current project.

As mentioned in the “Introduction” section, DS is associated with accelerated AD; in fact, clinical analysis of DS brains shows that amyloid plaques appear in the DS brain at a very early age, and essentially, all DS individuals who survive to middle age will develop the usual symptoms of AD [3–5]. The question as to whether the CBS/H_2_S pathway may contribute to the pathogenesis of DS-associated AD remains to be investigated in future studies. In this regard, on one hand, it should be pointed out that several investigators have proposed that AD has a pathogenetic basis in mitochondrial dysfunction and bioenergetic deficiency [80–82]; in this regard, a H_2_S-mediated inhibition of mitochondrial ATP generation—alone or in combination with various pathophysiological processes in DS—may serve as a candidate for the pathogenesis of DS-associated AD. On the other hand, however, recent studies demonstrate that the expression of CBS in the brain decreases as part of the physiological aging process [83]; if this is also the case for CBS expression in DS (which remains to be investigated), then it is possible that the role of the CBS/H_2_S pathway in DS is more relevant in the earlier, rather than the later years of the condition.

## Conclusions

In summary, in the current study, using the Dp(17)3Yey/ + mice—a model of DS which involves an extra copy of the mouse gene that encodes for *cbs*—we have demonstrated the functional role of the CBS/H_2_S pathway in the pathogenesis of neurobehavioral dysfunction, with a preferential role in female DS mice. The CBS/H_2_S pathway was found to be responsible for the impairment of recognition memory and impaired spatial learning. The underlying mechanisms likely involve cellular bioenergetic dysfunction, dysregulation of ER stress, and the promotion of reactive astrogliosis. These functional differences are likely driven by proteomic and metabolomic alterations, including changes in the persulfidation of various proteins that regulate the above processes. Taken together, the data lend further support to the Kamoun hypothesis, i.e., that excess H_2_S in DS contributes to the pathophysiological neurological events and that pharmacological inhibition of CBS may be of potential future therapeutic utility in this condition.

### Supplementary Information

Below is the link to the electronic supplementary material.

Supplementary file1 (XLSX 2600 KB)

Supplementary file2 (XLSX 84806 KB)

Supplementary file3 (XLSX 1009 KB)

Supplementary file4 (DOCX 40 KB)

Supplementary file5 (DOCX 36 KB)

Supplementary file6 (DOCX 45 KB)

Supplementary file7 (DOCX 36 KB)

Supplementary file8 (DOCX 38 KB)

Supplementary file9 (XLSX 42 KB)

Supplementary file10 (JPG 603 KB)

Supplementary file11 (JPG 482 KB)

Supplementary file12 (JPG 325 KB)

Supplementary file13 (JPG 1429 KB)

Supplementary file14 (JPG 440 KB)

Supplementary file15 (JPG 466 KB)

Supplementary file16 (JPG 1060 KB)

Supplementary file17 (JPG 867 KB)

Supplementary file18 (DOCX 14 KB)

Supplementary file19 (XLSX 37021 KB)

## Data Availability

The authors declare that the data supporting the findings of this study are available within the paper and its Supplementary Information files. Should any raw data files be needed in another format they are available from the corresponding author (csaba.szabo@unifr.ch) upon reasonable request.

## Abbreviations

%RI: Recognition Index
3-MST: 3-Mercaptopyruvate sulfurtransferase
AD: Alzheimer’s disease
AOAA: Aminooxyacetic acid
ATF6: Activating transcription factor 6
ATF6f: Active, 50-kDa fragment of activating transcription factor 6
Atg3: Autophagy-related 3
Atg7: Autophagy-related 7
ATP: Adenosine triphosphate
AzMC: 7-Azido-4-methylcoumarin
BiP: Immunoglobulin heavy-chain binding protein
CARS2: Cysteinyl-tRNA synthetase 2
CBS: Cystathionine β-synthase
Chop: C/EBP homologous protein
CSE: Cystathionine γ-lyase
Cys: Cysteine
DS: Down syndrome
EEG: Electroencephalogram
ER: Endoplasmic reticulum
ERO1-Lα: ERO1-like protein α
ETHE1: Ethylmalonic encephalopathy 1 protein
GFAP: Glial fibrillary acidic protein
GSH: Glutathione, reduced
GSSH: Glutathione, oxidized
H_2_S: Hydrogen sulfide
H_2_S_2_: Polysulfide
IRE1α: Inositol-requiring transmembrane kinase/endoribonuclease 1α
KEGG: Kyoto Encyclopedia of Genes and Genomes
LC3B: Microtubule-associated protein 1A/1B-light chain 3B
LGUL: Lactoylglutathione lyase
LIMC1: Calponin homology domain—containing protein 1
MUTA: Methylmalonyl-CoA mutase
OXPHOS: Oxidative phosphorylation
PDI: Protein disulfide isomerase
PERK: Protein kinase–like endoplasmic reticulum kinase
PITM2: Membrane-associated phosphatidylinositol transfer protein 2
PLP: Pyridoxal 5′-phosphate
PSD95: Post-synaptic density protein of 95 kDa
SAM: S-adenosyl-L-methionine
SEM: Standard error of the mean
SOD1: Superoxide dismutase 1
SQR: Sulfur dioxygenase
SUOX: Sulfide quinone oxidoreductase
SV: Synaptic vesicles
Sys: Synaptophysin
TCA: Tricarboxylic acid
TFA: Trifluoroacetic acid
TST: Thiosulfate sulfurtransferase
UPR: Unfolded protein response
WT: Wild type
