# Supplementary material for: Neurobehavioral dysfunction in a mouse model of Down syndrome: upregulation of cystathionine β-synthase, H2S overproduction, altered protein persulfidation, synaptic dysfunction, endoplasmic reticulum stress, and autophagy
Source: GeroScience. 2024 Apr 1;46(5):4275–314. doi: 10.1007/s11357-024-01146-8 (PMC11336008; doi:10.1007/s11357-024-01146-8)
Supplement: Supplementary file 4 — Supplementary file4 (DOCX 40 KB) [file 11357_2024_1146_MOESM4_ESM.docx]

**Table S4.** Relative ratios of amino acid sub pathway metabolites between DS mouse brain vs. wild-type mouse brain and AOAA-treated DS mouse brain vs. DS mouse brain.^1^

| **Subpathway** | **Analyte** | **DS/WT** | **DS+AOAA/DS** |
| --- | --- | --- | --- |
| **Glycine,**  **Serine**  **and**  **Threonine**  **Metabolism** | glycine | 0.53* | 1.39 |
|  | sarcosine | 0.97 | 1.09 |
|  | dimethylglycine | 0.75 | 1.05 |
|  | betaine | 0.79^ | 1.03 |
|  | serine | 1.28^ | 0.88 |
|  | N-acetylserine | 0.98 | 1.03 |
|  | 2-methylserine | 0.76* | 1.05 |
|  | threonine | 0.94 | 1.05 |
|  | N-acetylthreonine | 1.07 | 1.04 |
|  | allo-threonine | 1.54 | 0.75 |
|  | homoserine | 0.76 | 1.00 |
| **Histidine**  **Metabolism** | histidine | 1.06 | 1.06 |
|  | 1-methylhistidine | 1.02 | 0.71 |
|  | 3-methylhistidine | 1.03 | 0.97 |
|  | N-acetylhistidine | 1.01 | 0.98 |
|  | N-acetyl-1-methylhistidine | 1.00 | 0.78 |
|  | trans-urocanate | 1.82 | 0.75 |
|  | imidazole propionate | 0.76^ | 0.69^ |
|  | formiminoglutamate | 0.73^ | 1.08 |
|  | imidazole lactate | 1.20^ | 0.83^ |
|  | carnosine | 1.20 | 1.47 |
|  | homocarnosine | 0.61* | 1.77* |
|  | N-acetylcarnosine | 1.84 | 1.21 |
|  | anserine | 0.96 | 1.27 |
|  | histamine | 1.25 | 0.95 |
|  | 1-methylhistamine | 1.13 | 0.85 |
|  | 1-methyl-4-imidazoleacetate | 0.98 | 0.88 |
| **Tyrosine**  **Metabolism** | tyrosine | 1.25 | 0.88 |
|  | N-acetyltyrosine | 1.38 | 0.86 |
|  | 3-(4-hydroxyphenyl)lactate | 1.53* | 0.82 |
|  | phenol sulfate | 2.71* | 0.77 |
|  | dopamine | 2.61* | 0.53 |
|  | 3-methoxytyramine | 2.09^ | 0.66 |
|  | homovanillate | 1.93 | 0.56* |
|  | O-methyltyrosine | 1.25 | 0.90 |
|  | N-formylphenylalanine | 0.88 | 1.23 |
| **Urea cycle.**  **Arginine**  **and**  **Proline**  **Metabolism** | arginine | 0.88* | 1.13* |
|  | argininosuccinate | 0.62* | 1.37^ |
|  | urea | 0.95 | 1.13 |
|  | ornithine | 1.02 | 1.12 |
|  | 3-amino-2-piperidone | 1.01 | 0.97 |
|  | 2-oxoarginine | 0.94 | 1.00 |
|  | citrulline | 0.80 | 1.20 |
|  | homoarginine | 0.85 | 2.09^ |
|  | homocitrulline | 1.12 | 0.92 |
|  | proline | 1.09 | 1.01 |
|  | dimethylarginine (SDMA + ADMA) | 1.02 | 1.02 |
|  | N-acetylarginine | 1.16 | 1.00 |
|  | N-delta-acetylornithine | 1.00 | 1.05 |
|  | trans-4-hydroxyproline | 0.96 | 0.98 |
|  | pro-hydroxy-pro | 0.87 | 1.32 |
|  | N,N,N-trimethyl-alanylproline betaine | 1.04 | 1.08 |
|  | argininate | 0.90 | 0.92 |
| **Polyamine**  **Metabolism** | putrescine | 0.86^ | 1.31* |
|  | N-acetylputrescine | 0.87 | 1.48* |
|  | N-acetyl-isoputreanine | 0.62 | 0.94 |
|  | spermidine | 0.48 | 1.07 |
|  | (N1 + N8)-acetylspermidine | 0.84 | 1.22 |
|  | spermine | 0.52 | 1.03 |
|  | 5-methylthioadenosine | 0.92 | 1.06 |
|  | 4-acetamidobutanoate | 1.31 | 1.00 |

^1^ Data are expressed as mean of n=6 per group; *p<0.05; ^p<0.1
