# Supplementary material for: Neurobehavioral dysfunction in a mouse model of Down syndrome: upregulation of cystathionine β-synthase, H2S overproduction, altered protein persulfidation, synaptic dysfunction, endoplasmic reticulum stress, and autophagy
Source: GeroScience. 2024 Apr 1;46(5):4275–314. doi: 10.1007/s11357-024-01146-8 (PMC11336008; doi:10.1007/s11357-024-01146-8)
Supplement: Supplementary file 5 — Supplementary file5 (DOCX 36 KB) [file 11357_2024_1146_MOESM5_ESM.docx]

**Table S5.** Relative ratios of carbohydrate metabolism and the ‘energy sub pathway’ metabolites between DS mouse brain vs. wild-type mouse brain and AOAA-treated DS mouse brain vs. DS mouse brain.^1^

| **Subpathway** | **Analyte** | **DS/WT** | **DS+AOAA/DS** |
| --- | --- | --- | --- |
| **Glycolysis, Gluconeogenesis**  **and**  **Pyruvate**  **Metabolism** | 1,5-anhydroglucitol | 1.09 | 1.07 |
|  | glucose | 0.42* | 1.16 |
|  | glucose 6-phosphate | 0.97 | 0.98 |
|  | fructose/glucose 1,6-diphosphate**^2^** | 0.72 | 1.12 |
|  | 2-phosphoglycerate | 1.25 | 0.91 |
|  | 3-phosphoglycerate | 1.39 | 0.77 |
|  | phosphoenolpyruvate | 1.51 | 0.76 |
|  | pyruvate | 1.13 | 0.94 |
|  | lactate | 0.91^ | 0.99 |
|  | glycerate | 1.08 | 0.97 |
| **Pentose**  **Phosphate**  **Pathway** | 6-phosphogluconate | 1.26 | 0.55 |
|  | ribose 5-phosphate | 0.89 | 1.29 |
|  | ribose 1-phosphate | 0.97 | 0.97 |
|  | sedoheptulose-7-phosphate | 0.63* | 1.13 |
| **TCA**  **Cycle** | citrate | 0.81* | 1.06 |
|  | aconitate [cis or trans] | 0.74* | 1.10 |
|  | alpha-ketoglutarate | 1.08 | 0.97 |
|  | succinylcarnitine | 0.56* | 1.34 |
|  | succinate | 1.23 | 1.10 |
|  | fumarate | 0.82* | 1.13 |
|  | malate | 0.83* | 1.04 |
|  | 2-methylcitrate/homocitrate | 1.06 | 0.93 |

^1^ Data are expressed as mean of n=6 per group; *p<0.05; ^p<0.1; ^2^ also includes myo-inositol diphosphates
