# Supplementary material for: Neurobehavioral dysfunction in a mouse model of Down syndrome: upregulation of cystathionine β-synthase, H2S overproduction, altered protein persulfidation, synaptic dysfunction, endoplasmic reticulum stress, and autophagy
Source: GeroScience. 2024 Apr 1;46(5):4275–314. doi: 10.1007/s11357-024-01146-8 (PMC11336008; doi:10.1007/s11357-024-01146-8)
Supplement: Supplementary file 6 — Supplementary file6 (DOCX 45 KB) [file 11357_2024_1146_MOESM6_ESM.docx]

**Table S6.** Relative ratios of the lipid sub pathway metabolites between DS mouse brain vs. wild-type mouse brain and AOAA-treated DS mouse brain vs. DS mouse brain^1^

| **Subpathway** | **Analyte** | **DS/WT** | **DS+AOAA/DS** |
| --- | --- | --- | --- |
| **Long Chain Monosaturated**  **Fatty Acid** | palmitoleate (16:1n7) | 0.92 | 1.39^ |
|  | 10-heptadecenoate (17:1n7) | 0.82 | 1.53* |
|  | oleate/vaccenate (18:1) | 0.86 | 1.46^ |
|  | 10-nonadecenoate (19:1n9) | 0.83 | 1.51 |
|  | **eicosenoate** (20:1) | 0.63* | 1.67* |
|  | **erucate** (22:1n9) | 0.64* | 1.50^ |
| **Long Chain Polyunsaturated**  **Fatty Acid**  **(n3 and n6)** | tetradecadienoate (14:2) | 0.89 | 1.15 |
|  | **eicosapentaenoate (EPA; 20:5n3)** | 0.63^ | 1.97* |
|  | docosapentaenoate (n3 DPA; 22:5n3) | 0.73 | 1.53^ |
|  | docosahexaenoate (DHA; 22:6n3) | 0.85 | 1.51^ |
|  | **nisinate (24:6n3)** | 0.58* | 1.85* |
|  | hexadecadienoate (16:2n6) | 0.54* | 1.36 |
|  | linoleate (18:2n6) | 0.87 | 1.30 |
|  | linolenate [alpha or gamma; (18:3n3 or 6)] | 0.95 | 1.24 |
|  | dihomo-linoleate (20:2n6) | 0.82 | 1.56* |
|  | dihomo-linolenate (20:3n3 or n6) | 0.87 | 1.24 |
|  | arachidonate (20:4n6) | 1.09 | 1.34 |
|  | docosatrienoate (22:3n6) | 0.71 | 1.71* |
|  | adrenate (22:4n6) | 0.90 | 1.40 |
|  | docosapentaenoate (n6 DPA; 22:5n6) | 1.02 | 1.43 |
|  | docosadienoate (22:2n6) | 0.58 | 1.71* |
| **Fatty Acid**  **Metabolism**  **(Acyl Carnitine,**  **Long Chain Saturated)** | myristoylcarnitine (C14) | 0.97 | 0.82 |
|  | palmitoylcarnitine (C16) | 0.82 | 0.94 |
|  | margaroylcarnitine (C17) | 0.67 | 1.19 |
|  | stearoylcarnitine (C18) | 0.60^ | 1.42 |
|  | **arachidoylcarnitine (C20)** | 0.60^ | 1.68^ |
|  | behenoylcarnitine (C22) | 0.53* | 1.57 |
|  | lignoceroylcarnitine (C24) | 0.94 | 1.03 |

| **Fatty Acid, Monohydroxy** | 2-hydroxyheptanoate | 0.75 | 2.32* |
| --- | --- | --- | --- |
|  | 2-hydroxyoctanoate | 1.01 | 0.98 |
|  | 2-hydroxydecanoate | 1.14 | 0.96 |
|  | 2-hydroxyoleate | 1.51 | 1.33 |
|  | 2-hydroxystearate | 0.86 | 1.42* |
|  | 2-hydroxybehenate | 0.52* | 1.55 |
|  | 2-hydroxynervonate | 0.44* | 1.57 |
|  | 3-hydroxyhexanoate | 0.92 | 0.92 |
|  | 3-hydroxyoctanoate | 0.87 | 1.05 |
|  | 3-hydroxydecanoate | 0.80 | 1.12 |
|  | 3-hydroxylaurate | 0.68^ | 1.37 |
|  | 3-hydroxystearate | 0.79 | 1.35* |
|  | 3-hydroxyoleate | 0.75^ | 1.70* |
|  | 13-HODE + 9-HODE | 0.71^ | 1.37 |
| **Endocannabinoid** | **oleoyl ethanolamide** | 0.71* | 1.70* |
|  | **palmitoyl ethanolamide** | 0.69* | 1.75* |
|  | stearoyl ethanolamide | 0.71* | 1.23 |
|  | docosahexaenoyl ethanolamide | 0.69 | 1.12 |
|  | arachidonoyl ethanolamide | 0.84 | 1.17 |
|  | N-arachidonoyltaurine | 1.14 | 1.55 |
|  | N-oleoyltaurine | 0.99 | 1.53 |
|  | N-stearoyltaurine | 1.08 | 1.29 |
|  | N-palmitoyltaurine | 1.09 | 1.30 |
|  | linoleoyl ethanolamide | 0.66 | 1.32 |
|  | arachidoyl ethanolamide (20:0)* | 0.38 | 1.77 |
|  | lignoceroyl ethanolamide (24:0)* | 0.79* | 1.00 |
|  | nervonoyl ethanolamide (24:1)* | 0.34* | 1.67 |
|  | palmitoleoyl ethanolamide* | 0.76 | 1.03 |
|  | N-oleoylserine | 0.64 | 1.49 |
|  | N-stearoylserine* | 0.91 | 1.11 |
|  | **N-palmitoylserine** | 0.70* | 1.40* |

| **Lysophospholipid** | 1-palmitoyl-GPC (16:0) | 0.94 | 1.05 |
| --- | --- | --- | --- |
|  | 2-palmitoyl-GPC (16:0) | 0.88 | 1.47 |
|  | 1-palmitoleoyl-GPC (16:1) | 1.08 | 1.14 |
|  | 1-stearoyl-GPC (18:0) | 0.86* | 1.03 |
|  | 1-oleoyl-GPC (18:1) | 0.81 | 1.31 |
|  | 1-linoleoyl-GPC (18:2) | 0.94 | 1.17 |
|  | 1-arachidonoyl-GPC (20:4n6) | 1.00 | 1.49 |
|  | 1-lignoceroyl-GPC (24:0) | 0.35^ | 1.91 |
|  | 1-palmitoyl-GPE (16:0) | 0.93 | 1.03 |
|  | 1-stearoyl-GPE (18:0) | 0.98 | 0.94 |
|  | 2-stearoyl-GPE (18:0) | 0.99 | 1.10 |
|  | **1-oleoyl-GPE (18:1)** | 0.62* | 1.45* |
|  | **1-linoleoyl-GPE (18:2)** | 0.75^ | 1.34^ |
|  | 1-arachidonoyl-GPE (20:4n6) | 1.10 | 1.24 |
|  | 1-palmitoyl-GPS (16:0) | 0.82 | 1.84 |
|  | 1-stearoyl-GPS (18:0) | 1.02 | 0.93 |
|  | **1-oleoyl-GPS (18:1)** | 0.52* | 2.06* |
|  | **1-palmitoyl-GPG (16:0)** | 0.64* | 1.70* |
|  | **1-stearoyl-GPG (18:0)** | 0.66* | 1.48* |
|  | **1-oleoyl-GPG (18:1)** | 0.58* | 1.72* |
|  | 1-linoleoyl-GPG (18:2) | 0.57 | 1.88 |
|  | 1-palmitoyl-GPI (16:0) | 0.79 | 1.51 |
|  | 1-stearoyl-GPI (18:0) | 0.90 | 1.33 |
|  | 1-oleoyl-GPI (18:1) | 0.76 | 1.48^ |
|  | 1-linoleoyl-GPI (18:2) | 0.67 | 1.71^ |
|  | 1-arachidonoyl-GPI (20:4) | 0.96 | 1.46 |

^1^ Data are expressed as mean of n=6 per group; *p<0.05; ^p<0.1
