# Supplementary material for: Neurobehavioral dysfunction in a mouse model of Down syndrome: upregulation of cystathionine β-synthase, H2S overproduction, altered protein persulfidation, synaptic dysfunction, endoplasmic reticulum stress, and autophagy
Source: GeroScience. 2024 Apr 1;46(5):4275–314. doi: 10.1007/s11357-024-01146-8 (PMC11336008; doi:10.1007/s11357-024-01146-8)
Supplement: Supplementary file 7 — Supplementary file7 (DOCX 36 KB) [file 11357_2024_1146_MOESM7_ESM.docx]

**Table S7.** Relative ratios of endocannabinoid pathway metabolites between DS mouse brain vs. wild-type mouse brain and AOAA-treated DS mouse brain vs. DS mouse brain.^1^

| **Subpathway** | **Analyte** | **DS/WT** | **DS+AOAA/DS** |
| --- | --- | --- | --- |
| **Endocannabinoid pathway** | oleoyl ethanolamide | 0.71* | 1.70* |
|  | palmitoyl ethanolamide | 0.69* | 1.75* |
|  | stearoyl ethanolamide | 0.71* | 1.23 |
|  | docosahexaenoyl ethanolamide | 0.69 | 1.12 |
|  | arachidonoyl ethanolamide | 0.84 | 1.17 |
|  | N-arachidonoyltaurine | 1.14 | 1.55 |
|  | N-oleoyltaurine | 0.99 | 1.53 |
|  | N-stearoyltaurine | 1.08 | 1.29 |
|  | N-palmitoyltaurine | 1.09 | 1.30 |
|  | linoleoyl ethanolamide | 0.66 | 1.32 |
|  | arachidoyl ethanolamide | 0.38 | 1.77 |
|  | lignoceroyl ethanolamide | 0.79* | 1.00 |
|  | nervonoyl ethanolamide | 0.34* | 1.67 |
|  | palmitoleoyl ethanolamide | 0.76 | 1.03 |
|  | N-oleoylserine | 0.64 | 1.49 |
|  | N-stearoylserine | 0.91 | 1.11 |
|  | N-palmitoylserine | 0.70* | 1.40* |

^1^ Data are expressed as mean of n=6 per group; *p<0.05; ^p<0.1
