# Supplementary material for: Neurobehavioral dysfunction in a mouse model of Down syndrome: upregulation of cystathionine β-synthase, H2S overproduction, altered protein persulfidation, synaptic dysfunction, endoplasmic reticulum stress, and autophagy
Source: GeroScience. 2024 Apr 1;46(5):4275–314. doi: 10.1007/s11357-024-01146-8 (PMC11336008; doi:10.1007/s11357-024-01146-8)
Supplement: Supplementary file 8 — Supplementary file8 (DOCX 38 KB) [file 11357_2024_1146_MOESM8_ESM.docx]

**Table S8.** Relative ratios of nucleotide sub pathway metabolites between DS mouse brain vs. wild-type mouse brain and AOAA-treated DS mouse brain vs. DS mouse brain.^1^

| **Subpathway** | **Analyte** | **DS/WT** | **DS+AOAA/DS** |
| --- | --- | --- | --- |
| **Purine**  **Metabolism,**  **Adenine containing** | adenosine 5'-diphosphate (ADP) | 1.29^ | 0.79 |
|  | adenosine 5'-monophosphate (AMP) | 1.14^ | 0.95 |
|  | adenosine 2'-monophosphate (2’AMP) | 0.60* | 1.39 |
|  | adenosine 3',5'-cyclic monophosphate (cAMP) | 1.11 | 1.00 |
|  | adenosine 3',5'-diphosphate | 0.90 | 1.06 |
|  | adenylosuccinate | 0.77* | 1.01 |
|  | adenosine | 1.17 | 0.89 |
|  | adenine | 0.90 | 1.15 |
|  | N1-methyladenosine | 1.09 | 0.87 |
|  | N6-carbamoylthreonyladenosine | 0.92 | 1.23 |
|  | 2'-deoxyadenosine 5'-monophosphate | 1.14 | 0.90 |
|  | 2'-deoxyadenosine | 1.26 | 0.87 |
|  | N6-succinyladenosine | 0.63* | 1.35* |
| **Pyrimidine**  **Metabolism,**  **Uracil**  **containing** | uridine 5'-diphosphate (UDP) | 2.05* | 0.44^ |
|  | uridine 5'-monophosphate (UMP) | 1.14 | 0.88 |
|  | uridine | 0.95 | 1.06 |
|  | uracil | 1.11 | 0.97 |
|  | pseudouridine | 1.07 | 0.99 |
|  | 5,6-dihydrouridine | 1.15 | 0.97 |
|  | 2'-O-methyluridine | 1.16 | 0.88 |
|  | 5-methyluridine (ribothymidine) | 0.64^ | 1.49 |
|  | 5,6-dihydrouracil | 1.42^ | 0.81 |
|  | 2'-deoxyuridine | 1.03 | 1.09 |
|  | 3-ureidopropionate | 0.97 | 1.00 |
|  | beta-alanine | 1.00 | 1.03 |
|  | N-acetyl-beta-alanine | 0.92 | 1.12 |
|  | 3-(3-amino-3-carboxypropyl) uridine* | 1.11 | 0.99 |
| **Nicotinate and Nicotinamide Metabolism** | nicotinamide | 0.92 | 0.92 |
|  | nicotinamide ribonucleotide (NMN) | 1.31 | 1.00 |
|  | nicotinamide riboside | 1.11 | 1.00 |
|  | NAD^+^ | 2.64* | 0.90 |
|  | NADH | 4.05* | 0.56* |
|  | 1-methylnicotinamide | 1.06 | 0.90 |
|  | trigonelline (N'-methylnicotinate) | 0.61* | 1.14 |
|  | N1-methyl-2-pyridone-5-carboxamide | 0.97 | 0.98 |
|  | N1-methyl-4-pyridone-3-carboxamide | 1.14 | 0.92 |
|  | adenosine 5'-diphosphoribose (ADP-ribose) | 0.70 | 1.00 |

^1^ Data are expressed as mean of n=6 per group; *p<0.05; ^p<0.1
