# Supplementary figures and images for: Neurobehavioral dysfunction in a mouse model of Down syndrome: upregulation of cystathionine β-synthase, H2S overproduction, altered protein persulfidation, synaptic dysfunction, endoplasmic reticulum stress, and autophagy
Source: GeroScience. 2024 Apr 1;46(5):4275–314. doi: 10.1007/s11357-024-01146-8 (PMC11336008; doi:10.1007/s11357-024-01146-8)

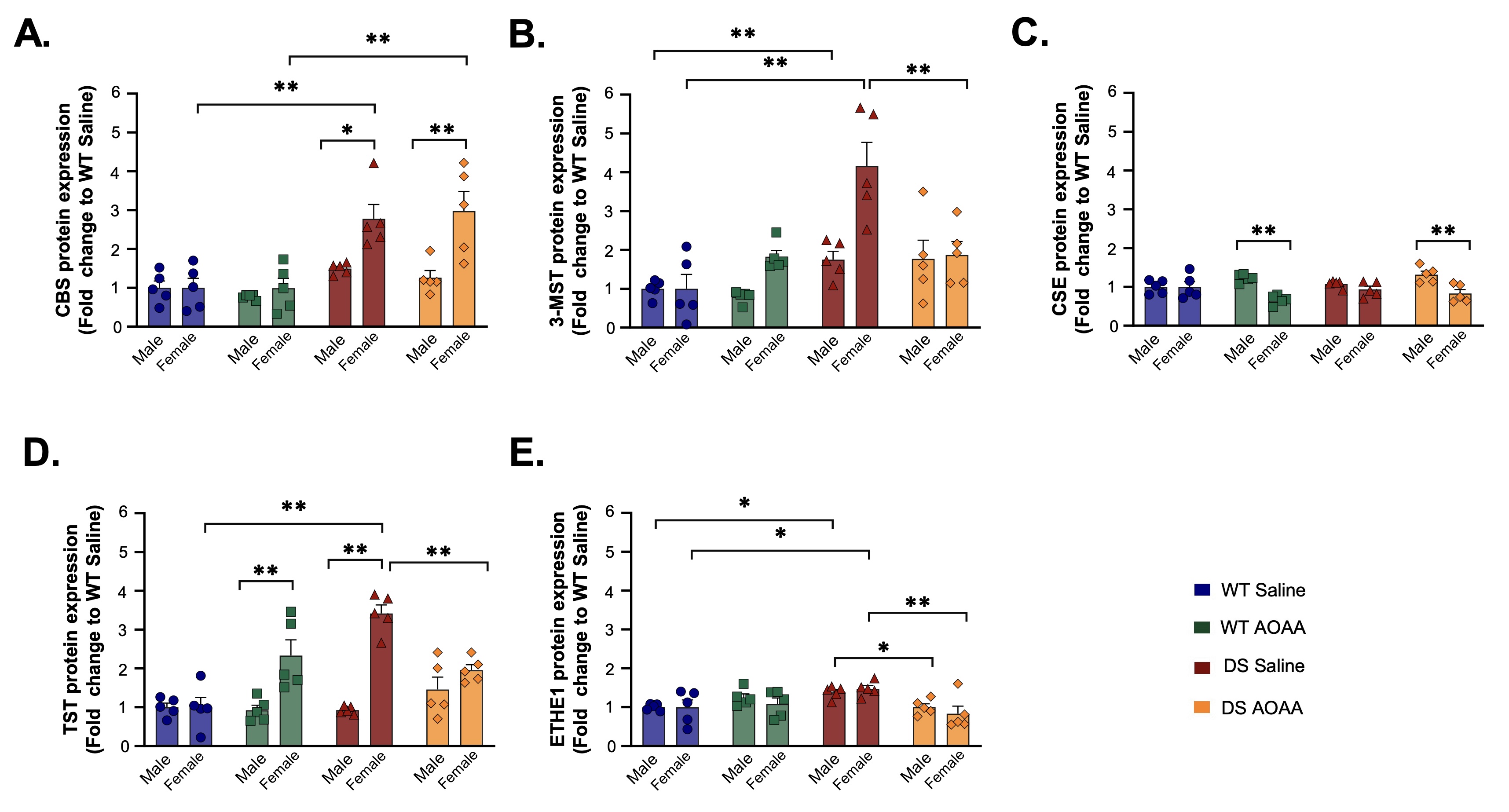

Supplement: Supplementary file 10 — Supplementary file10 (JPG 603 KB) [file 11357_2024_1146_MOESM10_ESM.jpg]

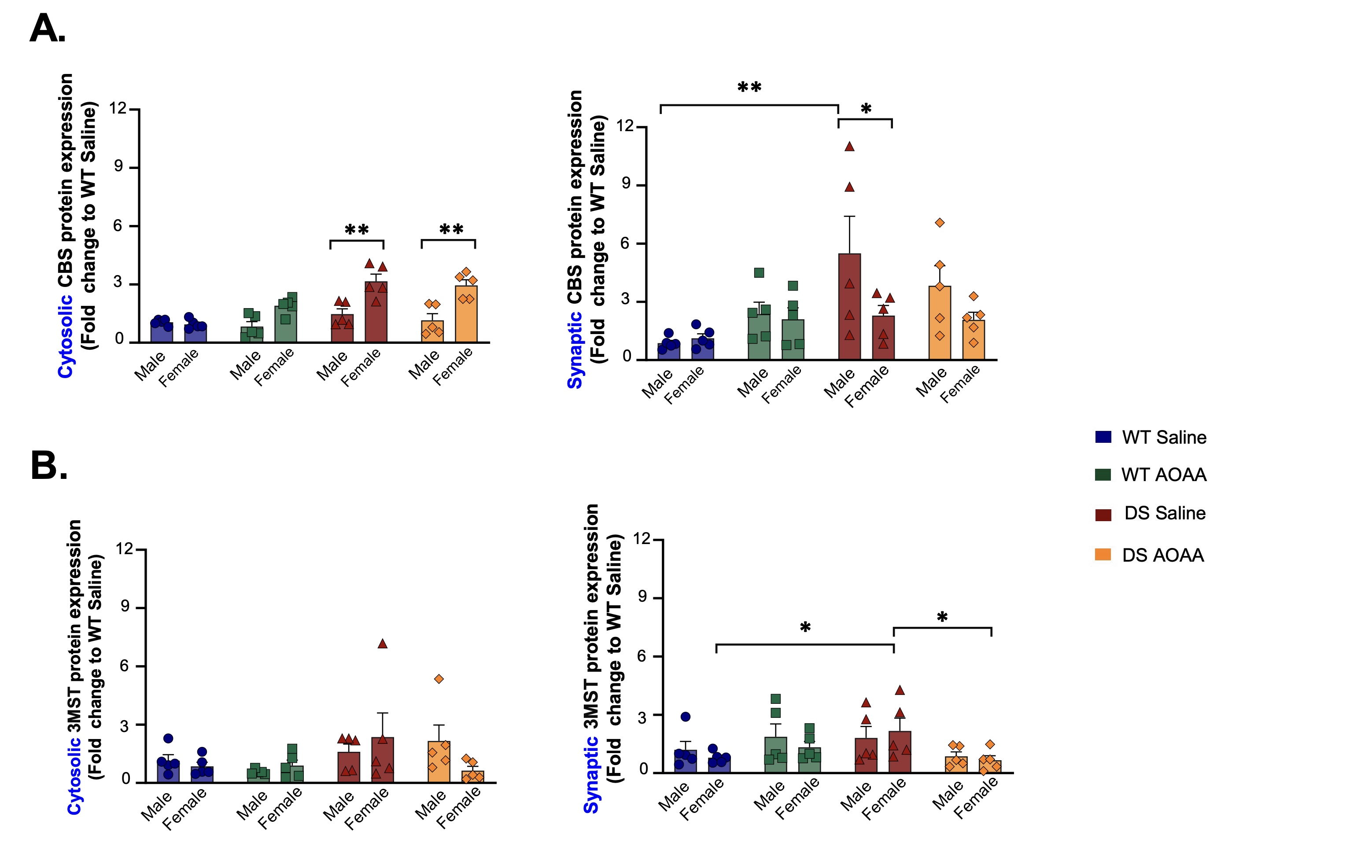

Supplement: Supplementary file 11 — Supplementary file11 (JPG 482 KB) [file 11357_2024_1146_MOESM11_ESM.jpg]

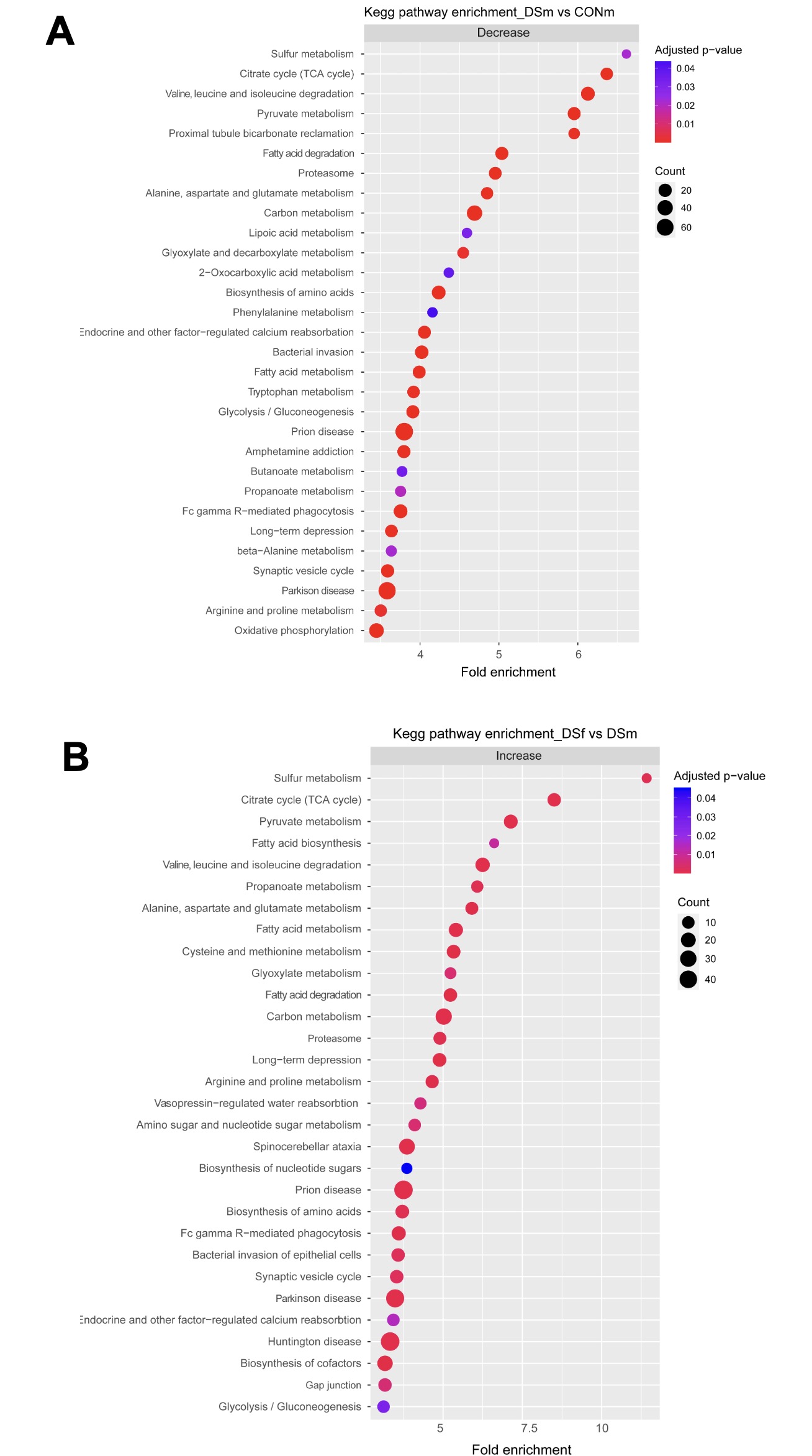

Supplement: Supplementary file 12 — Supplementary file12 (JPG 325 KB) [file 11357_2024_1146_MOESM12_ESM.jpg]

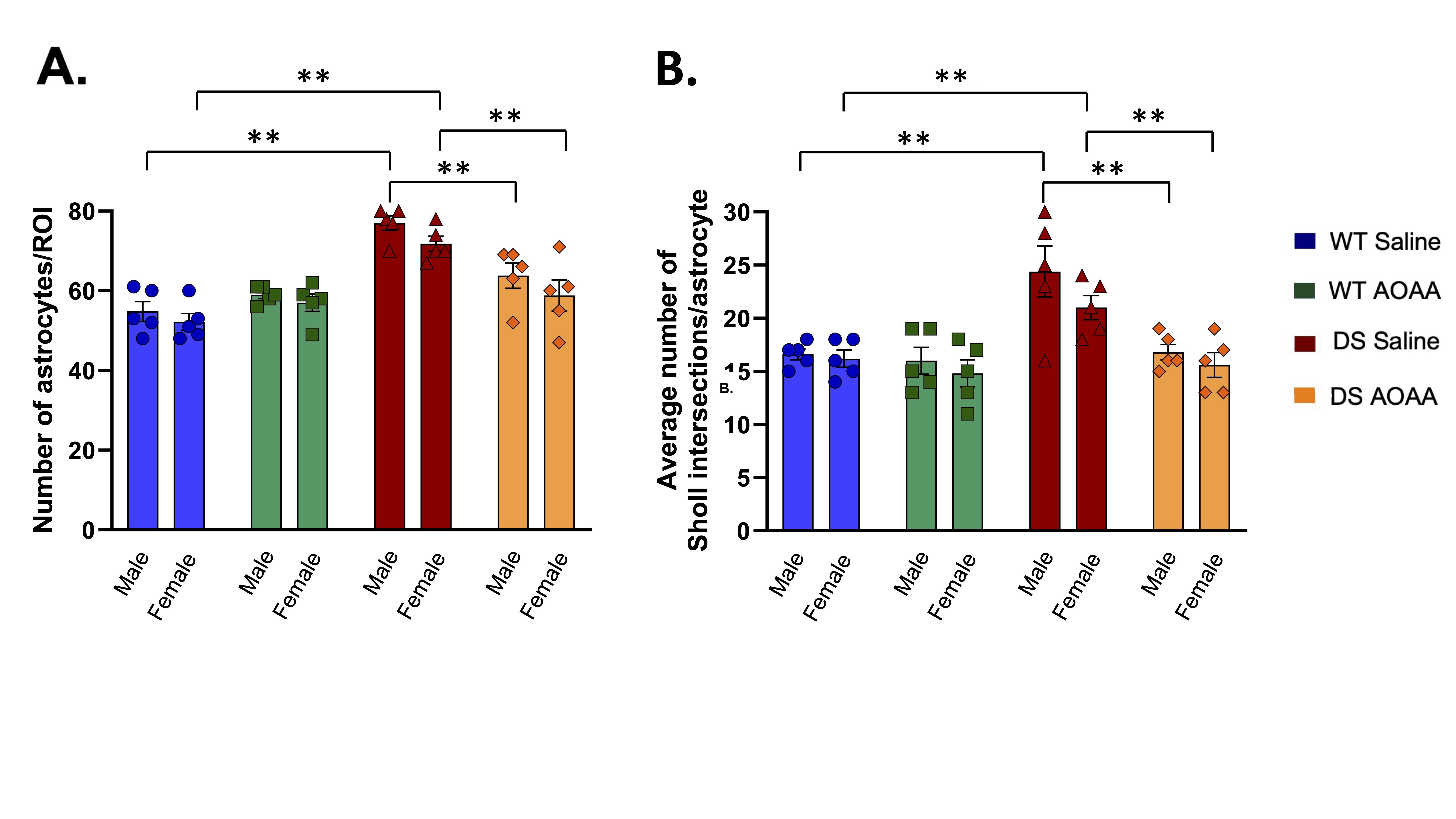

Supplement: Supplementary file 13 — Supplementary file13 (JPG 1429 KB) [file 11357_2024_1146_MOESM13_ESM.jpg]

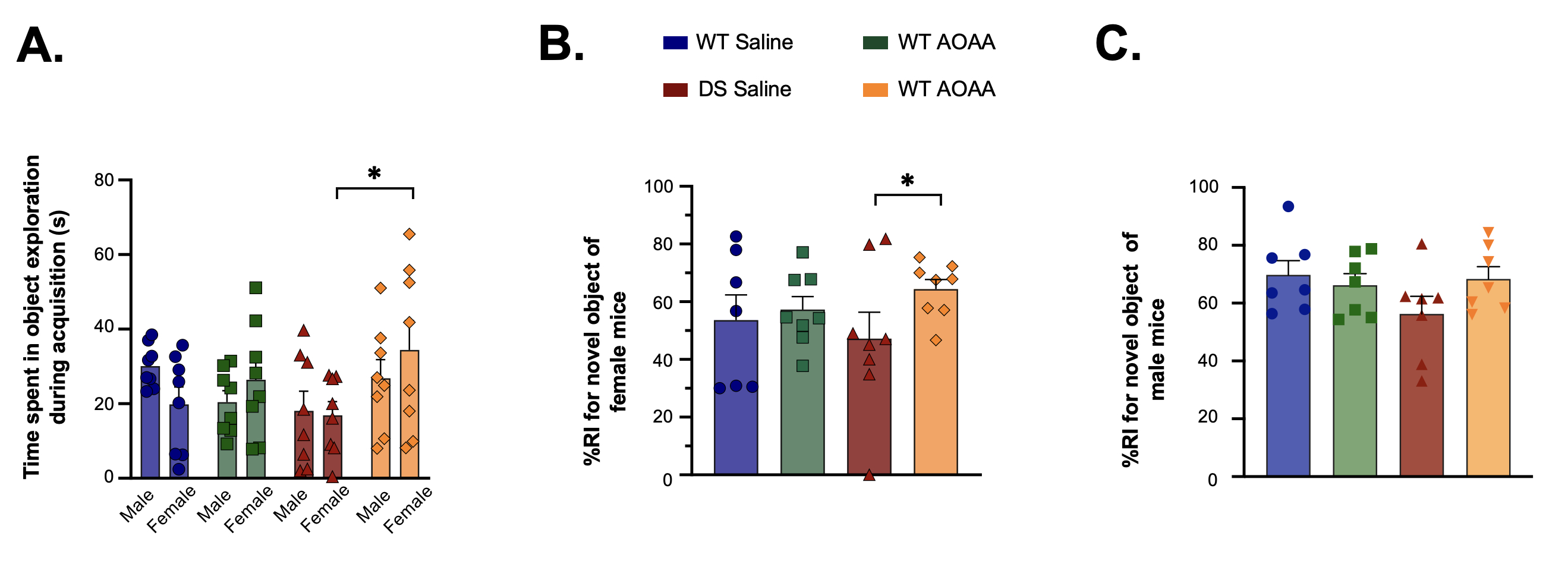

Supplement: Supplementary file 14 — Supplementary file14 (JPG 440 KB) [file 11357_2024_1146_MOESM14_ESM.jpg]

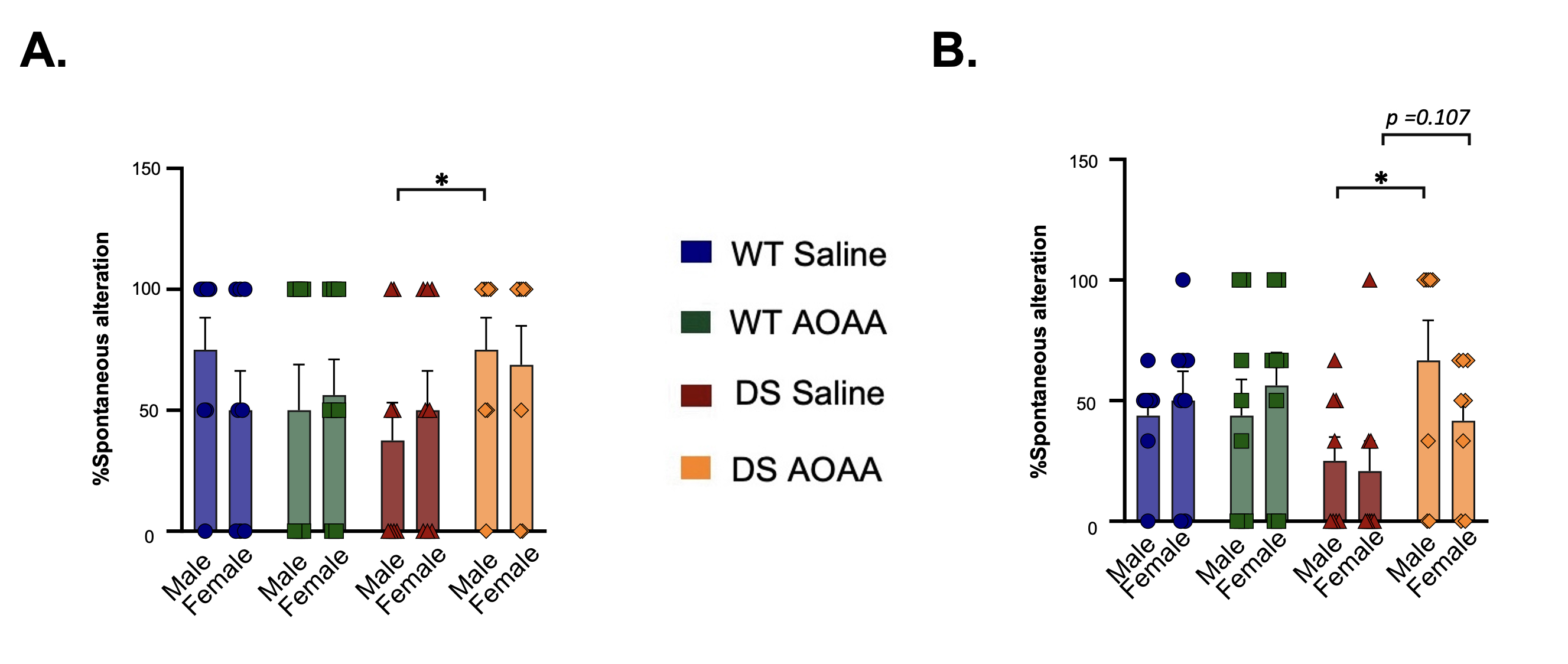

Supplement: Supplementary file 15 — Supplementary file15 (JPG 466 KB) [file 11357_2024_1146_MOESM15_ESM.jpg]

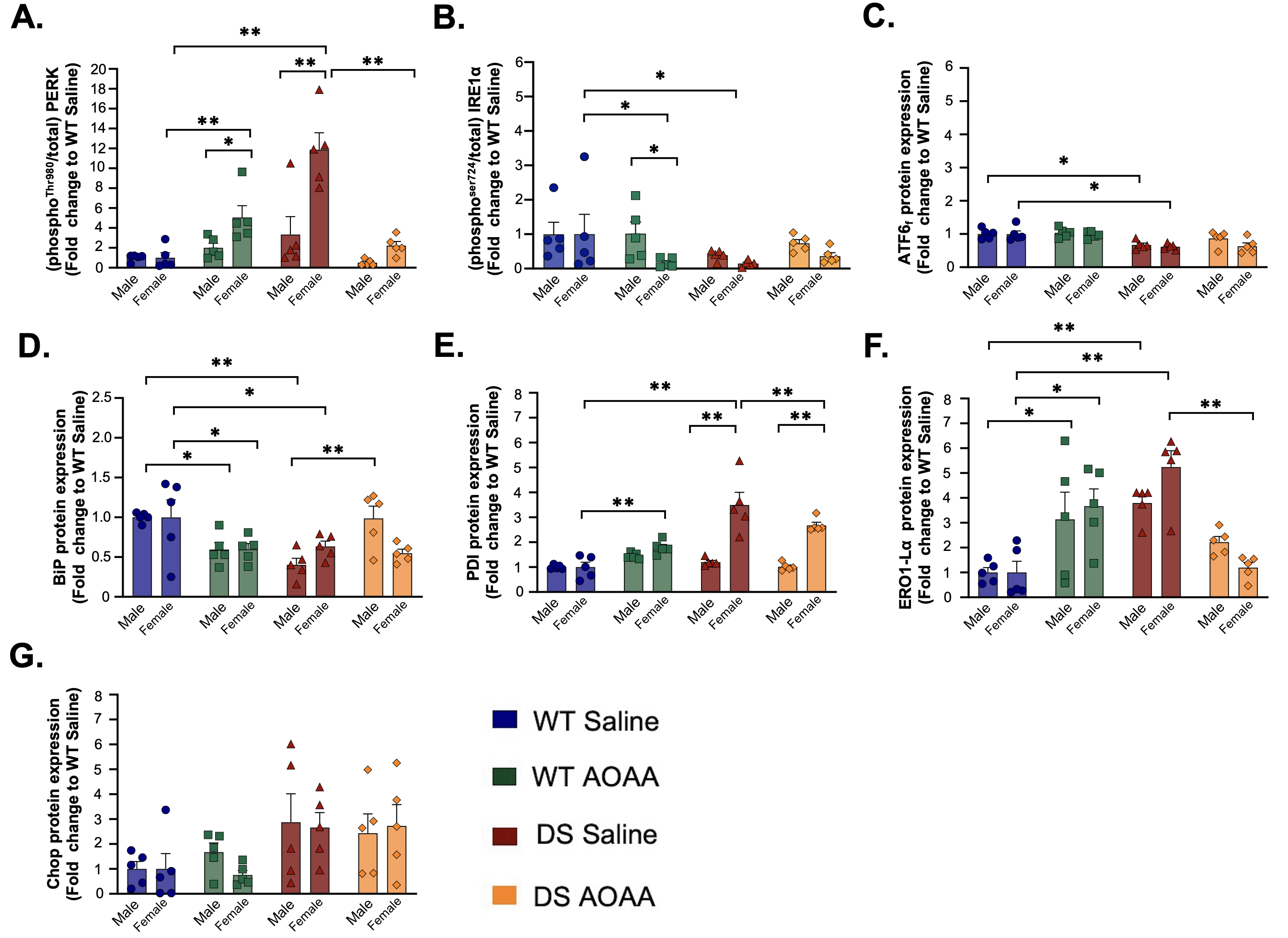

Supplement: Supplementary file 16 — Supplementary file16 (JPG 1060 KB) [file 11357_2024_1146_MOESM16_ESM.jpg]

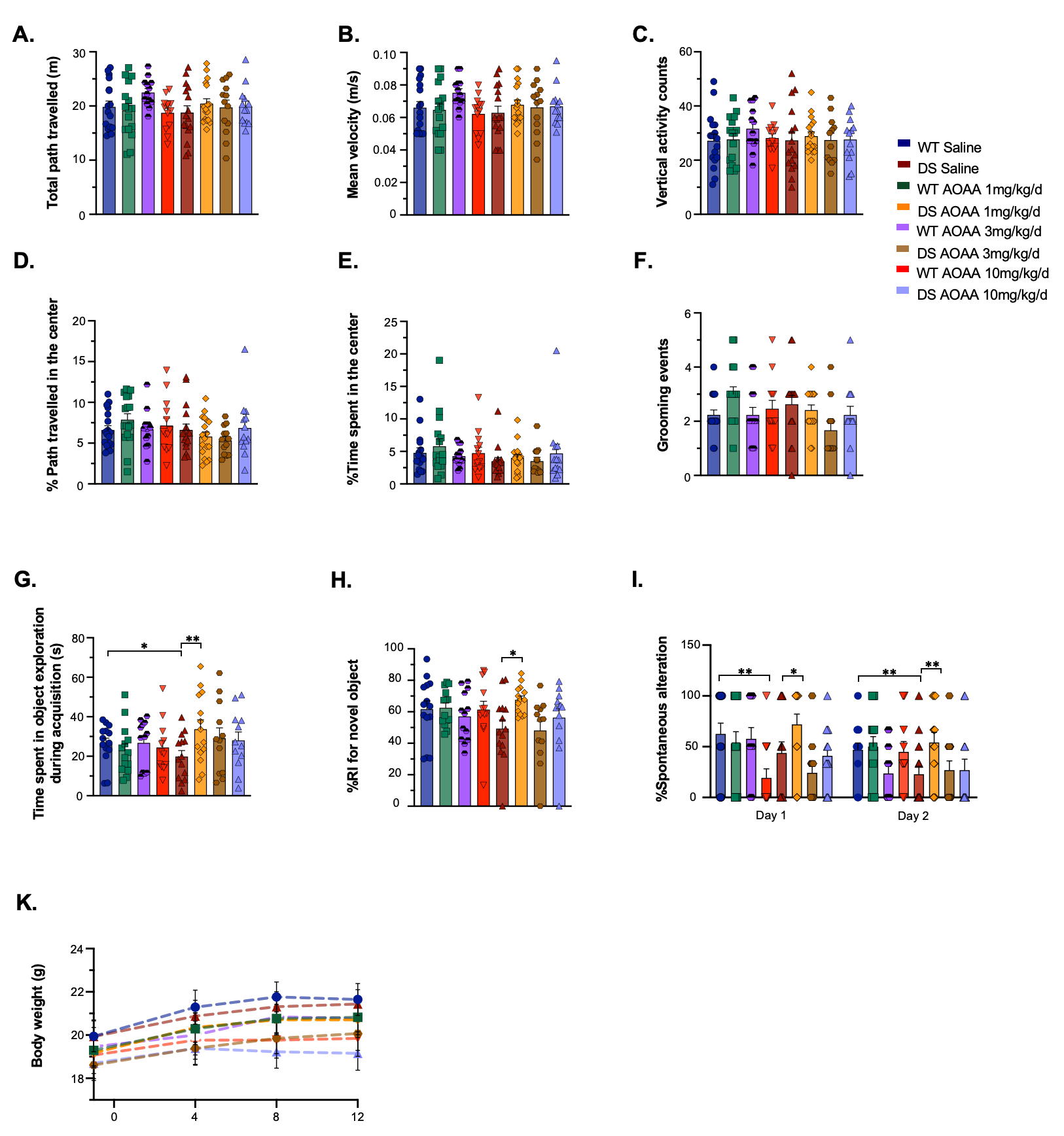

Supplement: Supplementary file 17 — Supplementary file17 (JPG 867 KB) [file 11357_2024_1146_MOESM17_ESM.jpg]
