# Supplementary material for: Neurobehavioral dysfunction in a mouse model of Down syndrome: upregulation of cystathionine β-synthase, H2S overproduction, altered protein persulfidation, synaptic dysfunction, endoplasmic reticulum stress, and autophagy
Source: GeroScience. 2024 Apr 1;46(5):4275–314. doi: 10.1007/s11357-024-01146-8 (PMC11336008; doi:10.1007/s11357-024-01146-8)
Supplement: Supplementary file 18 — Supplementary file18 (DOCX 14 KB) [file 11357_2024_1146_MOESM18_ESM.docx]

Table S11: Primers used for animal genotyping.

| Targeted Region | Ref ID | Sequence 5’ 🡪 3’ | Primer type |
| --- | --- | --- | --- |
| Duplication | 15359 | GGA GCC AGG GCT GAT GGT | Mutant Forward |
|  | 15360 | CAA CGC GGC CTT TTT ACG | Mutant Reverse |
| Wild-type allele specific for 3’-part of the targeted locus | oIMR7338 | CTA GGC CAC AGA ATT GAA AGA TCT | Internal Positive Control Forward |
|  | oIMR7339 | GTA GGT GGA AAT TCT AGC ATC ATC C | Internal Positive Control Reverse |
